# Extracellular Vesicle‐Mediated Regulation of H3C14 Contributes to Gemcitabine Resistance in Bladder Cancer

**DOI:** 10.1002/jev2.70179

**Published:** 2025-10-29

**Authors:** Cheng‐Shuo Huang, Dah‐Shyong Yu, Shih Sheng Jiang, Ying‐Si Wu, Jar‐Yi Ho, Cheng‐Ping Yu

**Affiliations:** ^1^ Graduate Institute of Life Sciences, College of Biomedical Sciences National Defense Medical University Taipei Taiwan; ^2^ Graduate Institute of Pathology and Parasitology, College of Medicine National Defense Medical University Taipei Taiwan; ^3^ Department of Pathology, Tri‐Service General Hospital National Defense Medical University Taipei Taiwan; ^4^ National Institute of Cancer Research National Health Research Institutes Miaoli Taiwan; ^5^ Department of Integrative Biology and Pharmacology, McGovern Medical School the University of Texas Health Science Center at Houston Houston Texas USA; ^6^ Division of Urology, Department of Surgery Tri‐Service General Hospital National Defense Medical University Taipei Taiwan

**Keywords:** extracellular vesicles, gemcitabine resistance, histone protein, tumour microenvironment

## Abstract

Extracellular vesicles (EVs) are critical mediators of intercellular communication within the tumour microenvironment and play a significant role in drug resistance. We aimed to investigate the mechanisms underlying gemcitabine (GCB) resistance in bladder cancer. GCB‐resistant bladder cancer cells exhibited dysregulation of nucleoside‐metabolizing enzymes and transporters. Characterization of EV subpopulations derived from GCB‐resistant cells revealed their ability to transfer drug‐resistant phenotypes to naïve cancer cells by modulating intracellular levels of nucleoside metabolic proteins and transporters. Proteomic and transcriptomic analyses identified the histone protein H3.2 and its corresponding transcript, H3C14, as key regulators in the transmission of GCB resistance. Notably, H3C14 overexpression in resistant cells restored GCB sensitivity, whereas its knockdown induced GCB resistance. Rab27A‐mediated biogenesis and secretion emerged as a crucial mechanism regulating EV release and H3C14 excretion in GCB‐resistant cells. A specific EV subpopulation enriched in CD147 and LAMB1—referred to as Excretion EVs—carried H3.2 (H3C14) but did not induce GCB resistance in recipient cells, suggesting their primary role in eliminating proteins associated with tumour progression and drug resistance. These findings highlight the role of EV‐mediated H3C14 excretion in regulating GCB resistance and suggest potential therapeutic strategies targeting EV pathways to overcome drug resistance in bladder cancer.

## Graphical abstract

H3C14 modulates gemcitabine metabolism and is selectively excluded via extracellular vesicles (EVs) in gemcitabine (GCB)‐resistant bladder cancer. CD9⁺/CD63⁺/CD81⁺ EVs (Transport‐EVs) transmit resistance, whereas CD147⁺/LAMB1⁺ EVs (Excretion‐EVs) excrete tumour‐suppressive cargos, highlighting the functional divergence of EV subtypes in GCB resistance.

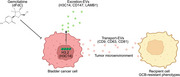

## 1 Introduction

Bladder cancer is the seventh most common malignancy worldwide and the second most prevalent tumour of the urinary tract after prostate cancer. It is broadly classified into muscle‐invasive bladder cancer (MIBC) and non‐muscle‐invasive bladder cancer (NMIBC), with NMIBC accounting for approximately 75% of newly diagnosed cases (Richters et al. 2020; Zhu et al. 2021; Yu et al. 2018). Despite initial therapeutic interventions, over 25% of NMIBC cases eventually progress to MIBC or metastatic disease, resulting in poor clinical outcomes (Babjuk 2018; Sastry and Kellie 2005). Surgical resection remains a cornerstone of treatment for bladder cancer: transurethral resection is typically performed for NMIBC treatment, whereas radical cystectomy is the standard therapy for MIBC. However, in patients with MIBC, the 5‐year post‐surgical survival rate remains <50% (Kang et al. 2020). Since the 1980s, cisplatin‐based neoadjuvant chemotherapy (NAC) has been introduced to improve survival outcomes, often in combination with immunotherapies, such as Bacillus Calmette‐Guérin or interleukin‐15 receptor agonists (Porter et al. 2011; Ward Grados et al. 2022). Despite its clinical benefits, the use of cisplatin is significantly limited owing to its severe adverse effects, including nephrotoxicity, peripheral neuropathy and myelosuppression (Tsang et al. 2009; Gold and Raja 2023; DeGeorge et al. 2017; von der Maase et al. 2005).

To address these limitations, gemcitabine (GCB)‐based regimens have emerged as safer and more tolerable first‐line treatments for bladder cancer. Among NAC protocols, gemcitabine + cisplatin (GC), dose‐dense methotrexate, vinblastine, doxorubicin and cisplatin (ddMVAC), and gemcitabine + paclitaxel (GP) are commonly used. Clinical studies have demonstrated that GC provides survival outcomes comparable to or better than those of MVAC, with significantly reduced toxicity, making it a preferred option for patients with advanced or metastatic urothelial carcinoma (von der Maase et al. 2000; Tae et al. 2022). Additionally, the GP regimen offers an effective alternative for patients ineligible for cisplatin‐based therapy because of comorbidities or impaired renal function (Pfister et al. 2021), (Takahashi et al. 2006). Despite these advances, chemoresistance remains a significant challenge, limiting the long‐term efficacy of GCB‐based treatments and contributing to tumour progression. Therefore, an improved understanding of the molecular mechanisms underlying GCB resistance is essential for developing more effective and durable therapeutic strategies.

GCB is a nucleoside analogue that exerts cytotoxic effects by incorporating its active metabolite, difluorodeoxycytidine triphosphate (dFdCTP), into DNA, resulting in replication obstruction and apoptosis (Zeng et al. 2019; Hayashi et al. 2011; Han et al. 2021). However, tumour cells often develop adaptive mechanisms to evade these cytotoxic effects. Resistance is commonly associated with dysregulation of metabolizing enzymes and nucleoside transporters, including the downregulation of deoxycytidine kinase (DCK) (Bergman et al. 2002; Patel et al. 2017) and thymidine kinase 1 (TK1) (Malvi et al. 2019; Beauséjour et al. 2002), both of which are crucial for GCB activation. Conversely, the upregulation of cytosolic 5'‐nucleotidase 2 (NT5C2) and concentrative nucleoside transporter 3 (CNT3) has been observed (Alvarellos et al. 2014; Zhang et al. 2007; Liu et al. 2014; Ueno et al. 2007), further impairing the intracellular conversion and efficacy of GCB. Additionally, the reduced expression of equilibrative nucleoside transporter 1 (ENT1) and CNT1 limits GCB uptake into tumour cells, contributing to drug resistance (Jia and Xie 2015; Greenhalf et al. 2014).

Beyond intracellular metabolic changes, GCB resistance is increasingly recognized as a major driver of bladder cancer recurrence and progression (Jia and Xie 2015). In NMIBC, multiple recurrences are associated with a higher progression risk and often require more aggressive interventions (Sharma et al. 2023). Transcriptomic studies have revealed that recurrent and multifocal bladder tumours share similar gene expression profiles, suggesting that paracrine or autocrine signalling within the tumour microenvironment may contribute to post‐chemotherapy recurrence (Lindgren et al. 2008; Habuchi 2005). These observations support the hypothesis that GCB‐resistant phenotypes may be transferred from resistant donor cells to naïve, non‐resistant recipient cells, thereby promoting tumour progression. However, the precise mechanisms underlying this phenotypic transfer remain unclear.

Extracellular vesicles (EVs), particularly exosomes, have emerged as key mediators of intercellular communication, playing critical roles in tumour progression and drug resistance. These vesicles transport bioactive molecules—proteins, lipids and nucleic acids—that affect the behaviour of recipient cells within the tumour microenvironment (Meng et al. 2019; Sun et al. 2018; Mashouri et al. 2019). In bladder cancer, tumour‐derived EVs have been implicated in oncogenic signalling through the transfer of microRNAs [e.g., miR‐222‐3p (Liu et al. 2021), miR‐106b (Fang et al. 2019)], matrix metalloproteinases [e.g., MMP14 (Li et al. 2022)] and receptor tyrosine kinases [e.g., EphA2 (Fan et al. 2018)]. These molecules contribute to drug resistance and promote a more aggressive tumour phenotype in recipient cells (Teoh et al. 2022). Therefore, understanding the role of EV‐mediated resistance is essential for identifying novel therapeutic targets for disrupting these resistance pathways (Di Donato et al. 2023). Based on this rationale, we aimed to investigate the role of EV‐mediated GCB resistance in bladder cancer.

## 2 Materials and Methods

### 2.1 Cell Lines and Cell Culture

Human bladder cancer cell lines T24, J82 and 5637 were obtained from the American Type Culture Collection and the Bioresource Collection and Research Centre. Cells were cultured in Roswell Park Memorial Institute (RPMI)‐1640 medium (Life Sciences, Palo Alto, CA, USA) supplemented with 5% foetal bovine serum (FBS) and 1 µg/mL penicillin–streptomycin (Life Sciences, Palo Alto, CA, USA). All cells were maintained at 37°C in a humidified incubator with 5% CO_2_. To generate GCB‐resistant cell lines, T24 and 5637 cells were continuously exposed to 0.01 µM GCB hydrochloride (Sigma–Aldrich, G6423), a concentration corresponding to their IC_50_ values, for a period 3 months. Following this selection phase, those cells were cultured for an additional 3 months in GCB‐free medium and subsequently re‐evaluated GCB cytotoxicity in a serial concentrations of GCB for ensuring the stability of the GCB‐resistant phenotype. The resulting resistant sublines, designated as T24GCB and 5637GCB, were maintained in GCB‐containing medium to preserve their drug‐resistant phenotype.

### 2.2 Isolation of EVs From Conditioned Media (ConMed)

To isolate EVs, 5 × 10⁶ cells were seeded into T75 flasks and cultured in 10 mL of RPMI‐1640 medium supplemented with 5% EV‐depleted FBS (System Biosciences (McNamara et al.), Palo Alto, CA, USA) and 1 µg/mL penicillin‐streptomycin (Life Sciences). After 48 h, ConMed was collected from T24, T24GCB, 5637, 5637GCB and J82 cell cultures for EV isolation. EVs were isolated using a standard differential centrifugation protocol consisting of sequential centrifugation at 3000 × *g* for 30 min to remove cells and large debris, followed by 10,000 × *g* for 1 h to eliminate apoptotic bodies and large vesicles. Thereafter, the supernatant was filtered through a 0.22 µm membrane to remove residual contaminants. Finally, ultracentrifugation at 120,000 × *g* for 2 h at 4°C was performed to pellet the EVs. The resulting EV pellets were resuspended in 0.02 µm‐filtered phosphate‐buffered saline (PBS) and stored at −80°C for further analysis. For the MTT assay, T24 and 5637 cells were co‐cultured with the indicated EVs or ConMed for 24 h. For the colony formation assay, T24 and 5637 cells were co‐cultured with the indicated EVs for 14 days. For protein extraction, EV pellets were resuspended in 100 µL of radioimmunoprecipitation assay (RIPA) lysis buffer (Thermo Fisher Scientific, Waltham, MA, USA) containing a protease inhibitor cocktail (Roche, Basel, Switzerland) at a 1:100 dilution. Samples were lysed at –80°C for 24 h prior to western blot analysis.

### 2.3 Nanoparticle Tracking Analysis (NTA)

EV size distribution and concentration were analysed using the NanoSight NS300 system (Malvern Panalytical, UK). EV samples were resuspended in 0.02 µm‐filtered PBS at a concentration of 1 µg/mL and further diluted 100‐fold to achieve an optimal detection range of 50–100 particles per frame. For analysis, samples were manually injected into the sample chamber at ambient temperature. Data acquisition was performed using a 488‐nm laser and a high‐sensitivity scientific complementary metal‐oxide semiconductor camera. Each sample was analysed in triplicate under the same conditions. The detection threshold was set to seven for all measurements. All data were analysed using NTA 3.0 analytical software, with consistent detection threshold settings applied across all measurements to ensure consistency.

### 2.4 Transmission Electron Microscopy (TEM)

EV morphology was assessed using TEM. The EV pellet was resuspended in a fixation buffer containing 2.5% glutaraldehyde (in 0.1 M sodium cacodylate, pH 7.4) and 4% paraformaldehyde (in 1× PBS) and, thereafter, incubated overnight at 4°C. After fixation, samples were washed three times with PBS (10 min each) and post‐fixed in 1% osmium tetroxide (in double‐distilled water) for 50 min at room temperature (25°C). The fixed EVs were embedded in 10% gelatine, further fixed in glutaraldehyde at 4°C, and sectioned into blocks <1 mm^3^. Samples were dehydrated through a graded ethanol series (70%, 90%, 95% and 100%, 10 min per step), followed by a propylene oxide exchange. Specimens were infiltrated with increasing concentrations of Quetol‐812 epoxy resin (25%, 50%, 75% and 100%) for at least 2 h per step; afterward, they were embedded in fresh Quetol‐812 and polymerized at 70°C for 24 h. Ultrathin sections (approximately 70 nm) were cut using a Leica UC6 ultramicrotome and stained sequentially with uranyl acetate for 10 min and lead citrate for 5 min at room temperature (25°C). TEM images were acquired using the Hitachi HT7700 TEM (Hitachi, Japan) operated at 120 kV.

### 2.5 ImageStreamX, Fluorescence Microscopy and Dynamic Uptake of EVs

Bladder cancer cells were treated with 0.1 µM GCB for 48 h or incubated with EVs derived from various sources—T24‐EVs, T24GCB‐EVs, 5637‐EVs, 5637GCB‐EVs, or J82‐EVs—for 6 h. Following incubation, 3 × 10⁶ cells were washed with cold PBS, fixed in 4% paraformaldehyde at 4°C for 1 h, and rehydrated in a permeabilization buffer containing PBS, 1% bovine serum albumin and 0.1% Triton X‐100. After centrifugation, the cells were incubated overnight at 4°C with the following primary antibodies: mouse monoclonal H3C14 monoclonal (1:500, LSBio, Beijing, China) and rabbit polyclonal Rab27A (1:800, Cell Signalling Technology, Danvers, MA, USA). After washing with PBS, cells were incubated at 4°C for 2 h in the dark with secondary antibodies: Alexa Fluor 488‐conjugated goat anti‐mouse IgG and Alexa Fluor 647‐conjugated goat anti‐rabbit IgG (both at 1:2000 dilution, Thermo Fisher Scientific, Waltham, USA). Nuclear staining was performed using 5 µM 4',6‐diamidino‐2‐phenylindole (DAPI) [Thermo Fisher Scientific, Waltham, USA]. Cells were analysed using the ImageStreamX system (Luminex), equipped with a 40× objective and the extended depth of field feature. Fluorescent signals were detected at 405, 488 and 645 nm, and brightfield imaging was used for granularity and morphological analysis. Image acquisition was conducted at a rate of 10–50 cells/s, with a minimum of 5000 cells acquired per sample. Single‐stained controls (H3C14‐AF488, Rab27A‐AF647 and DAPI) were used to generate a compensation matrix, calculated using IDEAS 5.0 software.

### 2.6 EV Detection and Dynamic Uptake Assay

EVs were labelled with the following fluorophore‐conjugated primary antibodies: CD9‐APC (1:2000, Miltenyi Biotec), CD63‐FITC (1:2000, Miltenyi Biotec), CD81‐PE (1:2000, Miltenyi Biotec), CD147‐PE/Cyanine7 (1:2000, BioLegend, San Diego, CA, USA) and LAMB1‐APC/Cyanine7 (1:2000, BioLegend). Labelling was performed at 4°C for 2 h. To eliminate unbound antibodies, EVs were subjected to ultracentrifugation at 120,000 × *g* for 2 h at 4°C, washed with 0.02 µm filtered PBS and ultracentrifuged again under the same conditions. The resulting EV–antibody complexes were resuspended in 0.02 µm filtered PBS and analysed using imaging flow cytometry or fluorescence microscopy to assess EV surface marker profiles. For dynamic uptake assays, T24 or 5637 bladder cancer cells were incubated with the fluorescently labelled EV‐antibody complexes for 4 h before imaging.

### 2.7 Quantification of GCB via High‐Performance Liquid Chromatography (HPLC)

GCB concentrations in biological samples were quantified using HPLC, based on a modified protocol by Prasath et al. (2025). The method was optimized for sensitivity, linearity and reproducibility. Chromatographic separation was performed in a C18 reverse‐phase column (100 × 4.6 mm, 5 µm particle size) at room temperature. The mobile phase comprised 20 mM sodium phosphate buffer (pH 6.5) and acetonitrile at a ratio of 95:5 (v/v). The mobile phase was filtered through a 0.22 µm membrane and degassed prior to use. The flow rate was maintained at 1.0 mL/min, with an injection volume of 20 µL. GCB was detected at 210 nm using a UV detector, and the total run time per sample was approximately 20 min. Calibration standards were prepared by serial dilution of a 1 mM GCB stock solution in methanol to final concentrations of 0.01, 0.05, 0.1, 0.5, 1, 5 and 10 µM. Calibration curves were generated by plotting peak area versus GCB concentration (µM), yielding excellent linearity across the entire range (R^2^ = 0.9994; y = 6456.4x + 149.06), as shown in Figure S1A,D. Regarding sample preparation, 100 µL of EV or conditioned medium sample was mixed with 400 µL of methanol, vortexed for 30 s and centrifuged at 12,000 rpm for 10 min at 4°C. Although GCB‐spiked EVs, those are extracted EVs treated with GCB for 24 h, were used as positive controls. The resulting supernatant was filtered through a 0.22 µm membrane prior to injection. GCB consistently eluted at 6–8 min. The method demonstrated stable retention times, high recovery rates (95%–105%) and acceptable intra‐ and inter‐assay precision, confirming its suitability for quantitative analysis of GCB in EVs and cell culture media.

### 2.8 Cell Viability Assay

Bladder cancer cell viability was assessed using the 3‐(4,5‐dimethylthiazol‐2‐yl)‐2,5‐diphenyl‐tetrazolium bromide (MTT) assay (Sigma–Aldrich). Cells were seeded in 96‐well plates at 3000 cells/well and incubated overnight under standard culture conditions. The following day, cells were treated with serial dilutions of GCB for 48 h. After treatment, 0.1 mg/mL MTT solution was added to each well, and cells were incubated for 3 h. The resulting formazan crystals were dissolved in dimethyl sulfoxide (DMSO, Sigma‐Aldrich) at room temperature (25°C) for 10 min. Absorbance was measured at 560 nm using a spectrophotometer (Bio‐Rad Inc., Hercules, CA, USA). All experiments were performed in sextuplicate (*n* = 6 per condition) to ensure statistical reliability.

### 2.9 Transwell Migration and Invasion Assays

Transwell migration and invasion assays were conducted using 8‐µm pore size Transwell inserts (Corning, Steuben County, NY, USA). For each condition, 5 × 10⁴ cells were seeded into the upper chambers in a serum‐free medium, either treated with GCB (0.1 µM) or transfected with siH3C14. For invasion assays, the inserts were pre‐coated with 1 mg/mL Matrigel (BD Biosciences, Franklin Lakes, NJ, USA) and incubated at 37°C for 1 h before cell seeding. Cells were allowed to migrate or invade toward the lower chamber containing a medium supplemented with 10% FBS for 24 h (migrate) or 48 h (invade) in a humidified 5% CO_2_ incubator at 37°C. After incubation, cells on the upper surface of the membranes were carefully removed with a cotton swab. The migrated or invaded cells on the bottom side were fixed with 4% paraformaldehyde and stained with 0.5% crystal violet (Sigma‐Aldrich) for 10 min at room temperature (25°C). The membranes were mounted on glass slides, and five randomly selected fields per insert were imaged using a light microscope at 100× magnification. Quantification was performed by measuring the stained area using ImageJ software. All experiments were performed in triplicate (*n* = 3 per condition) to ensure reproducibility.

### 2.10 Colony Formation Assay

The clonogenicity of bladder cancer cells was assessed following gene manipulation (siH3C14, siRab27A or H3C14‐GFP overexpression) or treatment with 5 × 10⁹ particles/mL EVs isolated from T24, T24GCB, 5637, 5637GCB and J82 cells. Briefly, 1 mL of 0.5% agarose in complete RPMI 1640 medium was used as the bottom layer in a 3.5 cm dish. A suspension of 1 × 10⁴ cells in 0.3% agarose was prepared in RPMI 1640 medium and layered on top. Cells were incubated at 37°C in a humidified 5% CO_2_ incubator for 14 days, with fresh RPMI 1640 medium added every 3 days. At the endpoint, colonies were fixed and stained with crystal violet (Sigma–Aldrich) for 10 min, followed by destaining with tap water for 20 min. Colonies were quantified using ImageJ software, and all experiments were performed in triplicate (*n* = 3 per condition) to ensure reproducibility.

### 2.11 Western Blotting

Total protein was extracted from cultured cells and EVs isolated by ultracentrifugation using RIPA lysis buffer (Thermo Fisher Scientific, Waltham, MA, USA) supplemented with a protease inhibitor cocktail (Roche, Basel, Switzerland) at a 1:100 dilution ratio. Lysates were centrifuged at 12,000 × *g* for 10 min to remove insoluble material. Protein concentration was quantified using the bicinchoninic acid protein assay kit (Thermo Fisher Scientific, Waltham, MA, USA). For electrophoresis, 30 µg protein was resolved on a 10% sodium dodecyl sulphate‐polyacrylamide gel electrophoresis and transferred to a polyvinylidene fluoride membrane (Millipore, Burlington, MA, USA). Membranes were blocked with 5% non‐fat milk or 2% albumin (BioShop, Burlington, Ontario, Canada) in tris‐buffered saline with Tween 20 (TBST) buffer and incubated overnight at 4°C with primary antibodies (detailed in Table S1). After primary antibody incubation, membranes were washed with TBST and incubated with horseradish peroxidase (HRP)‐conjugated secondary antibodies (1:5000; Santa Cruz Biotechnology, Dallas, TX, USA) for 1 h at room temperature (25°C). Immunoreactive bands were visualized using the Immobilon Western Chemiluminescent HRP Substrate (Millipore, Burlington, MA, USA) and analysed using the ultra‐violet products GelStudio PLUS System (Analytik Jena AG, Thuringia, Germany) and ImageJ software. Each experiment was performed in triplicate (*n* = 3 per condition) to ensure reproducibility.

### 2.12 RNA Sequencing

Total RNA was extracted from GCB‐sensitive (T24 and 5637) and GCB‐resistant (T24GCB and 5637GCB) bladder cancer cells, as well as from their corresponding EVs, using the TOOLSmart RNA Extractor (BIOTOOLS, Taiwan) according to the manufacturer's instructions. RNA integrity and concentration were assessed using Agilent 2100 Bioanalyzer. RNA libraries were prepared with the TruSeq Stranded mRNA Library Prep Kit and sequenced on an Illumina NovaSeq 6000 platform to generate 150 bp paired‐end reads. Raw sequencing data were quality‐checked using FastQC and aligned to the human reference genome (GRCh38) with HISAT2. Transcript abundance was quantified using StringTie and expressed as Fragments Per Kilobase of transcript per Million mapped reads. Differential gene expression analysis was conducted using DESeq2. To identify signalling pathways associated with GCB resistance, gene set enrichment analysis and Ingenuity Pathway Analysis (IPA; Qiagen) were performed.

### 2.13 Quantitative Real‐Time Polymerase Chain Reaction (qRT‐PCR)

Total RNA was extracted from cultured cells and EVs using the TOOLSmart RNA Extractor (BIOTOOLS, Taiwan), following the manufacturer's instructions. RNA concentration and purity were assessed using the NanoDrop spectrophotometer (Thermo Fisher Scientific, Waltham, MA, USA). Reverse transcription (RT) was performed using the FIREScript RT complementary DNA (cDNA) Synthesis Mix RT Kit with Oligo(dT) primers (Solis BioDyne, Tartu, Estonia). Each 20 µL RT reaction contained 1000 ng of total RNA, 2 µL of 10× RT Reaction Premix, 1.5 µL of FIREScript Enzyme Mix and RNase‐free distilled water. The reactions were incubated at 37°C for 20 min, followed by enzyme inactivation at 85°C for 5 min. qRT‐PCR was conducted using the QuantStudio 5 Real‐Time PCR System (Applied Biosystems, Foster City, CA, USA), following the manufacturer's instructions. Each 20 µL qPCR reaction contained 4 µL of diluted cDNA, 4 µL of 5× HOT FIREPol EvaGreen qPCR Mix Plus (ROX) (Solis BioDyne, Tartu, Estonia), 0.5 µL of gene‐specific forward and reverse primers (10 pmol/µL) and 11 µL of RNase‐free distilled water. The PCR cycling protocol was as follows: initial denaturation at 95°C for 12 min, followed by 50 amplification cycles comprising denaturation at 95°C for 15 s, annealing at 58–60°C for 20 s and extension at 72°C for 20 s. Amplicon specificity was confirmed using melting curve analysis. Glyceraldehyde‐3‐phosphate dehydrogenase served as an internal reference gene. Relative gene expression levels were calculated using the 2^−ΔΔCT^ method. No‐template controls were included in each run to monitor for contamination, and all reactions were conducted in triplicate (*n* = 3) to ensure reproducibility. Primer sequences used for qRT‐PCR are provided in Table S2.

### 2.14 Immunohistochemistry

Immunohistochemistry was performed on formalin‐fixed, paraffin‐embedded bladder cancer tissue specimens obtained from enrolled patients or xenograft tumour sections. Briefly, 4‐µm tissue sections were deparaffinized, rehydrated and subjected to antigen retrieval in citrate buffer (pH 6.0) at 95°C for 20 min. After cooling to room temperature (25°C), sections were blocked with 10% goat serum for 1 h and then incubated with primary antibodies against H3C14 or CNT3 (diluted 1:200) for 2 h at room temperature (25°C). Following three washes with TBST (10 min each), antigen‐antibody complexes were visualized using the Super Sensitive Polymer‐HRP Detection System with 3,3'‐diaminobenzidine (DAB) chromogen (BioGenex, San Francisco, USA). Sections were then counterstained with haematoxylin, dehydrated and mounted. Immunoreactivity was quantified using Aperio ImageScope and Spectrum version 12.0 software for each specimen.

### 2.15 H3C14 Fluorescence Assay

For immunofluorescence assay of H3C14, bladder cancer cells were adjusted to a concentration of 2 × 10⁵ cells/mL, seeded onto glass coverslips, and allowed to adhere overnight. The cells were then fixed with 4% paraformaldehyde for 20 min, permeabilized with 0.5% Triton X‐100 (v/v) in PBS for 20 min and blocked with 10% BSA (v/v) in PBS for 1 h at room temperature (25°C). Cells were incubated overnight at 4°C with a mouse monoclonal anti‐H3C14 antibody (1:500 dilution; LSBio, Beijing, China), followed by incubation with an Alexa Fluor 488‐conjugated goat anti‐mouse secondary antibody (1:500; Thermo Fisher Scientific, Waltham, USA) for 1 h at room temperature (25°C) in the dark. After counterstaining with DAPI (Sigma‐Aldrich, USA), the coverslips were mounted and fluorescence signals were visualized using an LSM980 Multiphoton laser scanning confocal microscope (Carl Zeiss, Oberkochen, Germany). The number of H3C14‐positive foci was quantified using ImageJ software.

### 2.16 Knockdown and Overexpression of H3C14 and Rab27A

#### 2.16.1 Small interfering RNAs (siRNA) Knockdown of H3C14, Rab27A and CNT3

siRNAs targeting H3C14 (siH3C14), Rab27A (siRab27A) and CNT3 (siCNT3), along with their corresponding negative control siRNAs, were purchased from Dharmacon (GE Healthcare Dharmacon, Lafayette, CO, USA). Bladder cancer cells were transfected with 40 pmol siRNA oligonucleotides using DharmaconFECT transfection reagents (GE Healthcare Dharmacon, Lafayette, CO, USA), following the manufacturer's protocol. The specific transfections were performed as follows: T24 cells with siH3C14, T24GCB cells with siRab27A and J82 cells with siRab27A. After 48 h post‐transfection, cells were collected for RNA and protein extraction or subjected to downstream functional assays. Knockdown efficiency was evaluated using RT‐qPCR and western blot analysis.

#### 2.16.2 H3C14 Overexpression Plasmid Transfection

The H3C14‐GFP plasmid was obtained from Sino Biological (Beijing, China). T24GCB and J82 bladder cancer cells were transfected with 2 µg of the H3C14‐GFP plasmid using DharmaconFECT transfection reagents, following the manufacturer's instructions. After 48 h, cells were collected for downstream analyses, including RT‐qPCR, western blotting and functional assays. Protein band intensities from western blots were quantified using ImageJ software, and all experiments were performed in triplicate.

### 2.17 Flow Cytometric Analysis of Apoptosis

Apoptosis was evaluated using Annexin V‐FITC/propidium iodide (PI) dual staining. T24 or 5637 cells were transfected with siH3C14 or negative control siRNA for 48 h, followed by treatment with GCB (0.01 µM) for an additional 48 h. After treatment, cells were harvested, washed twice with cold PBS, and resuspended in 1× binding buffer (BD Biosciences) at a density of 1 × 10⁶ cells/mL. Annexin V‐FITC (5 µL) and PI (5 µL) were added to 100 µL of the cell suspension, incubated for 15 min at room temperature (25°C) in the dark, and subsequently diluted with 400 µL of binding buffer. Samples were analysed using a BD Accuri C6 flow cytometer. At least 10,000 events were recorded per sample, and data were processed using FlowJo software (BD Biosciences). The percentages of early apoptotic (Annexin V⁺/PI^−^), late apoptotic (Annexin V⁺/PI⁺) and viable (Annexin V^−^/PI^−^) cells were quantified. Each condition was performed in triplicate and repeated in three independent experiments

### 2.18 Xenograft

Eight‐week‐old female Balb/c nude mice were obtained from the National Laboratory Animal Centre (Taiwan) and acclimated for 1 week before experimentation. Prior to implantation, T24GCB‐Vector and T24GCB‐H3C14 cells were tested for mycoplasma contamination using the e‐Myco Mycoplasma PCR Detection Kit (Intron, Korea). After confirmation of the absence of contamination, 5 × 10⁶ cells suspended in 100 µL of PBS mixed with 10 mg/mL Matrigel (BD Biosciences, Franklin Lakes, NJ, USA) were subcutaneously implanted into the left flank of each mouse. Once tumours reached approximately 20 mm^3^, mice were randomly assigned to one of four treatment groups: (1) T24GCB‐Vector + PBS [control; 100 µL intraperitoneal (Amrutkar & Gladhaug) injection], (2) T24GCB‐Vector + GCB (1 mg/kg i.p. injection), (3) T24GCB‐H3C14 + PBS (control; 100 µL i.p. injection) and (4) T24‐GCB‐H3C14 + GCB (1 mg/kg i.p. injection). GCB was administered three times per week for 4 weeks. tumour volumes were measured every 3 days using callipers and calculated using the formula: tumour volume = (length × width^2^)/2. On Day 28, all mice were euthanized, and tumours were excised, weighed and fixed in formalin for subsequent immunohistochemical analysis.

### 2.19 Co‐Immunoprecipitation (Co‐IP)

T24GCB cells were treated with GCB (0.1 µM) for 24 h and lysed in IP lysis buffer (20 mM Tris‐HCl, 150 mM NaCl, 1% NP‐40, 1 mM EDTA, supplemented with protease inhibitor cocktail). For each immunoprecipitation, 1 mg of total protein was incubated overnight at 4°C with 2 µg of anti‐H3C14 antibody. Protein A/G magnetic beads (Thermo Scientific) were added to capture the immune complexes, followed by five washes with lysis buffer. Complexes were eluted by boiling in sodium dodecyl sulfate sample buffer and subjected to immunoblotting for CNT3 or Rab27A using ECL detection. Input lysates (5%–10%) were included as loading controls, and IgG isotype controls were used to confirm the specificity of the immunoprecipitation.

### 2.20 Magnetic Bead‐Based Isolation of EV Subpopulations

EVs were isolated using magnetic bead‐based separation kits from Miltenyi Biotec, following the manufacturer's protocol. Specifically, the following kits were used to enrich distinct EV subpopulations: CD9 (130‐110‐913), CD63 (130‐110‐918), CD81 (130‐110‐914) and Pan‐EV (130‐110‐912). For each sample (up to 2 mL of pre‐cleared EV‐containing supernatant), 50 µL of the respective EV Isolation MicroBeads were added and vortexed briefly. The mixture was incubated for 1 h at room temperature (25°C) with gentle mixing. Afterward, magnetically labelled EVs were subjected to column‐based magnetic separation using µ Columns placed in a µMACS Separator attached to a MultiStand (Miltenyi Biotec). Columns were equilibrated with 100 µL Equilibration Buffer, followed by three washes with 100 µL Isolation Buffer. Labelled samples were applied to the columns and allowed to pass through by gravity. Thereafter, columns were washed with four aliquots of 200 µL Isolation Buffer to remove unbound material. To obtain lysed EVs for downstream western blot analysis, the columns were removed from the magnetic separator and placed over 1.5 mL collection tubes. Further, 100 µL of EV lysis and elution buffer (provided by the manufacturer) was added directly to the column, and the EV lysates were flushed out by firmly inserting the plunger. Lysed EV samples were used immediately for protein analysis.

### 2.21 Co‐Culture of Cells With Bead‐Isolated EV Subpopulations

Bead‐isolated EVs subpopulations (CD9⁺, CD63⁺, CD81⁺ and Pan‐EVs) were obtained using Miltenyi Biotec EV Isolation Kits. Following magnetic separation and elution in PBS, equivalent amounts of EVs (5 µg of total protein or 5 × 10⁹ particles) were added to GCB‐sensitive bladder cancer cells (T24 or 5637) seeded in 6‐well plates (1 × 10⁵ cells/well). After a 48 h incubation period, cells were re‐seeded into 96‐well plates (3000 cells/well) and treated with serial concentrations of GCB for an additional 48 h. Cell viability was assessed using either the MTT assay or an automated cell counter. Vehicle control (PBS) and a positive control group treated with ultracentrifuged total EVs were included in parallel. All conditions were tested in triplicate and repeated in at least three independent experiments.

### 2.22 Proteomic Profiling and Label‐Free Quantification

EV proteins from 5637/T24 parental and GCB‐resistant cells, as well as bead‐isolated Transport‐EVs and Excretion‐EVs, were analysed using mass spectrometry. Protein identification and label‐free quantification were performed using Proteome Discoverer software (version 2.3, Thermo Fisher Scientific). MS/MS spectra were searched against the SwissProt human database using the Mascot search engine (version 2.5, Matrix Science, London, UK). Search parameters included a precursor mass tolerance of 10 ppm and a fragment ion mass tolerance of 0.5 Da. Two missed trypsin cleavages were allowed. Oxidized methionine and N‐terminal acetylation were set as variable modifications, whereas carbamidomethylation of cysteine was defined as a static modification. Peptide‐spectrum matches were filtered based on high confidence and Mascot rank 1 to ensure a false discovery rate <1%. Proteins identified by a single unique peptide were excluded. Relative quantification was calculated based on the sum of peptide peak areas using the Minora algorithm. Differentially expressed proteins were defined by a fold change ≥2. Identified differential proteins were subjected to Gene Ontology (GO) enrichment analysis to explore functional pathways associated with EV subtype‐specific functions.

### 2.23 Protein A/G Magnetic Bead Pull‐Down EVs Assay

To isolate specific subpopulations of EVs, 1 × 10^12^ EV particles were incubated with 2 µg of Protein A/G magnetic beads pre‐bound with anti‐CD147 or anti‐LAMB1 antibodies at 4°C for 16 h with gentle rotation. Following incubation, the beads were washed five times with PBS and collected using a magnetic separation stand. The bead‐bound EVs were subsequently analysed via western blotting, scanning electron microscopy (SEM), or co‐culture functional assays.

### 2.24 SEM

The bead‐bound EVs were fixed in 2.5% glutaraldehyde (Sigma–Aldrich) in PBS for 1 h at room temperature (25°C). After two washes with PBS, the samples were sequentially dehydrated using graded ethanol concentrations (30%, 50%, 70%, 90% and 99.5%). Ethanol was allowed to evaporate, and the samples were subsequently subjected to critical point drying on glass substrates. Thereafter, dried samples were sputter‐coated with gold–palladium and visualized using a Thermo Scientific Apreo 2S scanning electron microscope.

### 2.25 Statistical Analysis

All statistical analyses were conducted using SPSS version 22.0 (IBM, SPSS, Chicago, IL, USA), Microsoft Excel 2019 and GraphPad Prism version 10.0 (GraphPad Software, La Jolla, CA, USA). Data were presented as mean ± standard deviation (SD) from at least three independent experiments. Comparisons between two groups were performed using Student's *t*‐test, whereas multiple group comparisons were analysed using one‐way analysis of variance followed by Tukey's post hoc test. Kaplan–Meier survival analysis was performed, using the log‐rank test to assess statistical significance. Correlation analyses were conducted using Pearson's correlation coefficient (*r*). All statistical tests were two‐tailed, with significance set at: *p* < 0.05 (*), *p* < 0.01 (**) and *p* < 0.001 (***). Graphs and statistical visualizations were generated using GraphPad Prism version 10.0.

## 3 Results

### 3.1 Establishment of GCB‐Resistant Bladder Cancer Cell Lines

**FIGURE 1:**
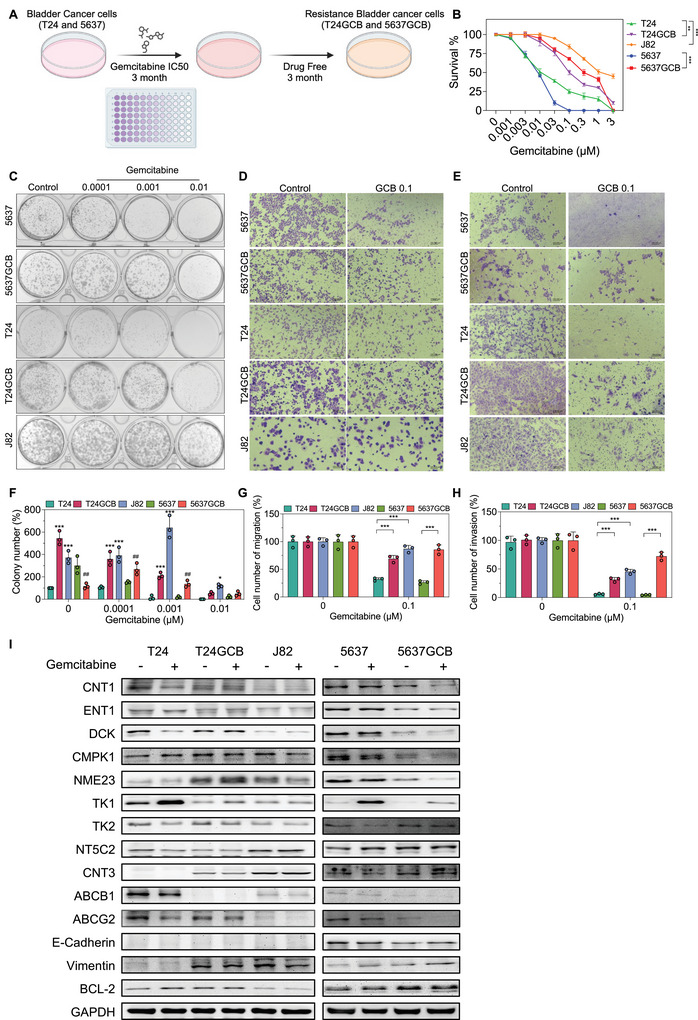
**Characterization of GCB‐resistant bladder cancer cells**. (A) Schematic illustration of the establishment of GCB‐resistant bladder cancer cell lines (T24GCB and 5637GCB). (B) Cell viability of parental (T24, J82 and 5637) and GCB‐resistant (T24GCB and 5637GCB) bladder cancer cells was assessed using the MTT assay following 48 h treatment with a range of GCB concentrations (0, 0.001, 0.003, 0.01, 0.03, 0.1, 0.3, 1 and 3 µM; *n* = 6 per group). (C, F) Colony formation assays were used to evaluate the clonogenic survival of T24, J82, 5637, T24GCB and 5637GCB cells treated with GCB (0, 0.0001, 0.001 and 0.01 µM) for 14 days (*n* = 3 per group). (D, G) Migration assay was used to evaluate the migratory ability of the indicated bladder cancer cells treated with GCB (0 and 0.1 µM) for 24 h (*n* = 3 per group). (E, H) Invasion assay was used to evaluate the invasive potential of bladder cancer cells treated with GCB (0 and 0.1 µM) for 24 h (*n* = 3 per group). (I) Western blot analysis of GCB‐metabolizing enzymes and transporters (CNT1, ENT1, DCK, CMPK1, NME23, TK1, TK2, NT5C2, CNT3, ABCB1 and ABCG2), epithelial‐mesenchymal transition markers (E‐Cadherin and Vimentin) and the anti‐apoptotic protein BCL‐2 in parental and resistant bladder cancer cells following treatment with 0.01 µM GCB for 24 h. Data are presented as mean ± SEM. Statistical significance was determined using an unpaired two‐tailed Student's *t*‐test. *p < 0.05, **p < 0.01, ***p < 0.001. All experiments were performed in triplicate. MTT, 3‐(4,5‐dimethylthiazol‐2‐yl)‐2,5‐diphenyl‐tetrazolium bromide; GCB, gemcitabine.

Given that GCB resistance remains a significant challenge in the treatment of bladder cancer, developing reliable GCB‐resistant cell models is essential for mechanistic studies. To establish GCB‐resistant bladder cancer cell lines, T24 and 5637 cells were continuously exposed to their respective IC_50_ concentrations of GCB for 3 months to generate GCB‐resistant sublines (T24GCB and 5637GCB). Those cells were cultured for an additional 3 months in GCB‐free medium and subsequently re‐evaluated GCB cytotoxicity in a serial concentrations of GCB for ensuring the stability of the GCB‐resistant phenotype (Figure 1A). MTT assays demonstrated that T24GCB and 5637GCB cells exhibited significantly higher survival rates in response to GCB than those of their parental counterparts, particularly at 0.01 µM (Figure 1B). Notably, the intrinsically resistant J82 cell line exhibited greater survival rates at serial GCB concentrations than did T24 and 5637 cells. To evaluate the malignant potential associated with GCB resistance, we performed colony formation, migration and invasion assays. T24GCB, 5637GCB and J82 cells demonstrated higher clonogenic capacity (Figure 1C,F), enhanced migration ability (Figure 1D,G), and increased invasion behaviour (Figure 1E,H) under GCB treatment than those of their parental lines. These results suggest that GCB resistance is associated with a more aggressive cellular phenotype. This observation is consistent with previous findings indicating that chemoresistant bladder cancer cells often exhibit increased invasive and metastatic potential.

Western blot analysis demonstrated a significant reduction in key GCB‐metabolizing enzymes and transporters—ENT1, DCK and TK1—alongside upregulation of several GCB resistance‐associated proteins, including NT5C2 and CNT3, in T24GCB, 5637GCB and J82 cells. Additionally, the anti‐apoptotic protein BCL‐2 was significantly upregulated in T24GCB and 5637GCB cells, suggesting enhanced survival under GCB‐induced stress (Figure 1I). Our established GCB‐resistant cell lines are consistent with previously reported GCB‐resistant models, in which resistance is primarily driven by dysregulation of GCB‐metabolizing enzymes and transporters. This dysregulation leads to insufficient intracellular GCB activation, contributing to therapeutic failure across various GCB‐treated cancers (Amrutkar and Gladhaug 2017).

### 3.2 Characterization and Functional Analysis of EVs in GCB‐Resistant Bladder Cancer Cells

**FIGURE 2:**
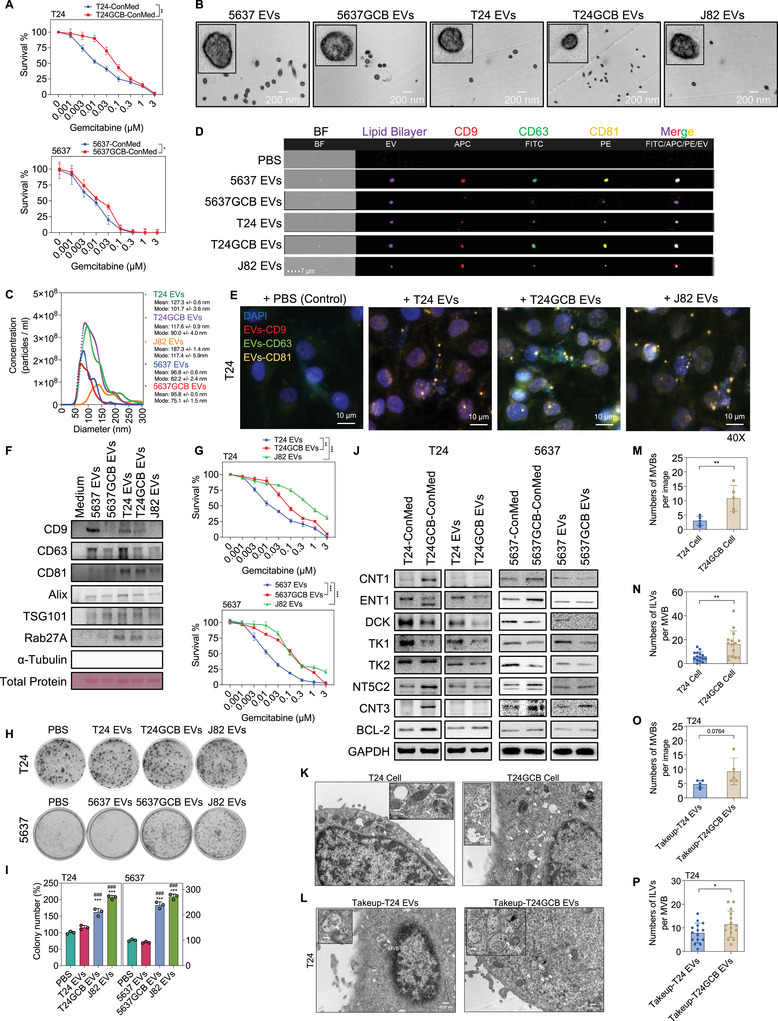
**Characterization and functional analysis of EVs in GCB‐resistant bladder cancer cells**. (A) MTT assay was used to evaluate the viability of T24 and 5637 cells co‐cultured with T24‐ConMed and 5637‐ConMed or T24GCB‐ConMed and 5637GCB‐ConMed for 24 h, followed by GCB treatment, as described in panel E (*n* = 6 per group). (B) Representative transmission electron microscopy images of EVs derived from bladder cancer cells (5637‐EVs, T24‐EVs and J82‐EVs) and their GCB‐resistant counterparts (5637GCB‐EVs and T24GCB‐EVs). Scale bar: 100 nm. (C) Nanoparticle tracking analysis (NS300) was used to evaluate the size distribution and concentration of EVs from 5637, 5637GCB, T24, T24GCB and J82 cells. (D) Imaging flow cytometry analysis of EVs stained with a lipid bilayer dye and labelled with EV surface markers CD9‐APC, CD63‐FITC and CD81‐PE. Each dot represents a single EV. (E) Representative fluorescence microscopy images of T24 cells following a 6‐h incubation with T24‐EVs, T24GCB‐EVs, or J82‐EVs pre‐labelled with CD9‐APC, CD63‐FITC and CD81‐PE. Scale bar: 10 µm. (F) Western blot analysis of EV markers (CD9, CD63, CD81, Alix and TSG101) and endosomal protein Rab27A in EVs from the indicated cell lines. (G) MTT assay evaluating the viability of T24 and 5637 cells co‐cultured with the indicated EVs for 24 h, followed by GCB treatment (0–3 µM) for 48 h (*n* = 6 per group). (H and I) Colony formation assay was used to evaluate the clonogenic potential of T24 and 5637 cells after co‐culture with T24‐EVs, T24GCB‐EVs, or 5637‐EVs, 5637GCB‐EVs, or J82‐EVs for 14 days (*n* = 3 per group). (J) Western blot analysis of GCB‐metabolizing enzymes and transporters (CNT1, ENT1, DCK, TK1, TK2, NT5C2 and CNT3) and anti‐apoptotic protein BCL‐2 in T24 and 5637 cells co‐cultured with T24‐ConMed, T24GCB‐ConMed, or 5637‐ConMed, 5637GCB‐ConMed, or T24‐EVs, T24GCB‐EVs, or 5637‐EVs, 5637GCB‐EVs for 24 h. (K, M, N) Electron microscopy images of multivesicular bodies (MVBs) and intraluminal vesicles (ILVs) in T24 and T24GCB cells. Quantification of MVBs per image (M) and ILVs per MVB (N). (L, O, P) Electron microscopy images of T24 cells incubated with T24‐EVs or T24GCB‐EVs for 24 h showing MVB and ILV structures. Quantification of MVBs per image (O) and ILVs per MVB (P). Data are presented as mean ± SEM. Statistical comparisons were performed using an unpaired two‐tailed Student's *t*‐test. *p < 0.05, **p < 0.01, ***p < 0.001. All experiments were performed independently in triplicate. MTT, 3‐(4,5‐dimethylthiazol‐2‐yl)‐2,5‐diphenyl‐tetrazolium bromide; GCB, gemcitabine; EVs, extracellular vesicles; MVBs, multivesicular bodies; ILVs, intraluminal vesicles.

Bladder cancer is characterized by frequent recurrences, which are associated with an increased risk of disease progression and necessitate more aggressive treatment strategies. Notably, multiple recurrent bladder tumours revealed similar transcriptomic profiles, suggesting the involvement of paracrine or autocrine regulation within the tumour microenvironment in post‐chemotherapy recurrence. To explore whether intercellular communication contributes to the development of GCB resistance, we treated parental T24 cells with ConMed from GCB‐resistant T24GCB cells (T24GCB ConMed). This treatment clearly induced GCB resistance in T24 cells (Figure 2A), implying the presence of transferable GCB resistance factors in the ConMed. Given the well‐established role of EVs in mediating intercellular communication within the tumour microenvironment and their documented involvement in drug resistance through the transfer of oncogenic proteins, drug efflux transporters and regulatory RNAs (Becker et al. 2016; Namee and O'Driscoll 2018), we subsequently investigated the role of EVs in modulating GCB resistance. EVs were isolated from 5637, 5637GCB, T24, T24GCB and J82 cell cultures as described in Materials and Methods. TEM confirmed the characteristic lipid bilayer morphology of EVs, with a mean diameter of approximately 100 nm (Figure 2B). NTA further validated the size distributions of EVs from all cell lines (Figure 2C). Western blotting and imaging flow cytometry confirmed the presence of key EV surface tetraspanins—CD9, CD63 and CD81—although their expression levels and distribution patterns varied among the different cell types (Figure 2D,F). To confirm the purity of isolated EVs, we examined the absence of non‐EV contaminants such as Calnexin and Cytochrome P450, following the MISEV guidelines. Western blot analysis showed that none of the EV samples (5637 EVs, 5637GCB EVs, T24 EVs, T24GCB EVs and J82 EVs) expressed Calnexin (an endoplasmic reticulum marker) and Cytochrome P450 (a mitochondrial/endoplasmic reticulum‐associated protein), supporting the specificity of EV isolation (Figure S2).

To trace the transfer of EVs from GCB‐resistant to GCB‐sensitive bladder cancer cells, EVs derived from T24GCB and 5637GCB cells were labelled with fluorescently tagged antibodies against CD9, CD63 and CD81, and then applied to T24 and 5637 recipient cells. Fluorescence microscopy and imaging flow cytometry confirmed effective EV internalization, with a higher uptake of CD63⁺ EVs by T24 and 5637 cells exposed to 5 × 10⁹ particles/mL GCB‐resistant EVs (Figures 2E and S3A–D). Following a 48 h treatment with 5 × 10⁹ particles/mL GCB‐resistant EVs, both T24 and 5637 recipient cells exhibited significantly increased GCB resistance (Figure 2G). Consistent with this, colony formation assays demonstrated enhanced clonogenic potential in cells treated with 5 × 10⁹ particles/mL GCB‐resistant EVs (Figure 2H,I). Western blot analysis revealed that both ConMed and EVs from T24GCB and 5637GCB differentially regulated key GCB‐metabolizing enzymes and transporters (Figure 2J). Specifically, ConMed derived from GCB‐resistant cells significantly upregulated CNT1 and CNT3 in recipient T24 cells, whereas EVs primarily downregulated ENT1, DCK, TK1 and TK2—key components involved in GCB uptake and activation—thereby contributing to reduced GCB efficacy. These findings suggest that once a subset of bladder cancer cells escapes from GCB‐induced cytotoxicity, the resulting GCB‐resistant cells can mediate resistance in neighbouring GCB‐sensitive cells through EV‐driven modulation. This modulation appears to reduce GCB influx and activation in recipient cells, promoting a localized microenvironment of GCB resistance.

To validate that the observed GCB resistance‐promoting effects of T24GCB EVs and 5637GCB EVs were not due to residual GCB contamination, we performed HPLC‐UV analysis to assess GCB content in these EV preparations. As positive controls, two GCB‐spiked EV samples were analysed: SP1 (J82 EVs + 0.1 µM GCB) yielded a measured concentration of 0.1025 µM and SP2 (J82 EVs + 0.05 µM GCB) yielded a concentration of 0.0437 µM. In contrast, EV samples derived from both GCB‐sensitive and GCB‐resistant bladder cancer cell lines, including SP3 (T24 EVs), SP4 (T24GCB EVs), SP5 (5637 EVs) and SP6 (5637GCB EVs), showed no detectable GCB signal (Figure S1B,C and E). These findings indicate that GCB was either completely absent or present at levels below the quantification limit in all tested EV samples. Collectively, our results confirm that EVs derived from GCB‐resistant bladder cancer cells (T24GCB and 5637GCB) do not contain residual drug, suggesting that their ability to confer GCB resistance is attributable to the intrinsic functional properties of the EVs rather than GCB carryover.

To further investigate whether EV biogenesis and secretion contribute to GCB resistance, we examined the endosomal biogenesis pathway and its associated markers. The formation of intraluminal vesicles (ILVs) within multivesicular bodies (MVBs) involves inward budding of the endosomal membrane, incorporating cargos from the cytoplasm or nucleus (Larios et al. 2020; Vietri et al. 2020). This process is tightly regulated by the endosomal sorting complex required for transport (ESCRT) machinery, which comprises ESCRT‐0, ‐I, ‐II, ‐III, VPS4‐VTA1, ALIX and TSG101 (Schöneberg et al. 2017; Kenific et al. 2021). Additionally, Rab27A and Rab27B are essential regulators of EV secretion, mediating the trafficking of MVBs to the plasma membrane and subsequent vesicle release (Ostrowski et al. 2010).

TEM revealed a greater abundance of MVBs and ILVs in T24GCB cells than in T24 cells (Figure 2K,M and N). Notably, T24 cells exposed to EVs derived from T24GCB cells exhibited an enhancement in MVBs and ILVs formation (Figure 2L, O and P), suggesting that GCB‐resistant EVs can reprogram neighbouring GCB‐sensitive cells to enhance endosomal biogenesis. This observation may reflect an increased requirement for cargo packaging and intercellular communication during the development of GCB resistance. Collectively, these findings support a model in which EVs not only transfer GCB resistance‐associated molecules but also stimulate EV biogenesis in recipient cells, thereby amplifying GCB resistance within the tumour microenvironment. The specific cargo components sorted into these EVs and their functional roles in mediating intercellular communication warrant further investigation.

### 3.3 Identification of GCB Resistance‐Specific EV Cargoes

**FIGURE 3:**
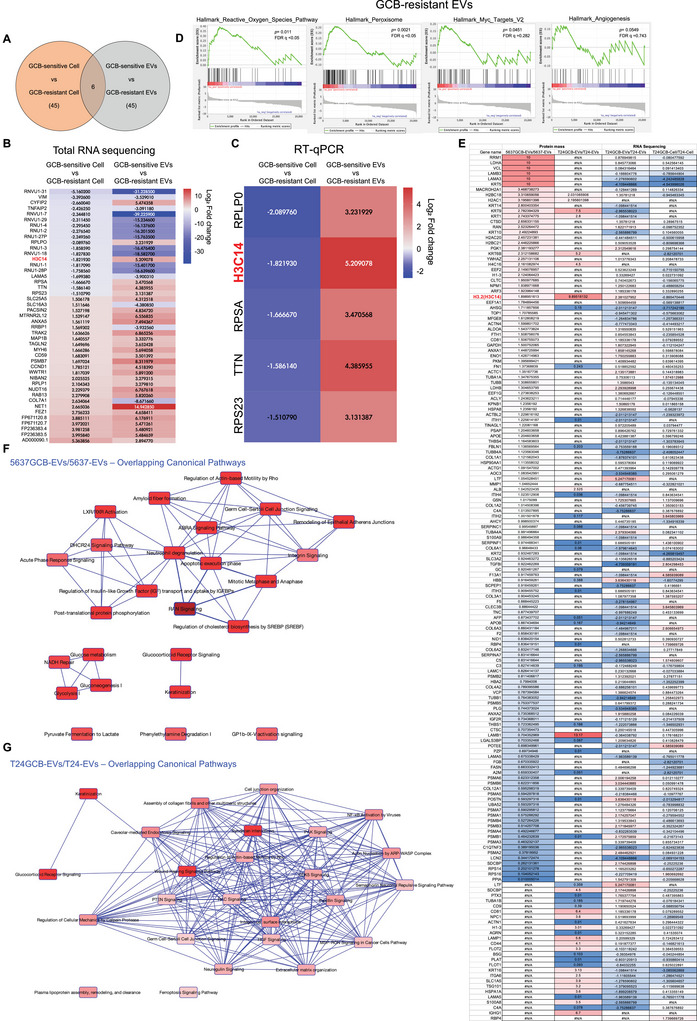
**Identification of GCB resistance‐associated genes in GCB‐resistant bladder cancer cells and their EVs by RNA sequencing**. (A) Schematic overview of total RNA sequencing comparing GCB‐sensitive bladder cancer cells (T24 and 5637) with their GCB‐resistant counterparts (T24GCB and 5637GCB), as well as EVs derived from GCB‐sensitive (T24‐EVs and 5637‐EVs) and GCB‐resistant cells (T24GCB‐EVs and 5637GCB‐EVs). (B) The heatmap illustrates DEGs between GCB‐sensitive and GCB‐resistant cells and EVs. DEGs were identified using a fold change > 2 (Log_2_FC > 1.5 or <–1.5) and adjusted *p* < 0.01. The colour scale represents relative gene expression levels (blue = low, red = high). (C) RT‐qPCR validation of selected DEGs in GCB‐sensitive and GCB‐resistant bladder cancer cells, as well as their corresponding EVs. (D) Pathway enrichment analysis of DEGs identified in GCB‐resistant EVs identified several significantly enriched pathways, including the ROS pathway, peroxisome, Myc targets V2 and angiogenesis. (E) Comparative heatmap of proteomic profiling illustrates differential protein expression in GCB‐resistant versus GCB‐sensitive EVs. The analysis identified overlapping candidates with RNA‐seq data from both cell (T24GCB vs. T24) and EV (T24GCB‐EVs vs. T24‐EVs) datasets. (F and G) Canonical pathway analysis using IPA identified overlapping enriched pathways between GCB‐resistant and GCB‐sensitive EVs in (F) 5637GCB‐EVs versus 5637‐EVs and (G) T24GCB‐EVs versus T24‐EVs. GCB, gemcitabine; EVs, extracellular vesicles; DEGs, differentially expressed genes; RT‐qPCR, reverse transcription quantitative polymerase chain reaction; ROS, reactive oxygen species; IPA, Ingenuity Pathway Analysis.

Given the role of EVs in mediating GCB resistance, we next sought to identify key molecular contributors through transcriptomic profiling. RNA sequencing (RNA‐seq) was performed to compare GCB‐sensitive bladder cancer cells (T24 and 5637) with their GCB‐resistant counterparts (T24GCB and 5637GCB). This analysis revealed 45 significantly differentially expressed genes (adjusted *p* < 0.0001), many of which were correlated with ribosomal function and chromatin regulation (Figure 3A,B). RNA‐seq profiles of EVs derived from GCB‐sensitive and GCB‐resistant cells were subsequently compared. Notably, five genes—RPLP0, H3C14, RPSA, TTN and RPS23—were downregulated in GCB‐resistant cells but enriched in EVs derived from the same cells (Figure 3C), suggesting a selective cargo sorting mechanism.

Simultaneously, proteomic profiling was performed on EVs from GCB‐resistant and GCB‐sensitive cells to generate comparative heatmaps. Pathway enrichment analysis of DEGs identified in GCB‐resistant EVs identified several significantly enriched pathways, including the ROS pathway, peroxisome, Myc targets V2 and angiogenesis (Figure 3D). Integrative analysis combining EV proteomics (5637GCB‐EVs vs. 5637‐EVs and T24GCB‐EVs vs. T24‐EVs) with RNA‐seq data from both cellular and EV samples identified eight overlapping genes: H2BC18, H2AC1, KRT9, KRT6B, H4C16, H3.2 (H3C14), AL and LAMB1. Among these, only H3.2 (H3C14) was consistently and significantly downregulated in GCB‐resistant cells but upregulated in their corresponding EVs at the mRNA and protein levels (Figure 3E). This pattern suggests that a selective EV‐based mechanism may contribute to the depletion of H3.2 within GCB‐resistant cells, potentially representing a novel pathway of GCB resistance. To further explore the underlying mechanisms, we conducted canonical pathway analysis using Ingenuity Pathway Analysis on the differentially expressed mRNAs from 5637GCB‐EVs and T24GCB‐EVs. This analysis revealed several commonly enriched pathways across both GCB‐resistant EV datasets, including neutrophil degranulation, germ cell–Sertoli cell junction signalling, regulation of actin‐based motility by Rho, wound healing signalling, phosphatase and tensin homolog signalling, keratinization, p21‐activated kinase signalling, NF‐κB activation by viruses and extracellular matrix organization (Figure 3F,G). Many of these pathways are strongly associated with tumour progression, immune modulation and chemoresistance, suggesting that EV cargoes derived from GCB‐resistant cells actively contribute to the establishment of a tumour‐promoting and drug‐resistant microenvironment.

### 3.4 H3C14 as a Key Regulator of GCB Resistance in Bladder Cancer

**FIGURE 4:**
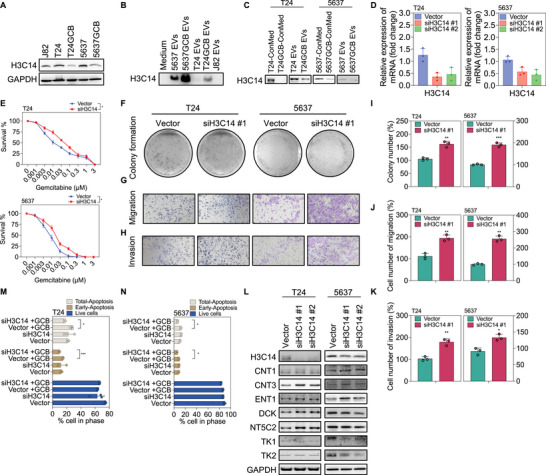
**Functional characterization of H3C14 in GCB resistance in bladder cancer cells**. (A) Western blot analysis of histone H3.2 (H3C14) protein expression in GCB‐sensitive bladder cancer cells (T24 and 5637) versus their GCB‐resistant counterparts (T24GCB and 5637GCB) and J82 cells. (B) Western blot analysis of histone H3.2 (H3C14) protein levels in EVs isolated from 5637, 5637GCB, T24, T24GCB and J82 cells. (C) Western blot analysis of histone H3.2 (H3C14) in T24 and 5637 cells after 24 h treatment with conditioned media (ConMed) or EVs derived from T24, T24GCB and 5637, 5637GCB cells. (D) RT‐qPCR analysis was used to evaluate the knockdown efficiency of H3C14 RNA in T24 and 5637 cells transfected with H3C14 siRNA (siH3C14#1 and siH3C14#2). (E) MTT assay was used to evaluate cell viability in T24 and 5637 cells transfected with scramble siRNA (Vector) or siH3C14 following treatment with a series of GCB concentrations (0–3 µM) for 48 h (*n* = 6 per group). (F and I) Colony formation assays were used to evaluate measuring clonogenic survival clonogenicity of T24 and 5637 cells transfected with Vector or siH3C14 over 14 days (*n* = 3 per group). (G and J) Migration assays were used to evaluate the migratory capacity of T24 and 5637 cells transfected with Vector or siH3C14 (*n* = 3 per group). (H and K) Invasion assays were used to evaluate the invasive ability of T24 and 5637 cells transfected with Vector or siH3C14 (*n* = 3 per group). (M and N) Flow cytometry analysis of apoptosis in T24 and 5637 cells transfected with Vector or siH3C14 following treatment with 0.01 µM gemcitabine. Apoptosis was assessed using PI and Annexin V staining (*n* = 3 per group). (L) Western blot analysis of histone H3.2 (H3C14) and GCB‐metabolizing enzymes and transporters (CNT1, ENT1, DCK, TK1, TK2, NT5C2 and CNT3) in T24 and 5637 cells transfected with Vector, siH3C14#1, or siH3C14#2. For all panels, data are presented as mean ± SEM. An unpaired two‐tailed Student's *t*‐test was used for comparisons between groups. *p < 0.05, **p < 0.01 and ***p < 0.001. All experiments were independently repeated at least three times. GCB, gemcitabine; EVs, extracellular vesicles; RT‐qPCR, reverse transcription quantitative polymerase chain reaction; MTT, 3‐(4,5‐dimethylthiazol‐2‐yl)‐2,5‐diphenyl‐tetrazolium bromide; PI, propidium iodide.

Histones are small (15–21 kDa), highly conserved, positively charged proteins serving as the core structural components of chromatin. The fundamental unit of chromatin, the nucleosome, consists of approximately 146 base pairs of superhelical DNA wrapped around a histone octamer, which consists of the core histones H2A, H2B, H3 and H4, along with the linker histone H1 (Mariño‐Ramírez et al. 2005). Nucleosomes are highly vulnerable to DNA damage and strand breaks induced by chemotherapeutic agents, ultraviolet radiation, or ionizing radiation. Such insults can lead to the release of nucleosome fragments and extranuclear histones. Based on their localization, these extranuclear histones can be categorized as free cytoplasmic histones, circulating histones in systemic circulation, or vesicle‐associated histones (Singh et al. 2022; Jeppesen et al. 2019). H3C14 (H3 Clustered Histone 14; HIST2H3C) is a member of the histone H3.2 gene family, which also includes H3C13 and H3C15. All three genes encode the identical histone H3.2 protein. Although histone H3 serves as a core nucleosomal component, its variants—including H3.1, H3.2, H3.3 and centromeric H3 (CEN‐H3)—exhibit distinct roles in chromatin organization and gene regulation (Kamakaka and Biggins 2005; Gurard‐Levin et al. 2014). Notably, H3C14 mRNA and protein were highly enriched in GCB‐resistant EVs, as confirmed via RT‐qPCR (Figure 3C) and western blot analysis (Figure 4A,B). To investigate how H3C14 expression is modulated under GCB‐resistant conditions, we performed western blot analysis of T24 and 5637 cells treated with either ConMed or T24GCB‐derived EVs. Both treatments resulted in a significant reduction in intracellular H3C14 expression (Figure 4C), suggesting that GCB‐resistant cells can suppress H3C14 in neighbouring cells through secreted factors or EV‐mediated transfer. To explore the functional role of H3C14 in GCB resistance, we conducted loss‐of‐function experiments using siRNA‐mediated knockdown. T24 and 5637 bladder cancer cells were transfected with two independent siRNAs targeting H3C14 (siH3C14#1 and siH3C14#2), and knockdown efficiency was confirmed using RT‐qPCR (Figure 4D). Among the two, siH3C14#1 was selected for subsequent experiments based on superior knockdown performance. MTT assays demonstrated that H3C14 knockdown significantly increased GCB resistance (0.01 µM) in both T24 and 5637 cells, making them more resistant to GCB than vector controls (Figure 4E).

To further assess the effect of H3C14 knockdown on malignant properties, colony formation, migration and invasion assays were conducted (Figure 4F–K). The results demonstrated that H3C14 knockdown significantly enhanced cell clonogenicity, motility and invasiveness. Flow cytometric analysis of apoptosis, using propidium iodide and Annexin V staining, revealed that siH3C14‐transfected cells exhibited a greater reduction in apoptosis under GCB treatment (0.01 µM) than that observed in controls (Figure 4M,N). Next, we investigated how H3C14 modulates GCB‐metabolizing enzymes and transporters. Western blot analysis demonstrated that H3C14 knockdown increased the expression of CNT3 and NT5C2 while significantly downregulating TK1 levels (Figure 4L). Collectively, these findings identify H3C14 as a critical modulator of GCB sensitivity and tumour aggressiveness in bladder cancer. Loss of H3C14 enhances GCB resistance, promotes malignant phenotypes, reduces apoptosis and alters the expression of key GCB‐metabolizing enzymes, highlighting its potential as a therapeutic target in GCB‐refractory bladder cancer.

**FIGURE 5:**
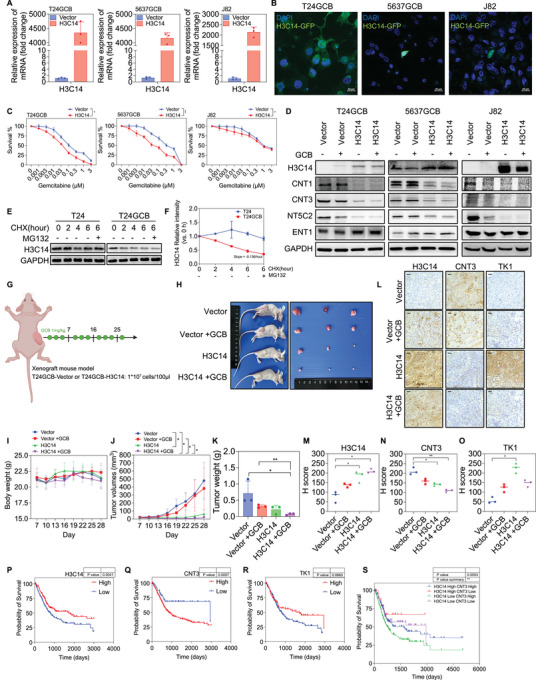
**Functional characterization of H3C14 gain‐of‐function in GCB‐resistant bladder cancer cells and its role in GCB sensitivity**. (A) RT‐qPCR analysis was conducted to confirm H3C14 overexpression in T24GCB, 5637GCB and J82 cells transfected with H3C14‐GFP plasmid. (B) Representative fluorescence microscopy images show subcellular localization of H3C14‐GFP in T24GCB, 5637GCB, and J82 cells. Scale bar: 20 µm. (C) MTT assays were used to evaluate cell viability in T24GCB‐H3C14‐GFP, 5637GCB‐H3C14‐GFP and J82‐H3C14‐GFP cells after 48 h treatment with GCB concentrations ranging from 0 to 3 µM (*n* = 6 per group). (D) Western blot analysis of histone H3.2 (H3C14) and GCB‐metabolizing enzymes and transporters (CNT1, ENT1, NT5C2 and CNT3) in T24GCB, 5637GCB and J82 cells transfected with either vector or H3C14‐GFP, with or without 0.01 µM GCB treatment. (E and F) Western blot analysis of histone H3.2 (H3C14) protein stability in T24 and T24GCB cells treated with cycloheximide (CHX, 50 µg/mL) for 0, 2, 4 or 6 h, and with MG132 (10 µM) for 6 h. (G) Schematic diagram of xenograft tumour model in nude mice—T24GCB‐Vector or T24GCB‐H3C14 cells were subcutaneously injected, followed by GCB treatment (1 mg/kg every 2 days for 28 days). (H) Representative tumour images from the four treatment groups: Vector, Vector + GCB, H3C14 and H3C14 + GCB (*n* = 3 mice per group). (I) Tumour weight comparison among the four groups (*n* = 3 mice per group). (J) Tumour growth curves present changes in tumour volume over time across treatment groups (*n* = 3 mice per group). (K) Histogram of final tumour weights from each treatment group (*n* = 3 mice per group). (L–O) IHC staining of H3C14, CNT3 and TK1 in xenograft tumours from the four groups. H‐scores were calculated to quantify expression levels (*n* = 3 mice per group). (P–R) Kaplan–Meier survival analysis of patients with bladder cancer from TCGA datasets based on high versus low expression of H3C14, CNT3 and TK1. (S) TCGA survival analysis based on high H3C14 and low CNT3 expression revealed the highest survival probability in this subgroup. Data are presented as mean ± SEM. Statistical comparisons were made using an unpaired two‐tailed Student's *t*‐test. *p < 0.05, **p < 0.01, ***p < 0.001. All experiments were independently repeated at least three times. GCB, gemcitabine; RT‐qPCR, reverse transcription quantitative polymerase chain reaction; MTT, 3‐(4,5‐dimethylthiazol‐2‐yl)‐2,5‐diphenyl‐tetrazolium bromide; IHC, Immunohistochemical; TCGA, The Cancer Genome Atlas.

In contrast, gain‐of‐function assays were conducted to further investigate the roles of H3C14 in GCB resistance. H3C14 was overexpressed in T24GCB, 5637GCB and J82 cells through transfection with an H3C14‐GFP plasmid. RT‐qPCR confirmed a significant upregulation of H3C14 mRNA (Figure 5A), and fluorescence microscopy revealed the localization of histone H3.2 (H3C14) protein in the cytoplasm and nucleus (Figure 5B). MTT assays demonstrated that H3C14 overexpression restored GCB sensitivity in GCB‐resistant cells (Figure 5C). Western blot analysis further revealed that H3C14 overexpression resulted in a reduction of CNT1, CNT3 and NT5C2 levels, while upregulating ENT1 (Figure 5D). Since T24 and T24GCB cell lines demonstrated better responses or effects compared with 5637 and J82 in those cellular and molecular biology assays, they were used to the time‐course analysis of H3.2 (H3C14) protein stability and for the generation of mice xenografts. To explore whether the reduction in intracellular H3.2 (H3C14) protein levels resulted from accelerated protein degradation, a time‐course analysis of protein stability was conducted in T24 and T24GCB cells treated with cycloheximide, a selective inhibitor of protein translation, or MG132, a 26S proteasome inhibitor. T24GCB cells exhibited a more pronounced reduction in H3.2 (H3C14) protein levels over time than did T24 cells, and this decline remained unaffected by MG‐132 treatment (Figure 5E,F). These findings suggest that the protein stability of H3.2 (H3C14) is not altered following the acquisition of GCB resistance; however, EV‐mediated H3.2 (H3C14) excretion likely plays a critical role in GCB resistance.

To assess the role of H3C14 in vivo, we established a subcutaneous xenograft tumour model using T24GCB‐Vector and T24GCB‐H3C14 cells in nude mice (Figure 5G). Tumour‐bearing mice treated with GCB developed significantly smaller tumours in the H3C14‐overexpressing group than those of controls (Figure 5H–K). Immunohistochemical analysis confirmed increased nuclear expression of H3.2 (H3C14), decreased CNT3 levels and increased TK1 levels in H3C14‐overexpressing tumours (Figure 5L–O). Analysis of the Cancer Genome Atlas–Bladder Urothelial Carcinoma dataset revealed that patients with bladder cancer with high H3C14 expression had significantly better overall survival (*p* = 0.0041); meanwhile, higher CNT3 expression was associated with poorer outcomes (*p* = 0.0057), and higher TK1 expression demonstrated a trend toward improved outcomes (*p* = 0.0665) (Figure 5P–R). Furthermore, combined expression analysis demonstrated that patients with high H3C14 and low CNT3 expression had the most favourable prognosis (*p* = 0.0053) (Figure 5S).

### 3.5 Process of EV Secretion Involved in H3.2 (H3C14) Excretion in GCB‐Resistant Bladder Cancer Cells

**FIGURE 6:**
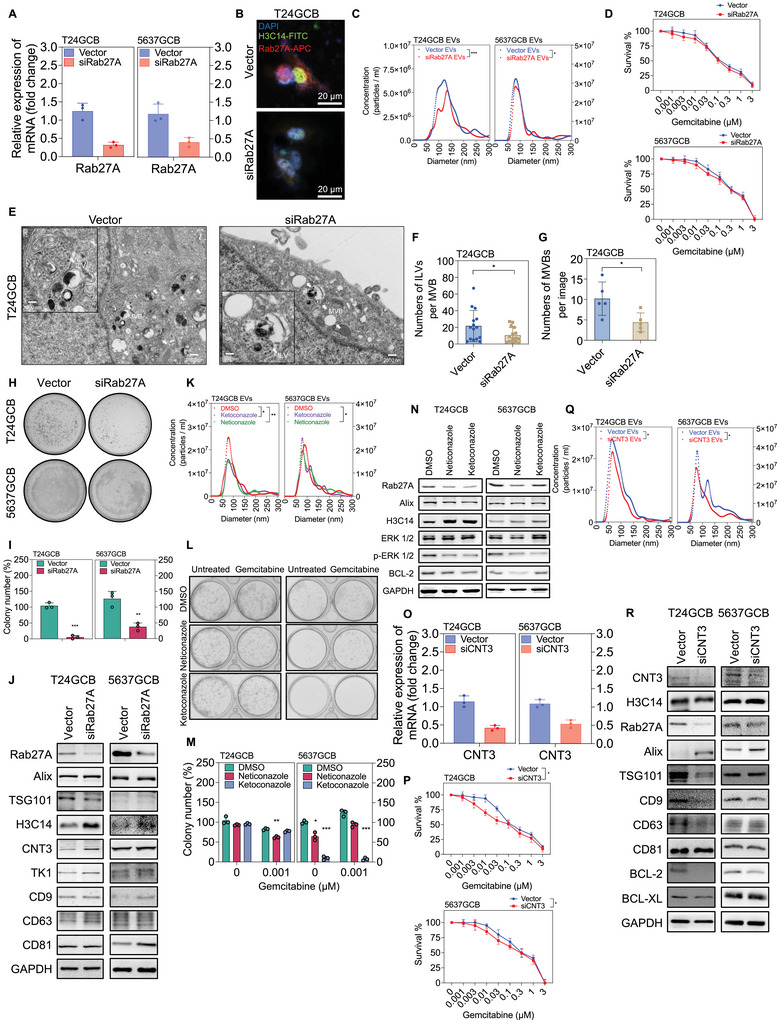
**Rab27A regulates EV release and histone H3.2 (H3C14) protein excretion in GCB‐resistant bladder cancer cells**. (A) RT‐qPCR analysis was used to evaluate Rab27A knockdown efficiency in T24GCB and 5637GCB cells following siRab27A transfection. (B) Representative fluorescence microscopy images of T24GCB‐Vector and T24GCB‐siRab27A cells stained with H3C14‐FITC, Rab27A‐APC and DAPI. Scale bar: 20 µm. (C) Nanoparticle tracking analysis (NS300) of EVs released from T24GCB‐Vector, T24GCB‐siRab27A and 5637GCB‐Vector, 5637GCB‐siRab27A cells revealed particle size distribution and concentration. (D) MTT assay was used to evaluate cell viability of T24GCB‐Vector, T24GCB‐siRab27A and 5637GCB‐Vector, 5637GCB‐siRab27A cells after 48 h treatment with GCB concentrations ranging from 0 to 3 µM (*n* = 6 per group). (E) Representative electron microscopy images show MVBs and ILVs in T24GCB‐Vector, T24GCB‐siRab27A and 5637‐Vector, 5637GCB‐siRab27A cells. (F, G) Quantification of MVBs per image (F) and ILVs per MVB (G) from panel L. (H, I) Colony formation assays were used to evaluate clonogenicity of T24GCB and 5637GCB cells transfected with Vector or siRab27A over 10 days (*n* = 3 per group). (J) Western blot analysis of Rab27A, H3C14, CNT3 and TK1, as well as EV‐associated proteins (CD9, CD63, CD81, TSG101 and Alix) in T24GCB and 5637GCB cells transfected with Vector or siRab27A. (K) Nanoparticle tracking analysis (NS300) of EVs derived from T24GCB and 5637GCB cells treated with DMSO, neticonazole (1 µM), or ketoconazole (1 µM) for 24 h. (L, M) Colony formation assays in T24GCB and 5637GCB cells treated with DMSO, neticonazole, or ketoconazole (1 µM each) ± GCB (0.001 µM) for 7 days (*n* = 3 per group). (N) Western blot analysis of Rab27A, H3C14, Alix, p‐ERK1/2, total ERK1/2 and BCL‐2 in T24GCB and 5637GCB cells after 24 h treatment with DMSO, neticonazole, or ketoconazole. (O) RT‐qPCR analysis was used to evaluate CNT3 knockdown efficiency in T24GCB and 5637GCB cells after siCNT3 transfection. (P) Nanoparticle tracking analysis (NS300) of EVs derived from T24GCB‐Vector, T24GCB‐siCNT3, and 5637GCB‐Vector, 5637GCB‐siCNT3 cells. (Q) MTT assay was used to evaluate viability of T24GCB‐Vector, T24GCB‐siCNT3 and 5637GCB‐Vector, 5637GCB‐siCNT3 cells treated with GCB (0–3 µM) for 48 h (*n* = 6 per group). (R) Western blot analysis of CNT3, H3C14, Rab27A, EV markers (CD9, CD63, CD81, Alix and TSG101) and anti‐apoptotic proteins (BCL‐2 and BCL‐XL) in T24GCB and 5637GCB cells transfected with Vector or siCNT3. All data are presented as mean ± SEM. Statistical comparisons were performed using an unpaired two‐tailed Student's *t*‐test. *p < 0.05, **p < 0.01, ***p < 0.001. All experiments were repeated independently at least three times. GCB, gemcitabine; EVs, extracellular vesicles; RT‐qPCR, reverse transcription quantitative polymerase chain reaction; MTT, 3‐(4,5‐dimethylthiazol‐2‐yl)‐2,5‐diphenyl‐tetrazolium bromide; MVBs, multivesicular bodies; ILVs, intraluminal vesicles.

Rab27A and Rab27B, members of the Rab GTPase family, are key regulators of EV secretion (Ostrowski et al. 2010). Rab27A‐mediated EV release has been implicated in several oncogenic processes, including: (1) activation of epithelial‐to‐mesenchymal transition in highly metastatic hepatocellular carcinoma cells (Chen et al. 2018); (2) activation of NF‐κB signalling, contributing to chemoresistance in bladder cancer (Liu et al. 2017) and (3) induction of Nanog expression and regorafenib resistance in cancer stem cells (Huang et al. 2021). Rab27A specifically regulates EV release during the late endosomal stage, thereby affecting drug resistance and tumour progression. Given that EV‐mediated H3.2 (H3C14) excretion is involved in GCB resistance in bladder cancer cells, inhibition of EV biogenesis and secretion through Rab27A knockdown using siRab27A in T24GCB cells was performed. Following transfection with siRab27A for 48 h, RT‐qPCR analysis revealed a significant reduction in Rab27A mRNA expression (Figure 6A). Immunofluorescence staining demonstrated a significant reduction in Rab27A protein expression, while H3.2 (H3C14) protein levels increased in the nucleus (Figure 6B).

Moreover, nanoparticle tracking analysis revealed a greater reduction in EV concentration in the siRab27A group than in the control (Figure 6C). Despite this reduction, MTT assays showed no significant difference in GCB‐induced cytotoxicity between the siRab27A and scramble siRNA groups (Figure 6D), suggesting that Rab27A knockdown does not directly affect GCB sensitivity. Similarly, TEM demonstrated a significant decrease in the formation of MVBs and ILVs in the siRab27A group (Figure 6E–G). However, colony formation assays revealed a significant decrease in clonogenicity following Rab27A knockdown (Figure 6H,I), implicating an inhibitory effect on tumorigenicity. Western blot analysis demonstrated that siRab27A significantly upregulated H3.2 (H3C14) protein levels, with a mild increase in CNT3 expression (Figure 6J). Collectively, these findings suggest that Rab27A knockdown impairs EV secretion, modulates histone H3.2 (H3C14) protein levels and suppresses clonogenicity, while exerting a limited impact on GCB metabolism. Moreover, these results may help explain the tumorigenic and tumour‐progressive roles of Rab27A in previous studies through EV‐associated mechanisms and extranuclear histone dynamics, particularly by modulating histone H3.2 (H3C14). To further verify whether H3C14 interacts with Rab27A or CNT3, co‐immunoprecipitation assays were performed using T24GCB cells and T24GCB cells treated with 0.1 µM GCB. The results demonstrated that histone H3.2 (H3C14) can bind to both Rab27A and CNT3. The H3.2 (H3C14)–Rab27A interaction may suggest a role for H3.2 (H3C14) in the formation or secretion of EVs, whereas the H3.2 (H3C14)–CNT3 interaction may imply that H3.2 (H3C14) potentially regulates or suppresses CNT3 expression (Figure S4).

Additionally, small compounds inhibiting EV secretion were introduced to evaluate the role of EV‐mediated GCB resistance in bladder cancer cells. Previous research by Datta et al. (Datta et al. 2018) has suggested that imidazole derivatives, such as neticonazole, ketoconazole and climbazole, inhibit Rab27A‐associated EV protein fusion and reduce EV secretion, although the underlying molecular mechanisms remain largely undefined. Notably, neticonazole has been demonstrated to suppress EV release, improve intestinal dysbiosis, and attenuate colorectal tumorigenesis (Gu et al. 2020). To assess the impact of EV secretion inhibitors on GCB resistance, T24GCB and 5637GCB cells were treated with 1 µM ketoconazole or neticonazole for 24 h. NTA confirmed a significant reduction in EV concentration in both treatment groups (Figure 6K). Furthermore, colony formation assays demonstrated a significant decrease in clonogenicity, with neticonazole exerting a more potent suppressive effect than that of ketoconazole (Figure 6L,M). Western blot analysis revealed that both ketoconazole and neticonazole treatments increased intracellular H3.2 (H3C14) protein levels while simultaneously downregulating Rab27A, phosphorylated ERK1/2 (p‐ERK1/2), and the anti‐apoptotic protein BCL‐2 (Figure 6N), suggesting that imidazole‐mediated suppression of Rab27A may indirectly restore intracellular H3.2 (H3C14) protein levels and suppress pro‐survival signalling. To evaluate the spatial correlation between Rab27A and H3.2 (H3C14) proteins under GCB treatment, imaging flow cytometry was used to assess their intracellular protein localization in T24, T24GCB and J82 cells. After a 24 h exposure to GCB (0.1 µM), T24 cells exhibited a marked increase in Rab27A expression, accompanied by a substantial reduction in nuclear H3.2 (H3C14) protein. In contrast, the GCB‐resistant T24GCB and J82 cells consistently exhibited higher Rab27A expression, regardless of GCB treatment. Notably, Rab27A was observed to translocate into the nucleus and co‐localize with H3.2 (H3C14) protein in these GCB‐resistant cells, suggesting a functional interaction between Rab27A and H3.2 (H3C14) that may contribute to chemoresistance (Figure S5A,B).

### 3.6 Inhibition of CNT3 Attenuates EV‐Mediated GCB Resistance in Bladder Cancer

Among the screened GCB‐metabolizing enzymes and transporters in this study, CNT3 was the most dramatically increased protein in GCB‐resistant cells compared to that in GCB‐sensitive cells (Figure 1I), suggesting that CNT3 interacts with H3.2 (H3C14) to induce and promote EV‐mediated GCB resistance. To evaluate the role of CNT3 in GCB metabolism and EV biogenesis, CNT3 expression was knocked down in T24GCB and 5637GCB cells using siRNA (siCNT3), with successful knockdown confirmed via RT‐qPCR (Figure 6O). ConMed was collected 24 h post‐transfection, and NTA revealed a greater decrease in EV concentration in the siCNT3 group than in the scramble siRNA control (Figure 6P). Consistently, MTT assays demonstrated that CNT3 knockdown significantly enhanced GCB sensitivity in T24GCB and 5637GCB cells (Figure 6Q), suggesting that CNT3 contributes to GCB resistance. Western blot analysis further revealed that siCNT3 reduced the expression of Rab27A, TSG101, CD9, CD63 and the anti‐apoptotic protein BCL‐2, while upregulating Alix, a component involved in EV biogenesis. However, H3.2 (H3C14) protein levels remained unchanged (Figure 6R), indicating that CNT3 does not directly regulate H3C14 expression but may affect GCB resistance by regulating the EV secretory process. Collectively, these findings highlight CNT3 as a crucial regulator of EV secretion and GCB resistance.

### 3.7 Characterization of Histone H3.2 (H3C14)‐Carrying EVs in GCB Resistance

**FIGURE 7:**
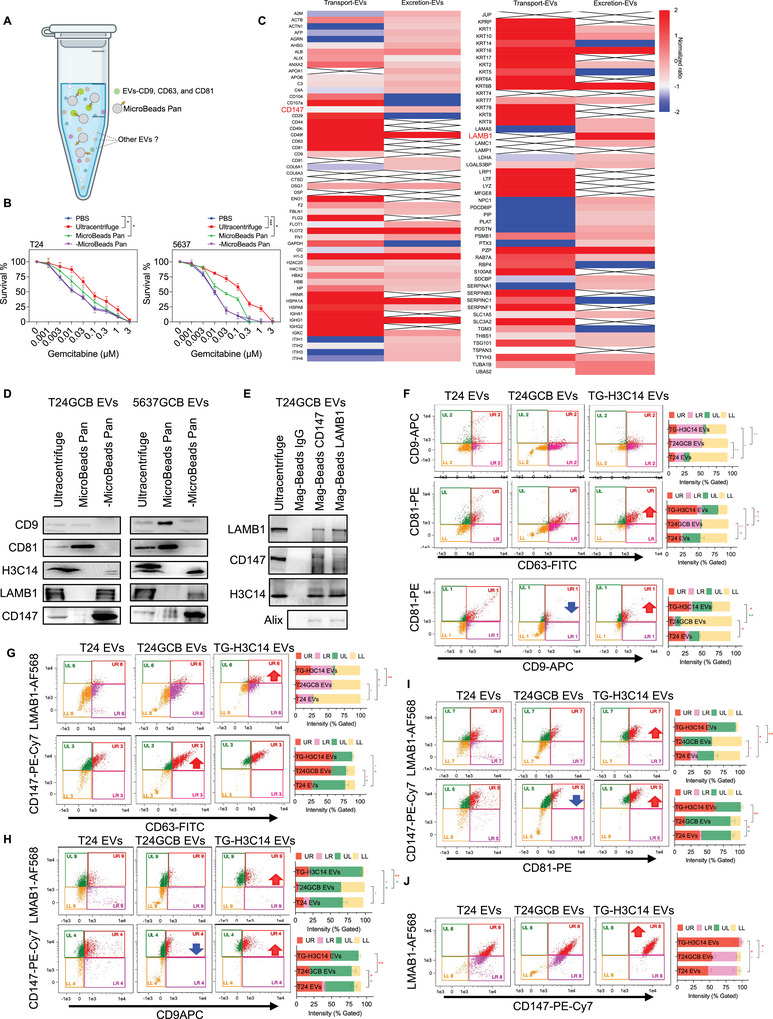
**Characterization of H3.2 (H3C14)‐carrying EVs in GCB Resistance**. (A) Schematic illustration of the isolation of EV subpopulations from T24GCB cells using MicroBeads Pan, targeting CD9, CD63 and CD81. (B) MTT assay was used to assess the viability of T24 and 5637 cells treated with PBS, ultracentrifugation‐derived T24GCB‐EVs (or 5637GCB‐EVs), or MicroBeads Pan‐isolated EVs (+MicroBeads Pan), or EVs not captured by MicroBeads Pan (–MicroBeads Pan). Cells were subsequently treated with a series of GCB concentrations (0–3 µM) for 48 h (*n* = 6 per group). (C) Mass spectrometry‐based comparative proteomic profiling of Transport‐EVs and Excretion‐EVs. The heatmap illustrates differentially expressed proteins, with red indicating higher expression and blue indicating lower expression levels. (D) Western blot analysis of EV‐associated markers (CD9 and CD81), histone H3.2 (H3C14), LAMB1 and CD147 in EVs derived from ultracentrifugation, MicroBeads Pan‐enriched (+MicroBeads Pan), or non‐captured (–MicroBeads Pan) fractions from T24GCB‐EVs and 5637GCB‐EVs. EVs may be broadly classified into Transport‐EVs (either CD9+, CD63+, or CD81+) and Excretion‐EVs (lacking CD9, CD63 and CD81). (E) Western blot analysis of histone H3.2 (H3C14), Alix, LAMB1 and CD147 in EVs isolated via ultracentrifugation or immunoprecipitation using magnetic beads conjugated with IgG, anti‐CD147, or anti‐LAMB1 antibodies from T24GCB‐EVs. (F–I) Imaging flow cytometry analysis of EVs derived from T24‐EVs, T24GCB‐EVs and T24GCB‐H3C14‐EVs (TG‐H3C14 EVs). EVs were stained with a lipid bilayer dye and labelled with CD9‐APC, CD63‐FITC, CD81‐PE, LAMB1‐AF568 and CD147‐Cy7 antibodies. Percentage gated values represent the proportion of EVs co‐expressing the indicated surface markers: (F) CD63⁺CD9⁺, CD63⁺CD81⁺, CD63⁺LAMB1⁺ and CD63⁺CD147⁺ (G) CD9⁺CD81⁺, CD9⁺LAMB1⁺ and CD9⁺CD147⁺ (H) CD81⁺LAMB1⁺ and CD81⁺CD147⁺ (I) CD147⁺LAMB1⁺ For all panels, data are presented as mean ± SEM. Statistical significance was determined using an unpaired two‐tailed Student's *t*‐test. *p < 0.05, **p < 0.01, ***p < 0.001. All experiments were repeated independently at least three times. GCB, gemcitabine; EVs, extracellular vesicles; RT‐qPCR, reverse transcription quantitative polymerase chain reaction; MTT, 3‐(4,5‐dimethylthiazol‐2‐yl)‐2,5‐diphenyl‐tetrazolium bromide; PBS, paraformaldehyde.

The size, morphology and surface markers of isolated EVs revealed exosome‐like properties. To more specifically fractionate EV subpopulations, we utilized MicroBeads Pan targeting CD9, CD63 and CD81. Based on surface marker distribution, EVs were broadly classified into two distinct groups: (1) CD9^−^/CD63^−^/CD81^−^ EVs—lacking CD9, CD63 and CD81, and (2) non‐CD9^−^/CD63^−^/CD81^−^ EVs—positive for at least one of CD9, CD63 and CD81 (Figure 7A). To assess the functional impact of these subpopulations, T24 and 5637 recipient cells were treated with either non‐CD9^−^/CD63^−^/CD81^−^ EVs (MicroBeads Pan‐captured) or CD9^−^/CD63^−^/CD81^−^ EVs (non‐captured) for 72 h (Figure 7B). MTT assays demonstrated that non‐CD9^−^/CD63^−^/CD81^−^ EVs significantly enhanced GCB resistance in T24 and 5637 cells, whereas CD9^−^/CD63^−^/CD81^−^ EVs failed to induce any GCB‐resistant phenotype. These results suggest that non‐CD9^−^/CD63^−^/CD81^−^ EVs may be classified as “Transport‐EVs,” transferring resistance‐related molecules to neighbouring sensitive cells, whereas the CD9^−^/CD63^−^/CD81^−^ EVs may be designated as “Excretion‐EVs,” acting as scavengers for unwanted or potentially immunogenic cargoes. These findings underscore the importance of different EV subtypes in enhancing chemoresistance in bladder cancer, with Transport‐EVs potentially facilitating the transfer of resistance‐related molecules to neighbouring sensitive cells, and excretion‐EVs acting as scavengers for unwanted or potentially immunogenic cargoes.

Given the unclear role of excretion‐EVs in GCB resistance, we performed proteomic profiling using mass spectrometry to compare the protein contents of transport‐EVs and excretion‐EVs (Figure 7C). To further elucidate the functional distinctions between these EV subpopulations, we performed GO enrichment analysis using Ingenuity Pathway Analysis based on the proteomic data. GO biological process analysis of Transport‐EVs revealed enrichment in pathways related to cytoskeletal remodelling and epithelial barrier integrity, including intermediate filament organization, keratinization, establishment of skin barrier and keratinocyte differentiation, as well as immune‐related processes, such as cell adhesion mediated by integrin and the humoral immune response (Figure S6A). Furthermore, GO cellular component analysis highlighted associations with structures, such as the integrin complex, keratin filament, cornified envelope and blood microparticles, suggesting that Transport‐EVs may play a role in both structural reorganization and immune modulation (Figure S6B). In contrast, Excretion‐EVs were enriched in distinct functional categories. GO molecular function analysis indicated involvement in structural molecule activity, exogenous protein binding, virus receptor activity and cell adhesion molecule binding (Figure S6C). Their GO cellular component profile further revealed associations with extracellular membrane‐bounded organelles, keratin filaments and the EV space, reinforcing their distinct localization and cargo content compared to those of Transport‐EVs (Figure S6D). Together, these findings suggest that Transport‐EVs actively contribute to cancer cell–cell communication and promote GCB resistance, whereas Excretion‐EVs may play a more passive role by offloading intracellular components, such as H3.2 (H3C14), thereby modulating the tumour microenvironment and exacerbating GCB resistance. Notably, H3.2 (H3C14) protein was specifically present in CD9^−^/CD63^−^/CD81^−^ EVs, as confirmed through western blot analysis (Figure 7D).

To identify potential surface markers for Excretion‐EVs, the cluster of differentiation dataset from the Human Genome Organization Gene Nomenclature Committee (https://www.genenames.org/data/genegroup/#!/group/471) was used to compare the mass spectrometry data of Transport‐EVs and Excretion‐EVs. After screening, LAMB1 and CD147 were significantly enriched in Excretion‐EVs. Further validation using western blot analysis revealed higher levels of CD9 and CD81 in Transport‐EVs, whereas H3.2 (H3C14), LAMB1 and CD147 were predominantly detected in Excretion‐EVs (Figure 7D). To confirm whether H3.2 (H3C14) protein is transported by specific subtypes of Excretion‐EVs, we conducted immunoprecipitation using magnetic beads conjugated with anti‐CD147 or anti‐LAMB1 antibodies. Western blot analysis of the immunoprecipitated EVs revealed co‐detection of H3.2 (H3C14) protein along with LAMB1 and CD147, indicating that both CD147^+^ EVs and LAMB1^+^ EVs can transport H3.2 (H3C14) protein (Figure 7E). To validate that Excretion‐EVs, characterized by low or undetectable levels of canonical tetraspanins, are EVs, we performed FM 1–43 membrane dye staining and imaging‐based analysis. EVs were captured using magnetic beads coated with antibodies targeting either CD9⁺CD63⁺CD81⁺ (Transport‐EVs) or CD147⁺LAMB1⁺ (Excretion‐EVs) and analysed using the ImageStreamX imaging flow cytometry system. Both populations exhibited FM 1–43 positivity, supporting their membrane vesicular identity (Figure S7A,B). Additionally, SEM revealed spherical vesicular structures on the antibody‐coated beads, confirming the physical presence of EVs (Figure 7C). These findings suggest that H3.2 (H3C14) protein may be selectively sorted into Excretion‐EVs through association with these membrane markers. Although Excretion‐EVs did not directly induce drug resistance in recipient bladder cancer cells, their selective enrichment in regulatory proteins, such as H3.2 (H3C14), LAMB1 and CD147, suggests an indirect role in maintaining chemoresistance. These vesicles may contribute to resistance by sequestering key intracellular proteins, thereby modifying the tumour microenvironment and stabilizing drug‐resistant phenotypes.

To further investigate the distribution of surface markers and cargo‐associated proteins within EV subtypes, we used imaging flow cytometry to analyse 10,000 individual EVs labelled with CD9, CD63, CD81, LAMB1 and CD147 (Figure 7F–J). Unlike western blot analysis, which measures bulk protein levels, imaging flow cytometry provides single‐EV resolution, enabling the identification of rare but functionally significant vesicle subpopulations. To evaluate the correlation between typical exosomal tetraspanins (CD9, CD63 and CD81) and the potential Excretion‐EVs markers CD147 and LAMB1, scatter plots of any two of the five markers were analysed. Fewer CD9⁺ and/or CD81⁺ EV subpopulations were observed in T24GCB‐derived EVs (T24GCB EVs) than in T24‐derived EVs (T24 EVs). However, the CD63⁺ EV subpopulation was similar between the two groups. Notably, EVs derived from H3C14‐overexpressing T24GCB (TG‐H3C14 EVs) exhibited a greater compensatory increase in CD9^+^ and CD81^+^ EV subpopulations than those from T24GCB‐derived EVs. This suggests that GCB resistance acquisition may suppress certain CD9⁺ or CD81⁺ EV subpopulations, which are partially restored following H3C14 overexpression (Figure 7F).

Additionally, CD147⁺, LAMB1⁺ or both CD147⁺/LAMB1⁺ EV subpopulations were slightly higher in T24GCB EVs but dramatically enriched in TG‐H3C14 EVs than in T24 EVs. This suggests that excess intracellular H3.2 (H3C14) protein in a GCB‐resistant background induces a compensatory increase in the CD147⁺/LAMB1⁺ EV subpopulation, facilitating the packaging and excretion of excess H3.2 (H3C14) protein. Notably, the CD63⁺/CD147⁺ EV subpopulation was significantly higher in T24GCB EVs than in T24 EVs, whereas the CD63⁺/LAMB1⁺ EV subpopulation remained unchanged between the two groups (Figure 7G). These findings suggest that CD63⁺/CD147⁺ EVs are selectively enriched in the GCB‐resistant state and contribute to the establishment of a chemoresistant tumour microenvironment. Although prior studies have independently linked CD63⁺ or CD147⁺ EVs to therapy resistance, our results imply that the CD63⁺/CD147⁺ EV subpopulation is critically involved in GCB‐resistant bladder cancer. Furthermore, CD9⁺/CD147⁺ and CD81⁺/CD147⁺ EVs exhibited a greater reduction in T24GCB EVs than in T24 EVs, whereas TG‐H3C14 EVs demonstrated increased expression of CD9⁺/CD147⁺, CD81⁺/CD147⁺, CD9⁺/LAMB1⁺ and CD81⁺/LAMB1⁺ EVs (Figure 7H, I). These correlated findings support the notion that CD9^+^ and CD81^+^ EVs were less abundant in T24GCB EVs and compensated by H3C14 overexpression. Besides, H3C14 overexpression induced a compensatory increase in CD147⁺ and LAMB1⁺ EVs. Together, these results highlight the functional heterogeneity of EV subpopulations and suggest that CD63⁺/CD147⁺ EVs play a key role in GCB resistance.

## 4 Discussion

GCB resistance has emerged as a critical challenge in the treatment of bladder cancer, particularly following chemotherapy. However, the underlying mechanisms driving the initiation and promotion of GCB resistance remain poorly understood. In this study, GCB‐resistant bladder cancer cell models (T24GCB and 5637GCB) were used to investigate the molecular basis of GCB resistance. Although previous studies have primarily focused on the dysregulation of GCB‐metabolizing enzymes and nucleoside transporters, our findings revealed that EVs secreted by GCB‐resistant cells can confer resistance to GCB‐sensitive recipient cells. This effect is mediated through the modulation of nucleoside transporter and metabolizing enzymes expression.

Proteomic analysis of EV content revealed a significant increase in histone H3.2 (H3C14) in EVs derived from GCB‐resistant cells, accompanied by a corresponding decrease of H3C14 within the resistant cells. Notably, H3C14 has previously been implicated in gastric cancer progression via the EGFR–FOXC1 signalling axis (Rashid et al. 2021). Our findings further demonstrated that H3C14 modulates key proteins involved in nucleoside transporter and metabolism, ultimately impairing GCB uptake and activation and thereby contributing to the development of GCB resistance.

Moreover, Rab27A knockdown using siRNA, as well as pharmacological inhibition with small‐molecule compounds such as neticonazole and ketoconazole, resulted in a marked reduction in EV secretion. This was accompanied by intracellular accumulation of histone H3.2 (H3C14), which alleviated GCB resistance, supporting the established role of Rab27 in EV‐mediated tumour progression (Li et al. 2018; Koh et al. 2020). Knockdown of Rab27A with siRab27A significantly increased H3.2 (H3C14) protein and mildly increased CNT3 protein in GCB‐resistant cells. And knockdown of Rab27A also mildly increased Alix and CD3 and reduced TSG101 protein levels in T24GCB; however, those proteins were indistinguishable between 5637GCB cells transfected with siRab27A and vector control (Figure 6G). In previous studies, Rab27A levels have been correlated with Alix, CD63 and TSG101 in the intracellular expression levels and contents of EVs, but positive‐ or negative‐correlation is various in different cellular context and exosomal subset. In sum, Rab27A knockdown increased TSG101 but did not change levels of CD63 and Alix in EVs of human SkMel28 melanoma cell, but Rab27A knockdown increased TSG101 and CD63 but did not change level of Alix in EVs of human A375 melanoma cell (Horodecka et al. 2024). And Rab27A knockdown or knockout also increased TSG101 and CD63 in EVs of human WM164 and murine B16–F10 melanoma cells (Guo et al. 2019). Rab27A knockdown decreased TSG101, CD63 and Alix in EVs of murine 4T1 breast cancer cell (“Scientific Program 2012 ISEV meeting Wednesday 18th April,” 2012), Rab27A knockdown decreased CD63 and Alix in EVs of Ad293 cell (Oshima et al. 2019) but no effect in EVs of HEK293 cells (Fordjour et al. 2022). Therefore, it implicates that Rab27A‐mediated changes of Alix, CD63 or TSG101 are higher dependent on cell or EVs types than those drug‐resistance induced effects. These findings underscore the critical role of EV‐mediated H3.2 (H3C14) excretion, likely facilitated by the ESCRT machinery, in the initiation and promotion of GCB resistance. Further studies are required to elucidate the specific pathways governing H3.2 (H3C14) sorting and excretion.

Histone H3 modifications have been implicated in GCB resistance across various cancer types. In pancreatic cancer cells, H3K4me3 is associated with the activation of anti‐apoptotic genes, such as Bcl‐x, FLIP and Mcl‐1 and H3K9me3 is linked to the suppression of pro‐apoptotic genes, including Bak, Bax and Bim, contributing to GCB resistance (Lu et al. 2018). In cervical cancer cells, demethylation of H3K9 (H3K9m2) at the hENT1 and dCK promoters correlates with GCB resistance via the upregulation of G9A, a histone methyltransferase (Candelaria et al. 2012). Additionally, histone H3 acetylation at lysine 9 and 27 (H3K9Ac and H3K27Ac) promotes ribonucleotide reductase large subunit M1 (RRM1)‐dependent invasion and extracellular matrix remodelling (Ono et al. 2023) and enhances acetylation at the matrix metalloproteinase‐10 promoter (Shimizu et al. 2017) in GCB‐resistant pancreatic cancer cells. Clinically, higher levels of H3K4me2 have been associated with better GCB responses in Japanese patients with pancreatic cancer (Watanabe et al. 2012). Notably, histone H3.1 and H3.2 differ at residue 96, with H3.1 containing cysteine (Cys96) and H3.2 containing serine (Ser96). However, histone H3 modifications associated with GCB resistance have not been reported at this specific residue. Early quantitative mass spectrometry analyses of human H3 variants revealed that H3.2 is predominantly enriched in repressive markers, such as H3.2K27me2, H3.2K27me3 and H3.2K36me, which are characteristic of facultative heterochromatin. In contrast, H3.1 displays a broader spectrum, enriched in both active (e.g., H3.1K14Ac) and repressive marks (e.g., H3.1K9me2 and H3.1K64me) modifications (Hake et al. 2006). Furthermore, chemotherapy‐related oxidative stress induced S‐glutathionylation at Cys96 of histone H3.1, contributing to chromatin compaction and playing a role in redox balance and gene expression regulation (García‐Giménez et al. 2024). Recent advances have emphasized the distinct functional roles of histone H3 variants in modulating therapy response. Notably, histone H3.1 acts as a chromatin‐embedded redox sensor that promotes adaptive phenotypic plasticity and multidrug resistance in cancer cells. Notably, mutation of cysteine 96 to serine (C96S) abolished the ability of H3.1 to confer drug resistance (Palma et al. 2024). These findings suggest that subtle structural differences between histone variants may underlie divergent redox‐sensing capacities and drug resistance functions. Given that our study identified histone H3.2 (H3C14) as an anti‐GCB resistance histone variant selectively excreted via EVs, this raises the possibility that redox sensitivity may be involved in the sorting of histones into EVs. However, the lack of antibodies capable of discriminating between histone H3.1 and H3.2 variants, owing to the single amino acid difference, limits the ability to fully understand these underlying interactions. Future investigations utilizing variant‐specific proteomic tools and targeted mutagenesis will be essential to unravel the functional interplay between histone subtype composition, EV‐mediated sorting and chemoresistance.

In our study, we uncovered the heterogeneity of EV subpopulations and their distinct contributions to GCB resistance in bladder cancer. By isolating CD9⁺, CD63⁺, and/or CD81⁺ EVs—collectively termed as Transport‐EVs—from GCB‐resistant cells, we demonstrated their ability to transfer GCB‐resistant phenotypes to otherwise sensitive recipient cells. In contrast, the CD9^−^/CD63^−^/CD81^−^ EVs (termed as Excretion‐EVs) did not directly induce GCB resistance but were enriched with key proteins, such as H3.2 (H3C14), CD147 and LAMB1. This suggests a potential role of Excretion‐EVs in the selective excretion of regulatory molecules from GCB‐resistant cells. These findings offer new insights into the multifaceted role of EVs in chemoresistance: Transport‐EVs actively disseminate GCB resistance signals, and Excretion‐EVs may function as a cellular disposal mechanism for factors that suppress malignancy or enhance GCB sensitivity. This hypothesis is supported by our observation that H3C14 overexpression reduces both GCB resistance and tumour aggressiveness, suggesting that its excretion via Excretion‐EVs may represent an adaptive strategy of GCB‐resistant cells to maintain their survival and aggressiveness.

Tetraspanins, such as CD9, CD63 and CD81, are canonical surface markers commonly used to define exosome populations; however, their functional roles extend beyond structural classification. Increasing evidence indicates that EVs enriched in these markers actively contribute to drug resistance and tumour progression. For instance, CD9 has been implicated in mediating EV uptake and facilitating metastasis. In breast cancer, chemotherapy stimulates CD9⁺ EV secretion, which promotes pre‐metastatic niche formation and enhances metastatic spread (Keklikoglou et al. 2019). Similarly, CD9 facilitates the uptake of fibroblast‐derived EVs by pancreatic cancer cells, thereby enhancing their aggressiveness and suggesting a role in shaping the tumour microenvironment (Nigri et al. 2022). Additionally, CD63 has been directly implicated in therapy resistance. Ma et al. demonstrated that vascular endothelial growth factor (VEGF) can be encapsulated within CD63⁺ EVs, allowing it to evade anti‐VEGF therapeutic antibodies and contribute to resistance in hepatocellular carcinoma (Ma et al. 2021). Supporting this, Kim et al. reported that engineered EVs displaying CD63 along with VEGFR‐binding peptides improved selective drug delivery, highlighting a functional role for CD63 in EV trafficking and cargo targeting (Kim et al. 2025). CD81 has been implicated in the regulation of cancer stemness and therapy resistance. In acute lymphoblastic leukaemia cells, CD81 knockout resulted in increased chemosensitivity and impaired engraftment, suggesting that CD81 supports the maintenance of resistant phenotypes via EV‐mediated mechanisms (Quagliano et al. 2020). Furthermore, Ramos et al. revealed that CD81 interacts with CD44 to maintain cancer stemness and EV structural integrity, underscoring its critical role in EV biogenesis and resistance propagation (Ramos et al. 2022). Additionally, CD81‐positive EVs have been used as efficient carriers for drug delivery in cancer models, reflecting their capacity for selective cargo packaging and targeted delivery (Huang et al. 2024). In our study, although CD9⁺, CD63⁺ and CD81⁺ EVs did not directly carry histone H3.2 (H3C14), their prevalence and enrichment in drug‐resistant cells suggest a potential role in mediating GCB resistance‐related signalling. These canonical EVs may function in tandem with CD147⁺ or LAMB1⁺ EVs, either by facilitating resistance transfer or by compensatory excretion of tumour‐suppressive cargos. Understanding the interplay between canonical and non‐canonical EV subpopulations will be essential for unravelling the complex EV‐mediated GCB resistance networks in bladder cancer.

Moreover, CD63⁺/CD147⁺ EVs were significantly elevated in T24GCB cells, implicating this subtype in the establishment of a GCB‐resistant microenvironment. Previous studies have linked both LAMB1 and CD147 to cancer metastasis and EV biogenesis. Our findings suggest that these markers define functionally distinct EV subpopulations in GCB‐resistant bladder cancer. Notably, both proteins are increasingly recognized as active contributors to tumour progression through their association with EVs. Supporting this, Pang et al. (2024) identified LAMB1 as a small EV (sEV) protein significantly enriched in the plasma of patients with metastatic prostate cancer, highlighting its potential as a biomarker for diagnosis and risk stratification (Pang et al. 2024). In addition, CD147 has been implicated in promoting cancer aggressiveness through EV‐mediated mechanisms. Boddu et al. (2024) demonstrated that urinary EVs from patients with MIBC exhibited higher levels of CD147 and increased pro‐thrombotic activity than did those with non‐invasive disease, reinforcing its clinical significance (Boddu et al. 2024). Beyond surface expression, CD147 may regulate EV cargo loading. Ko et al. (2023) reported that CD147 interacts with heterogeneous nuclear ribonucleoprotein A2/B1 (hnRNPA2B1), facilitating the selective enrichment of microRNAs within CD147⁺ EVs. Besides, elevated levels of circulating CD147⁺ EVs have been observed in patients with ovarian and renal cancers, further supporting its role as a cancer‐associated EV marker (Ko et al. 2023; Zhang et al. 2023). LAMB1 and CD147 have been correlated with different drug resistance in several cancer types; however, none of them contribute to resistance by sequestering key intracellular proteins. In short, LAMB1 decreases sensitivity to temozolomide in glioma cells by activating the NFκB/HK2 signalling axis mediated aerobic glycolysis (Zhao et al. 2025). LAMB1 also decreased sensitivity to lapatinib in HER2‐positive breast cancer cells by activating ERK1/2 phosphorylation and vimentin expression and enhancing cell viability (Gurung et al. 2024). Moreover, exosomal LAMB1 may serve as a cisplatin‐resistant biomarker in mesenchymal high‐grade serous ovarian carcinoma (Ferreira et al. 2023); however, there is no study about LAMB1 on EVs‐modulated drug‐resistant regulations in cancer treatment. Relatively, CD147 is more comprehensively correlated with drug resistance in several cancer cells (Landras et al. 2019). In sum, CD147 induces 5‐FU resistance by promoting HIF 1α/PI3K/AKT/mTOR axis–driven glycolysis and suppresses PPARα/MAPK axis‐mediated fatty acid oxidation in colorectal cells (Dong et al. 2022), or by stabilizing XIAP to reduce apoptosis in oral squamous carcinoma cells (Kuang et al. 2009). CD147 induces epirubicin/docetaxel resistance by interacting with vacuolar H⁺ ATPase and thereby increasing its activity to acidify the tumour microenvironment in breast cancer cells (Kuang et al. 2018). CD147 induces temozolomide resistance by suppressing GSK3β/β‐TrCP‐dependent Nrf2 protein degradation and ablating temozolomide induced ROS production in glioma cells (Bu et al. 2021). CD147 induced multidrug resistance by upregulating ABC transporters to facilitate drug efflux in leukaemia cells (Somno et al. 2016) and pancreatic cancer cells (Bahmed et al. 2013). And CD147‐mediated chemoresistance is also reported by activating MAPK/ERK signalling pathway (Ma et al. 2017), or collaborates with other membranous proteins, such as CD44, hyaluronan and so forth in some cancer cells (Landras et al. 2019). However, there is also no study about CD147 on EVs‐modulated drug‐resistant regulations in cancer treatment. Consistent with these findings, our imaging flow cytometry analysis revealed that CD63⁺/LAMB1⁺ and CD63⁺/CD147⁺ EVs were significantly higher in T24GCB cells than in their parental counterparts. These EV subtypes may contribute to the establishment of a drug‐resistant tumour microenvironment through targeted intercellular communication. Moreover, EVs derived from H3C14‐overexpressing cells (TG‐H3C14‐EVs) altered the overall composition of EV surface markers, suggesting that H3C14 levels can affect EV secretion dynamics and cargo selection. These findings support a model in which GCB‐resistant bladder cancer cells leverage both EV‐mediated signalling and selective excretion of regulatory proteins to enhance survival under chemotherapeutic stress. The identification of Excretion‐EVs as carriers of tumour‐suppressive proteins, such as H3.2 (H3C14), CD147 and LAMB1, highlights an underexplored mechanism of GCB resistance involving the active excretion of unwanted intracellular regulators via EV secretion. Targeting EV biogenesis or sorting mechanisms such as Rab27A inhibition or the interactions between CD63, LAMB1 and CD147 could provide novel therapeutic avenues to reverse resistance and re‐sensitize tumours to GCB.

As previously described, dysregulation of GCB‐metabolizing enzymes and nucleoside transporters plays a central role in the development of GCB resistance. Among these enzymes, DCK downregulation is particularly associated with reduced GCB sensitivity (Bergman et al. 2002). Moreover, exosome‐delivered miR‐155 inhibits DCK expression and impairs reactive oxygen species detoxification, thereby promoting GCB resistance in pancreatic cancer cells (Patel et al. 2017). Although findings remain somewhat controversial, TK2 downregulation has been implicated in GCB resistance in bladder cancer cells. (Beauséjour et al. 2002). Conversely, elevated expression of NT5C2 promotes resistance by dephosphorylating dCdCMP, thereby impairing GCB activation (Alvarellos et al. 2014). Regarding GCB transporters, uptake is primarily mediated by ENT1, with lesser contributions from concentrative nucleoside transporters CNT1 and CNT3. In general, ENT and CNT activity enhances GCB influx and improves drug sensitivity in most cancer models, although some conflicting reports exist (Alvarellos et al. 2014; Greenhalf et al. 2014; Liu et al. 2014; Zhang et al. 2007). The well‐documented ENT1 has been complicated by its controversial therapeutic implications. Although ENT1 enhances GCB influx and restores GCB sensitivity in most preclinical studies, higher ENT1 expression levels have been associated with better GCB responses and prognosis in various cancer types (McKenna et al. 2025), including bladder cancer (Matsumura et al. 2011). However, several conflicting reports have emerged regarding its exact role in GCB resistance (Boyd et al. 2023). Additionally, downregulation of CNT1 was reported to induce GCB resistance (Hung et al. 2015; Bhutia et al. 2011).

In particular, CNT3 was significantly upregulated in both T24GCB and 5637GCB cells, with further induction observed in T24 and 5637 cells after exposure to GCB‐resistant cell‐derived ConMed in this study (Figures 1I and 2Q). Although CNT3 is typically associated with enhanced nucleoside analogue uptake and increased drug sensitivity, emerging evidence suggests that its role in chemoresistance may be more complex. For example, Ibarra and Pfeiffer reported that CNT3‐mediated drug resistance reduced the antiviral efficacy of ribavirin by altering its intracellular trafficking (Ibarra and Pfeiffer 2009). Similarly, in chronic lymphocytic leukaemia, differential CNT3 expression was observed between fludarabine‐sensitive and resistant patient subgroups, suggesting that CNT3 plays a role in modulating drug responsiveness (Mackey et al. 2005). Recent cryo‐electron microscopy (cryo‐EM) structural analyses have revealed that certain CNT3 variants may localize to the endoplasmic reticulum (ER) rather than the plasma membrane (Zhou et al. 2020). This subcellular mislocalization raises the intriguing possibility that CNT3 contributes to EV biogenesis or cargo sorting within the ER‐Golgi network. Given that EV formation often involves ER‐derived membranes and EV composition is tightly regulated, our observation that CNT3 is preferentially packaged into CD81⁺ EVs (Figure S8) suggests a potential role in shaping the protein cargo profile of GCB resistance‐associated EVs. In contrast to TK1, NT5C2 and CNT1—whose regulation was either modest or inconsistent—CNT3 exhibited consistent upregulation in GCB‐resistant cells and their derived EVs, highlighting its unique functional relevance. Collectively, these findings highlight CNT3 as a previously underappreciated regulator of GCB resistance and EV‐mediated intercellular communication in bladder cancer.

Although imaging flow cytometry provides unique advantages in single‐particle profiling of EVs, several technical limitations must be considered. First, its high sensitivity is especially beneficial for identifying rare but functionally important EV subpopulations, such as CD63⁺/LAMB1⁺ vesicles. However, this sensitivity requires strict control over sample purity and antibody specificity to minimize false positives. Second, the sample preparation and staining protocols for EVs remain technically challenging. Current permeabilization methods are often insufficient for reliable detection of intraluminal cargo proteins, such as H3.2 (H3C14) protein, limiting our ability to confirm cargo localization within vesicles. Third, imaging flow cytometry experiments must be conducted using ultrapure, particle‐depleted buffers (e.g., 0.02 µm‐filtered PBS) to prevent artifacts from protein aggregates or background particles. Fourth, most commercial imaging flow cytometry instruments have a spectral resolution that typically allows for a maximum of six fluorescent channels, limiting the multiplexing capacity for surface and cargo markers. In summary, although imaging flow cytometry provides valuable resolution in analysing EV heterogeneity and marker co‐localization at the single‐vesicle level, its present limitations in membrane permeabilization and fluorescence channel capacity highlight the need for further methodological improvements. These advances will be essential for fully exploiting the potential of imaging flow cytometry in characterizing both the surface and luminal components of EVs involved in drug resistance and tumour progression.

Although the use of MicroBeads Pan targeting CD9, CD63 and CD81 allowed us to selectively isolate tetraspanin‐positive EV subpopulations and verify their uptake by recipient cells (Figure 7B), technical constraints limited our ability to functionally characterize other EV subpopulations. Specifically, we attempted to use magnetic beads conjugated with anti‐CD147 or anti‐LAMB1 antibodies to isolate CD147⁺ or LAMB1⁺ EVs. Although this approach successfully enriched these EVs for western blot‐based identification, the size of traditional magnetic beads (in the micron range) hindered effective EV uptake by recipient cells. Therefore, these bead‐bound EVs could not be used for subsequent functional assays, such as evaluating H3.2 (H3C14) transfer or cellular phenotypic changes. This limitation highlights the requirement for alternative strategies, such as antibody‐based capture with nanobeads or affinity microfluidics, to better assess the therapeutic relevance and biological functions of marker‐specific EV subpopulations.

**FIGURE 8:**
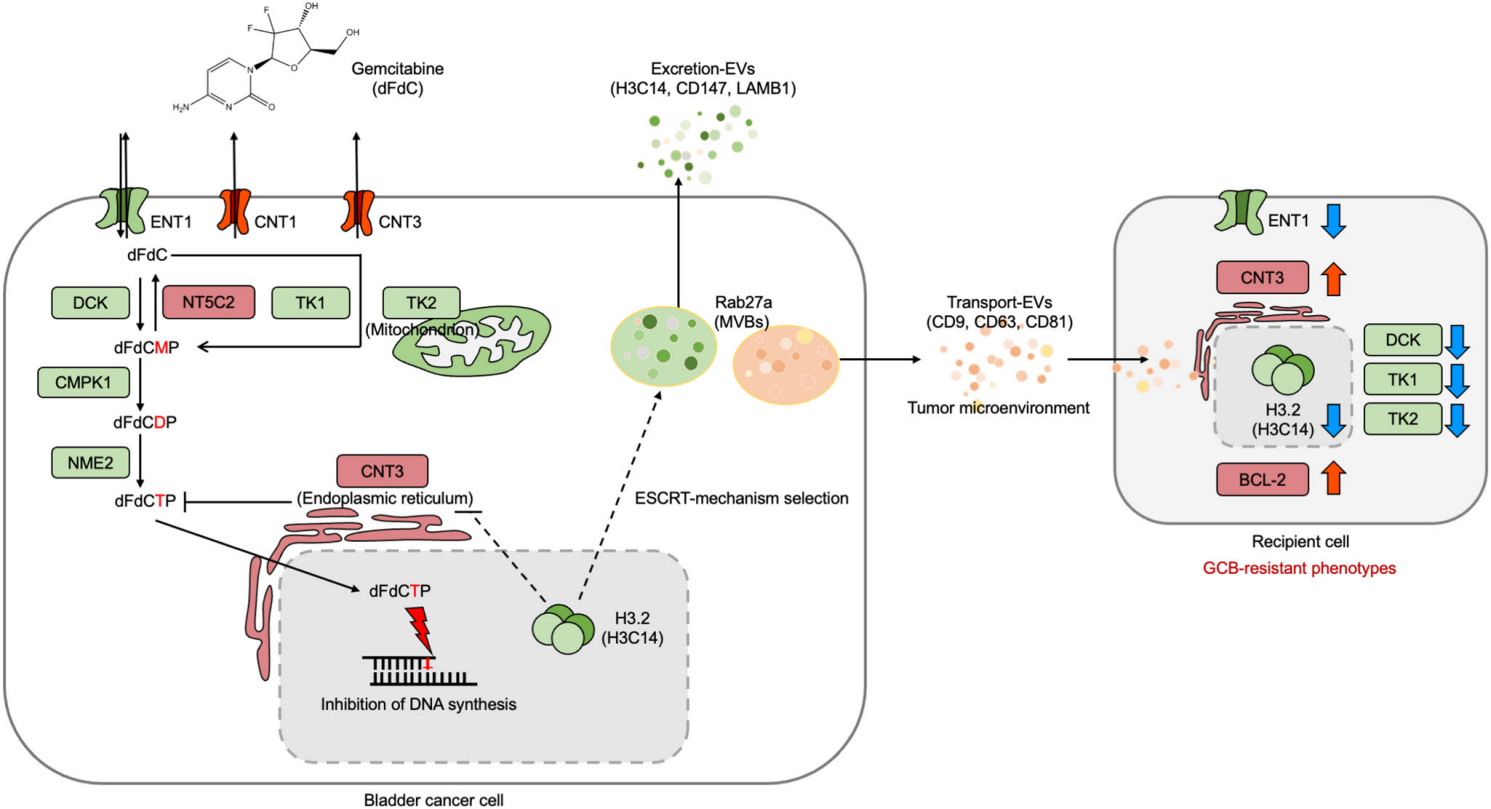
**Schematic diagram depicting the H3C14‐mediated regulation of GCB metabolism and dynamics of EV subtypes in bladder cancer**. Gemcitabine (GCB, dFdC) enters the bladder cancer cells via the equilibrative nucleoside transporter ENT1. After internalization, it undergoes sequential phosphorylation by DCK, CMPK1 and NME2 to form its active metabolite dFdCTP (dFdC → dFdCMP → dFdCDP → dFdCTP), which inhibits DNA synthesis in the nucleus. Alternatively, GCB can be phosphorylated through TK1 in the cytosol or TK2 in the mitochondrion. In GCB‐resistant cells, CNT1 and CNT3—localized at the plasma membrane and endoplasmic reticulum, respectively—are upregulated to facilitate drug efflux. NT5C2 further contributes to resistance by dephosphorylating GCB metabolites. Under normal conditions, the histone variant H3.2 (H3C14) suppresses CNT3 expression. However, in resistant cells, H3.2 (H3C14) is selectively excreted via the ESCRT machinery into MVBs and secreted extracellularly through EVs. These EVs can be classified into two major subtypes: (1) Transport‐EVs, marked by CD9, CD63 and/or CD81, which propagate GCB‐resistant phenotypes to recipient cells by shaping a pro‐tumorigenic microenvironment; (2) Excretion‐EVs, which lack CD9, CD63 and CD81 but are enriched in CD147 and LAMB1, thereby promoting GCB resistance by excreting tumour‐suppressive regulators from the GCB‐resistant cells. GCB, gemcitabine; EVs, extracellular vesicles; MVBs, multivesicular bodies.

In conclusion, we identified a novel mechanism by which GCB‐resistant bladder cancer cells reshape the tumour microenvironment through selective histone export via EVs. Our findings highlight histone H3.2 (H3C14) protein as a key variant that modulates GCB metabolism and tumour progression. GCB‐resistant cells actively excrete H3.2 (H3C14) through Rab27A‐dependent EV biogenesis and secretion, thereby reducing its intracellular levels and contributing to therapeutic resistance. Notably, gain‐of‐function of H3C14 restores GCB sensitivity, whereas its loss promotes a more aggressive cancer phenotype and apoptosis resistance. Additionally, we demonstrated that specific EV subpopulations—particularly those marked by CD147 and LAMB1—are closely associated with GCB resistance and may serve as biomarkers for GCB refractory disease. Pharmacological inhibition of Rab27A or knockdown of CNT3 significantly reduced EV production, restored intracellular H3.2 (H3C14) levels and enhanced GCB sensitivity, suggesting promising therapeutic strategies (Figure 8). Altogether, our study highlights the critical role of EV‐mediated histone dynamics in GCB resistance and identifies H3C14 and its trafficking pathways as actionable targets for overcoming chemoresistance in bladder cancer.

## Author Contributions

**Cheng‐Shuo Huang**: conceptualization, investigation, validation, writing–review and editing, writing–original draft, project administration, software, methodology, formal analysis, data curation, visualization. **Dah‐Shyong Yu**: conceptualization, funding acquisition, writing–original draft, project administration, resources, writing–review and editing, supervision, validation. **Shih Sheng Jiang**: methodology, investigation, data curation, visualization, writing–review and editing. **Ying‐Si Wu**: methodology, validation, formal analysis. **Jar‐Yi Ho**: writing–review and editing, writing–original draft, funding acquisition, conceptualization, resources, methodology, data curation, supervision, formal analysis, validation. **Cheng‐Ping Yu**: resources, writing–original draft, conceptualization, writing–review and editing, funding acquisition, project administration, supervision.

## Conflicts of Interest

The authors declare no competing interests.

## Supporting information




**Supplementary Figures**: jev270179‐sup‐0001‐FigureS1‐S8.pdf

**Supplementary Material**: jev270179‐sup‐0002‐SuppMatt.pdf

**Supplementary Material**: jev270179‐sup‐0003‐SuppMat.pdf

**Supplementary Material**: jev270179‐sup‐0004‐SuppMat.docx

**Supplementary Tables**: jev270179‐sup‐0005‐Table.docx

## Data Availability

All data relevant to this study are included in the article and its Supplementary Information.
