# Supplementary material for: Extracellular Vesicle‐Mediated Regulation of H3C14 Contributes to Gemcitabine Resistance in Bladder Cancer
Source: J Extracell Vesicles. 2025 Oct 29;14(11):e70179. doi: 10.1002/jev2.70179 (PMC12570045; doi:10.1002/jev2.70179)

Supplementary Figure 1

A

Data1: GCB 10μM Data4: GCB 0.5μM Data7: GCB 0.01μM
Data2: GCB 5μM Data5: GCB 0.1μM
Data3: GCB 1μM Data6: GCB 0.05μM

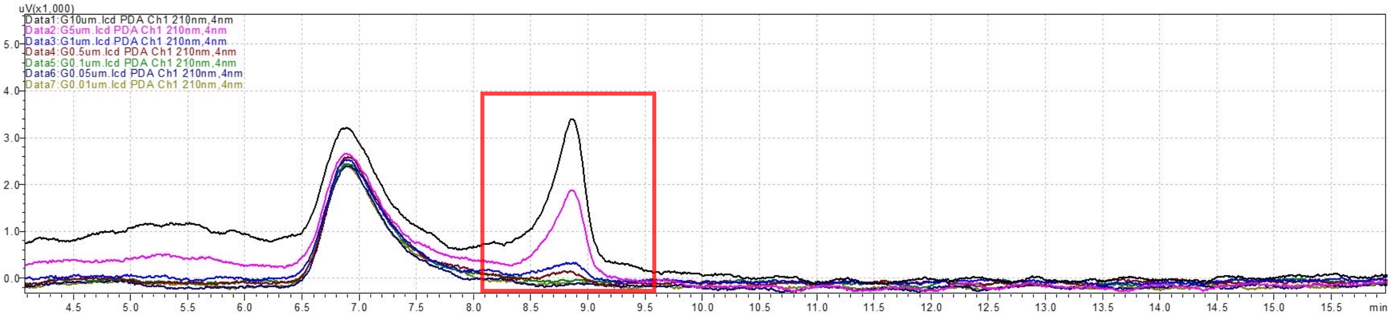

B

SP1: J82EVs + GCB 0.1μM SP3: T24EVs SP5: 5637EVs
SP2: J82EVs + GCB 0.05μM SP4: T24GCBEVs SP6: 5637GCBEVs

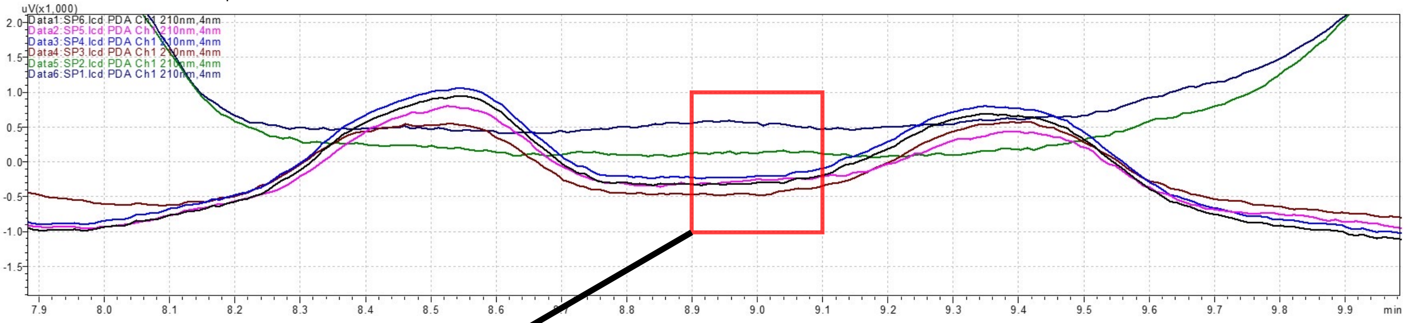

C

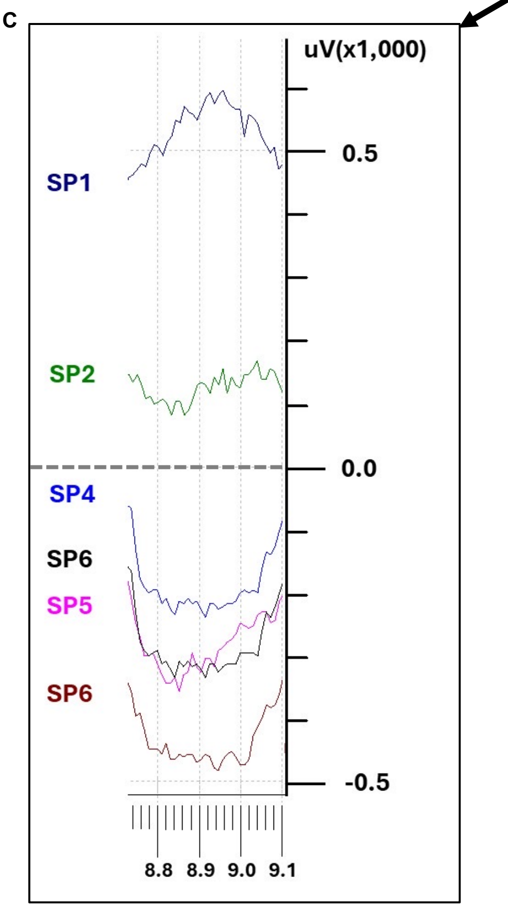

D

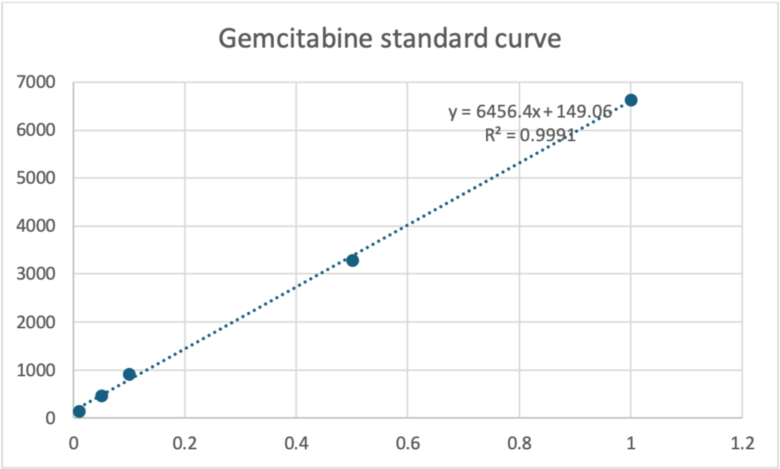

E

Sample ID	Gemcitabine (μM)
J82EVs + GCB 0.1μM	0.1025
J82EVs + GCB 0.05μM	0.0437
T24EVs	ND (<0.01)
T24GCBEVs	ND (<0.01)
5637EVs	ND (<0.01)
5637GCBEVs	ND (<0.01)

Supplementary Figure 2

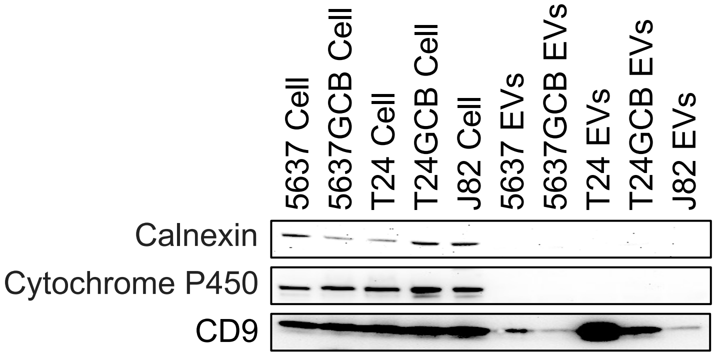

Supplementary Figure 3

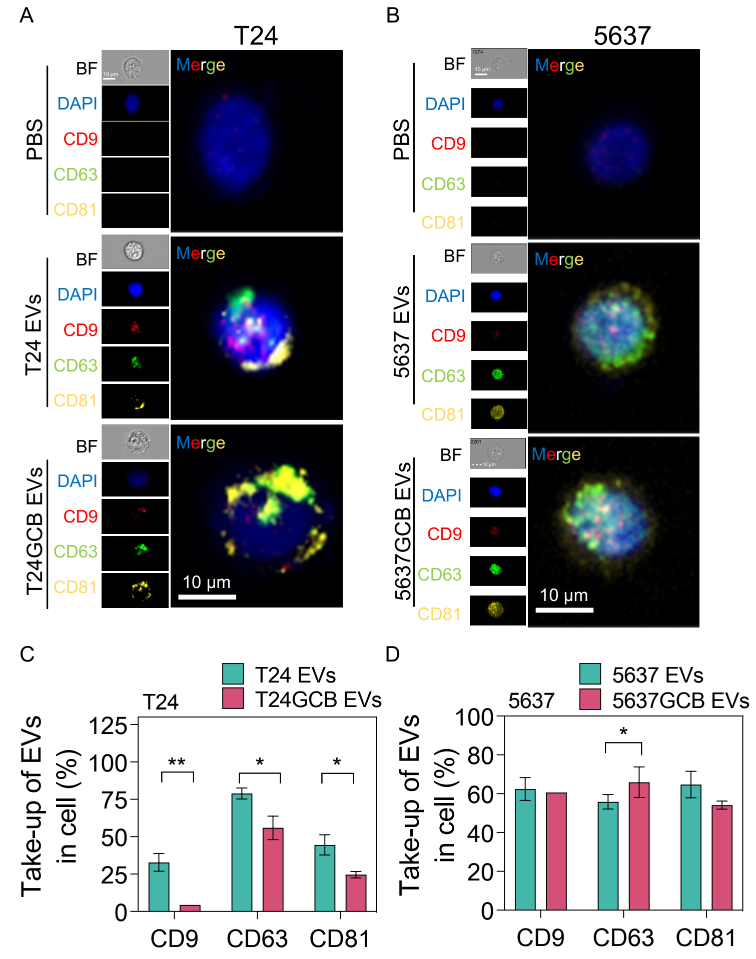

Supplementary Figure 4

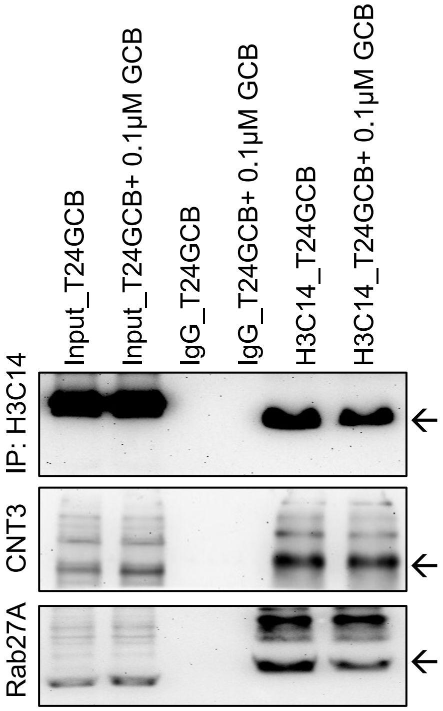

Supplementary Figure 5

A

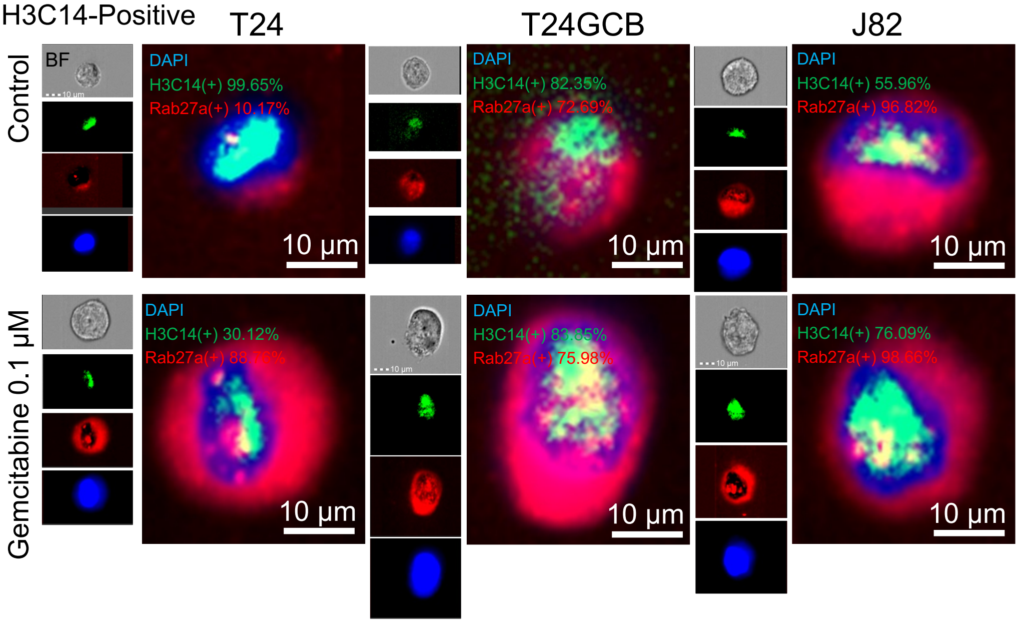

B

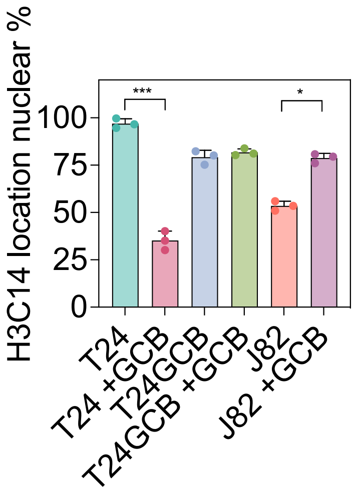

Supplementary Figure 6

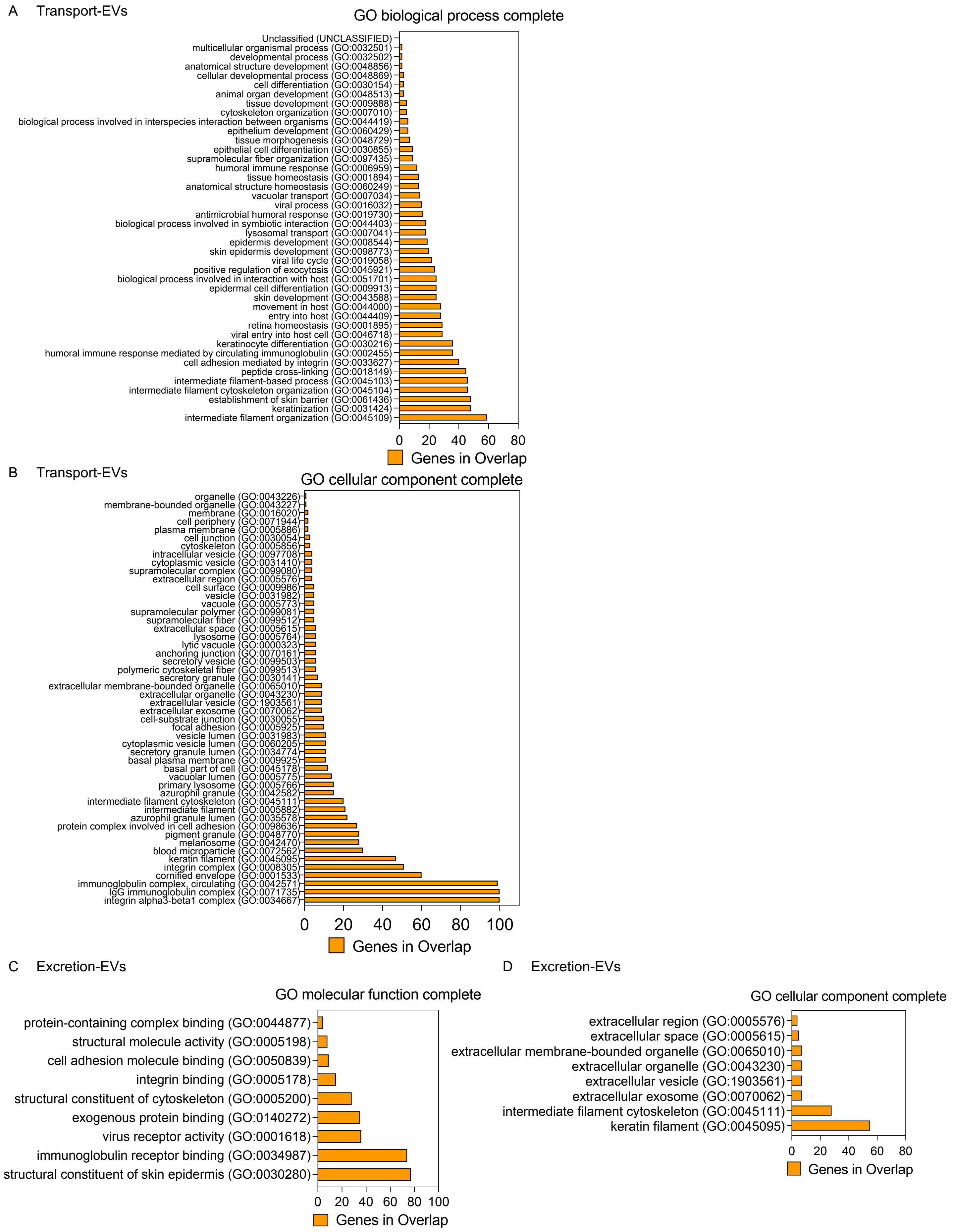

Supplementary Figure 7

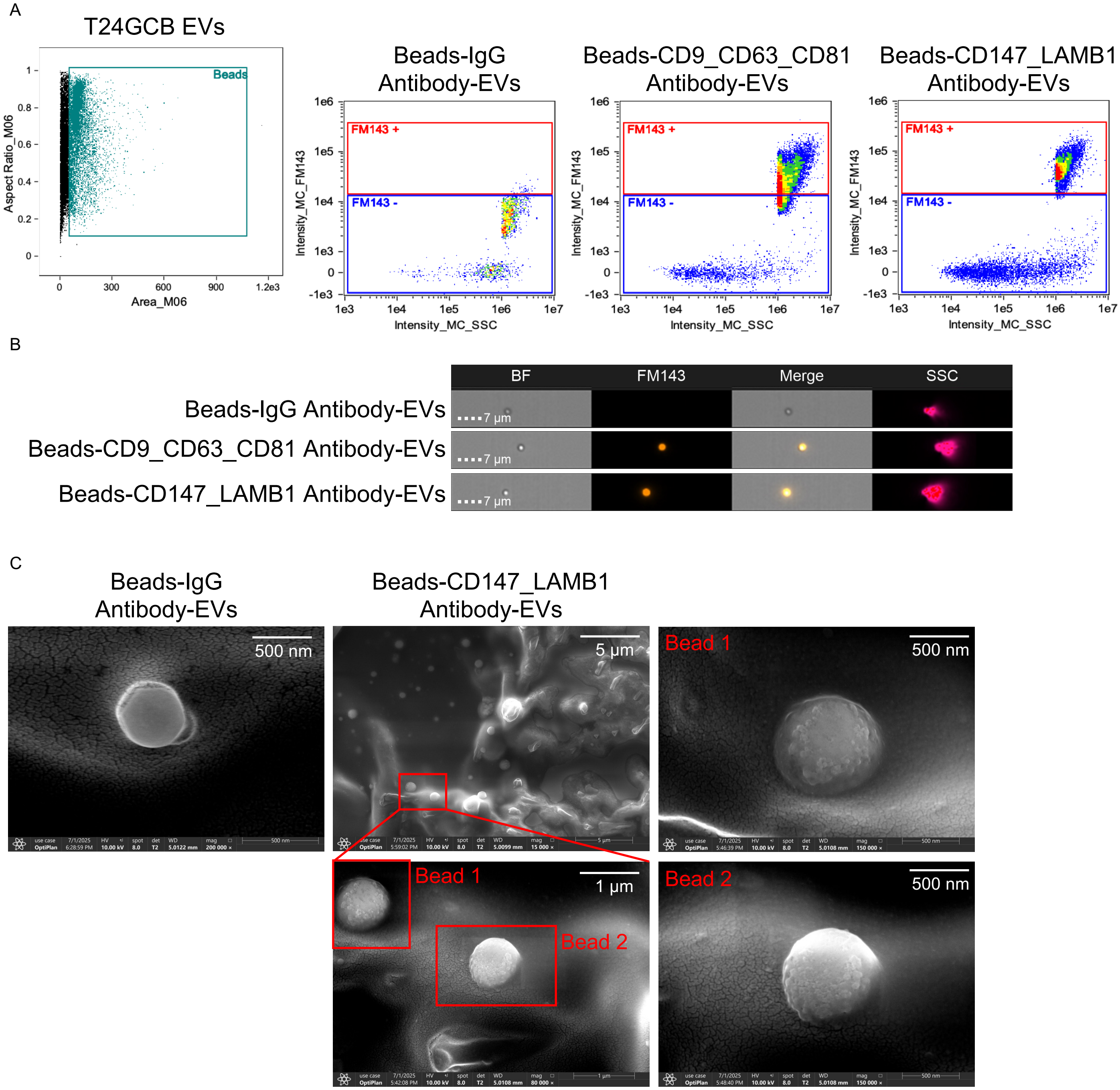

Supplementary Figure 8

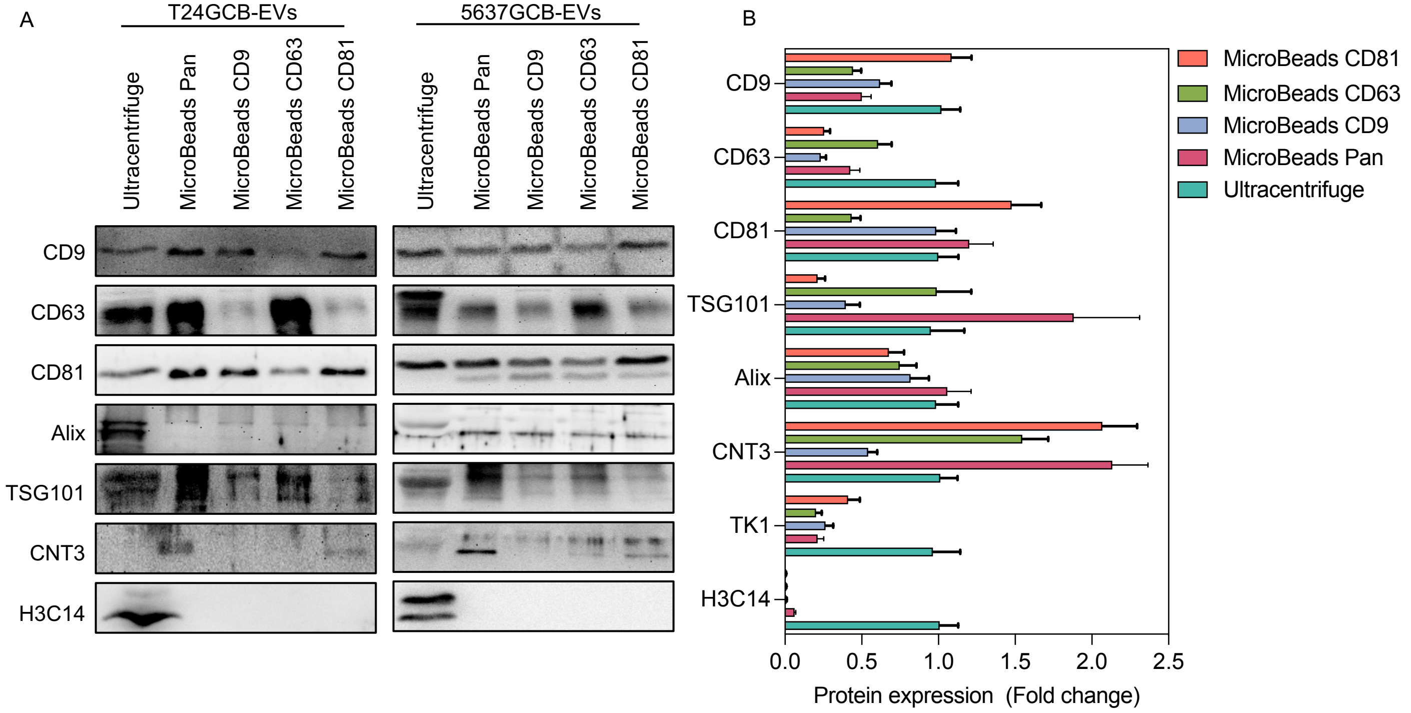

Supplement: Supplementary file 1 — Supplementary Figures: jev270179‐sup‐0001‐FigureS1‐S8.pdf [file JEV2-14-e70179-s002.pdf]
