# Supplementary figures and images for: Extracellular Vesicle‐Mediated Regulation of H3C14 Contributes to Gemcitabine Resistance in Bladder Cancer
Source: J Extracell Vesicles. 2025 Oct 29;14(11):e70179. doi: 10.1002/jev2.70179 (PMC12570045; doi:10.1002/jev2.70179)

Fig 11

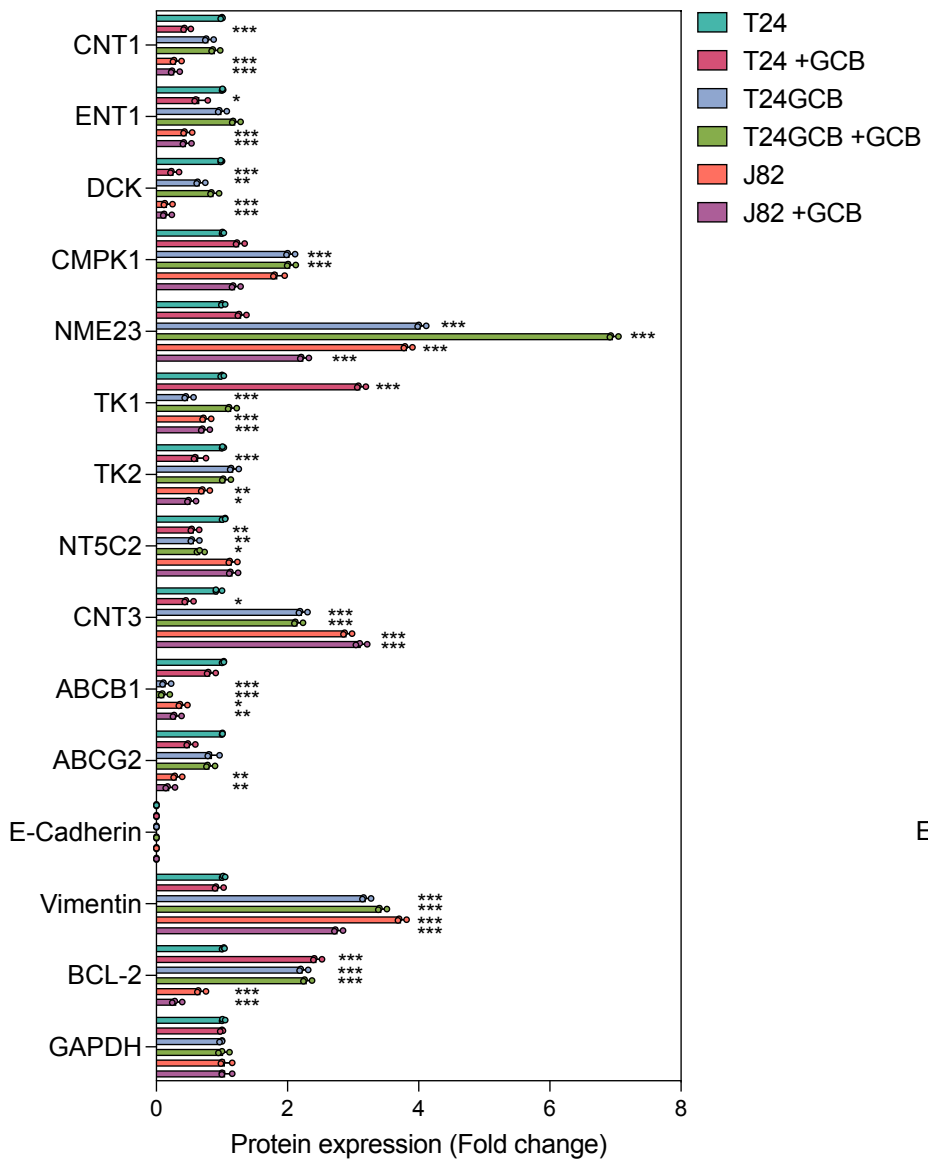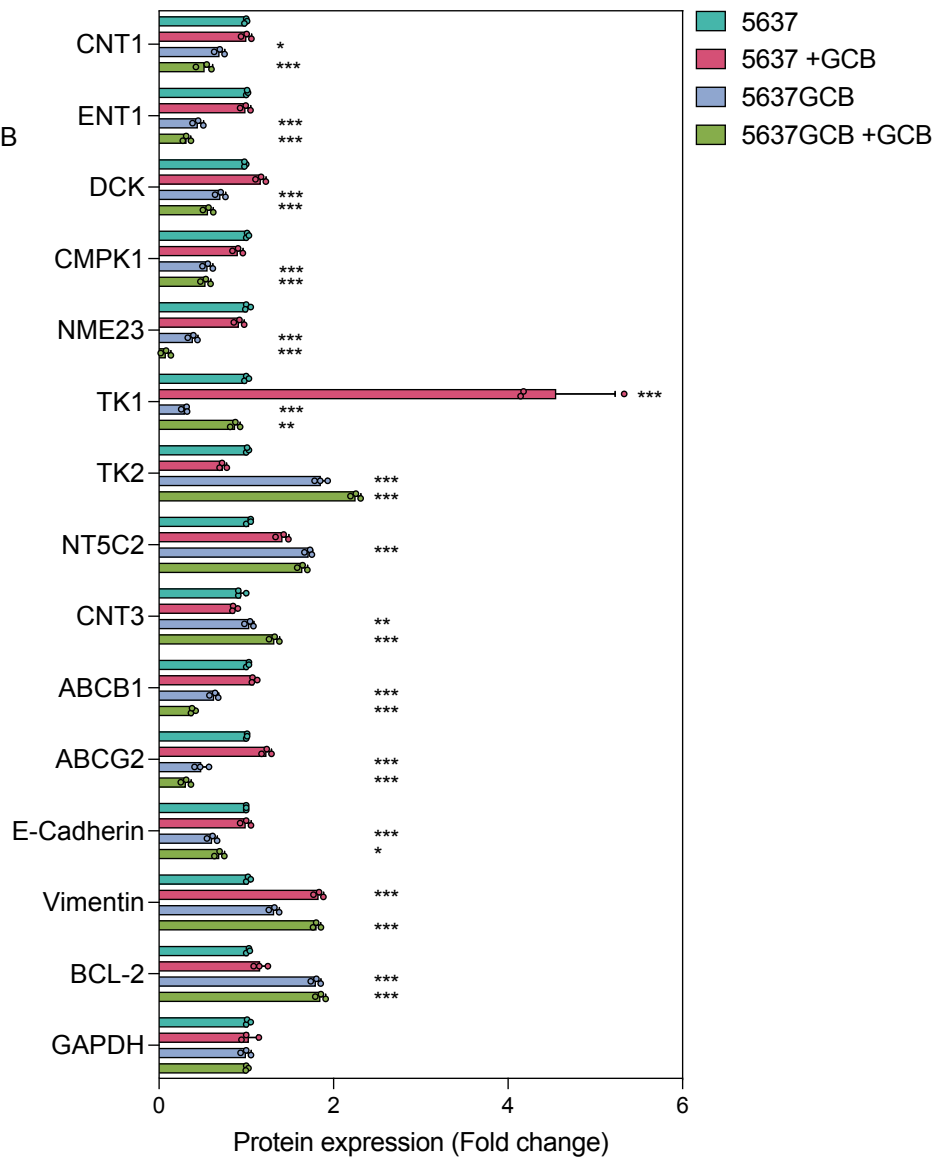

Fig 2F

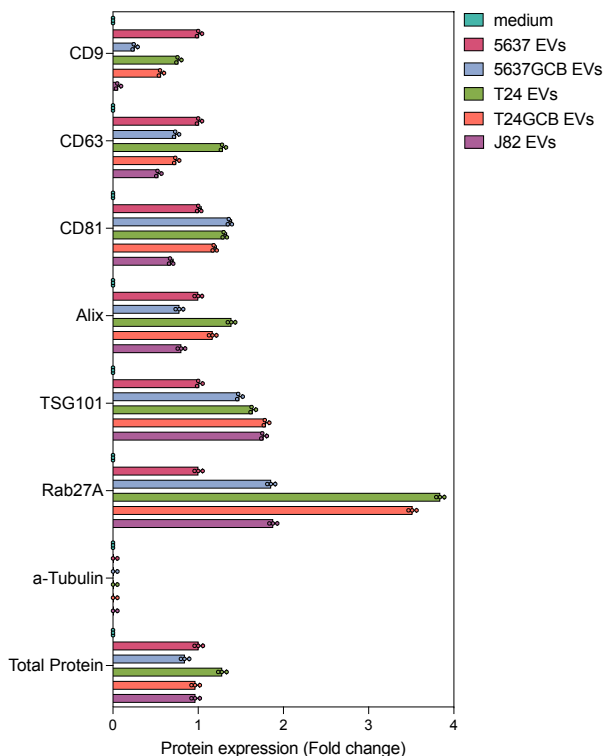

Fig 2J

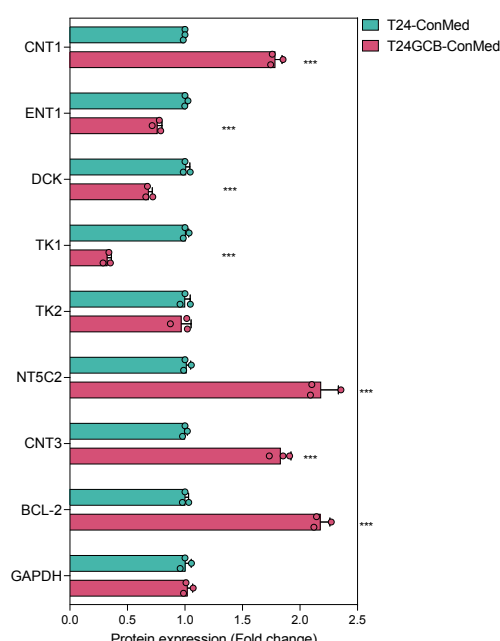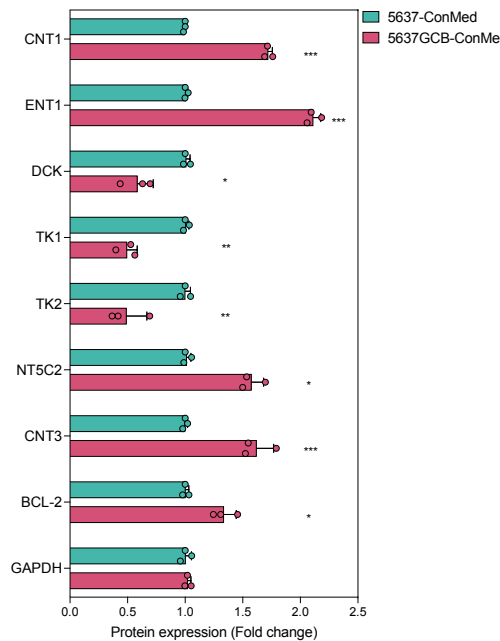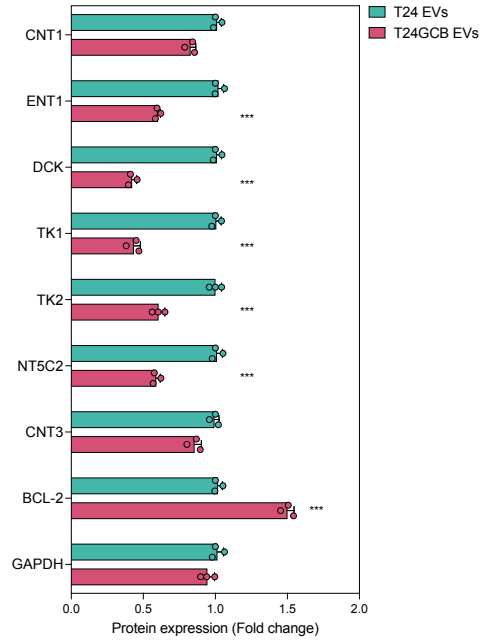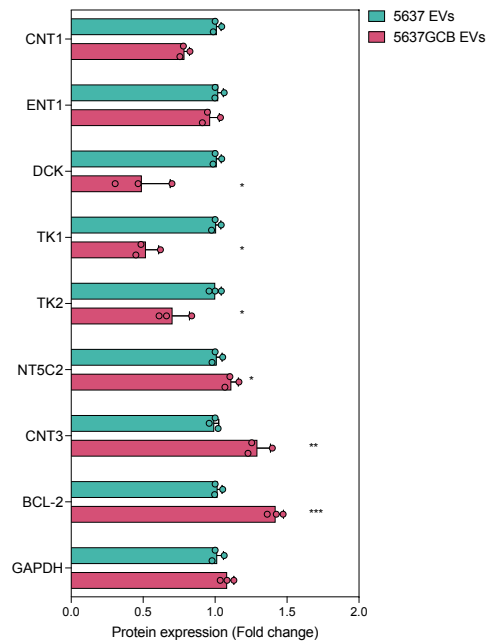

Fig 4A

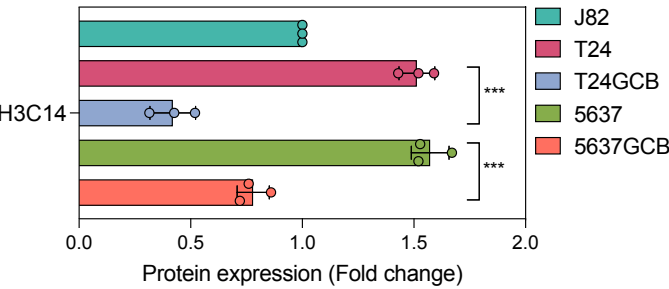

Fig 4B

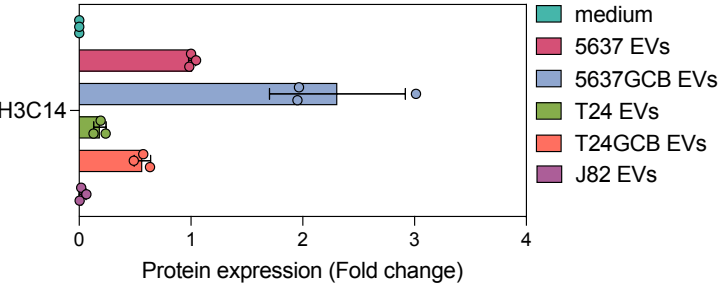

Fig 4C

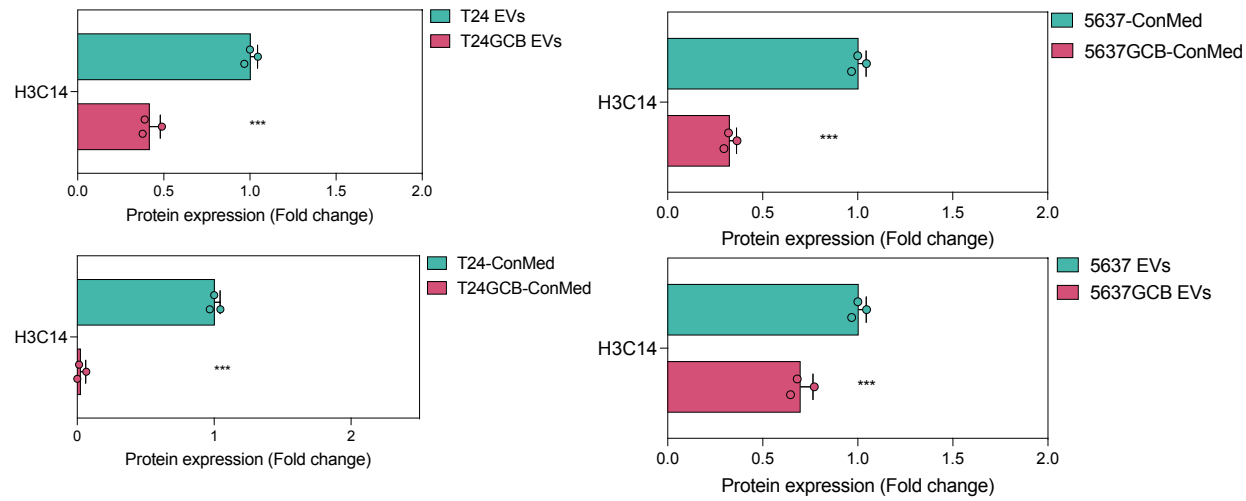

Fig 4P

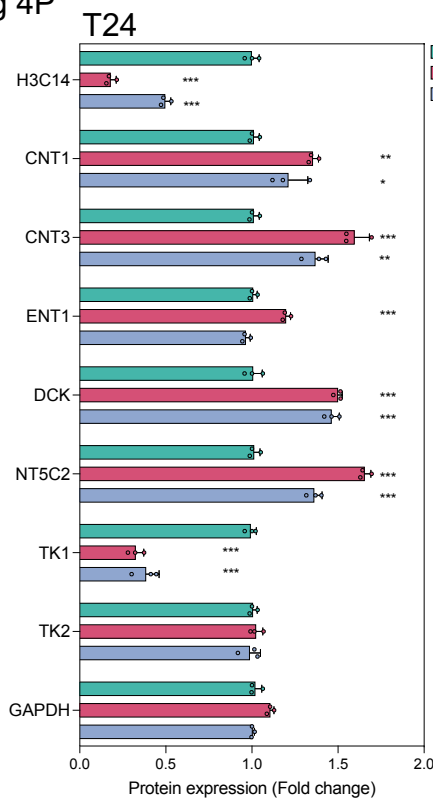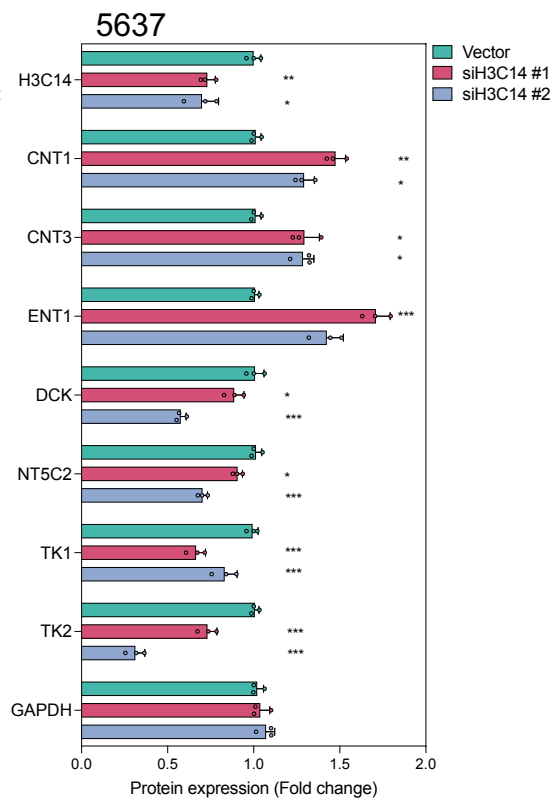

Fig 5D

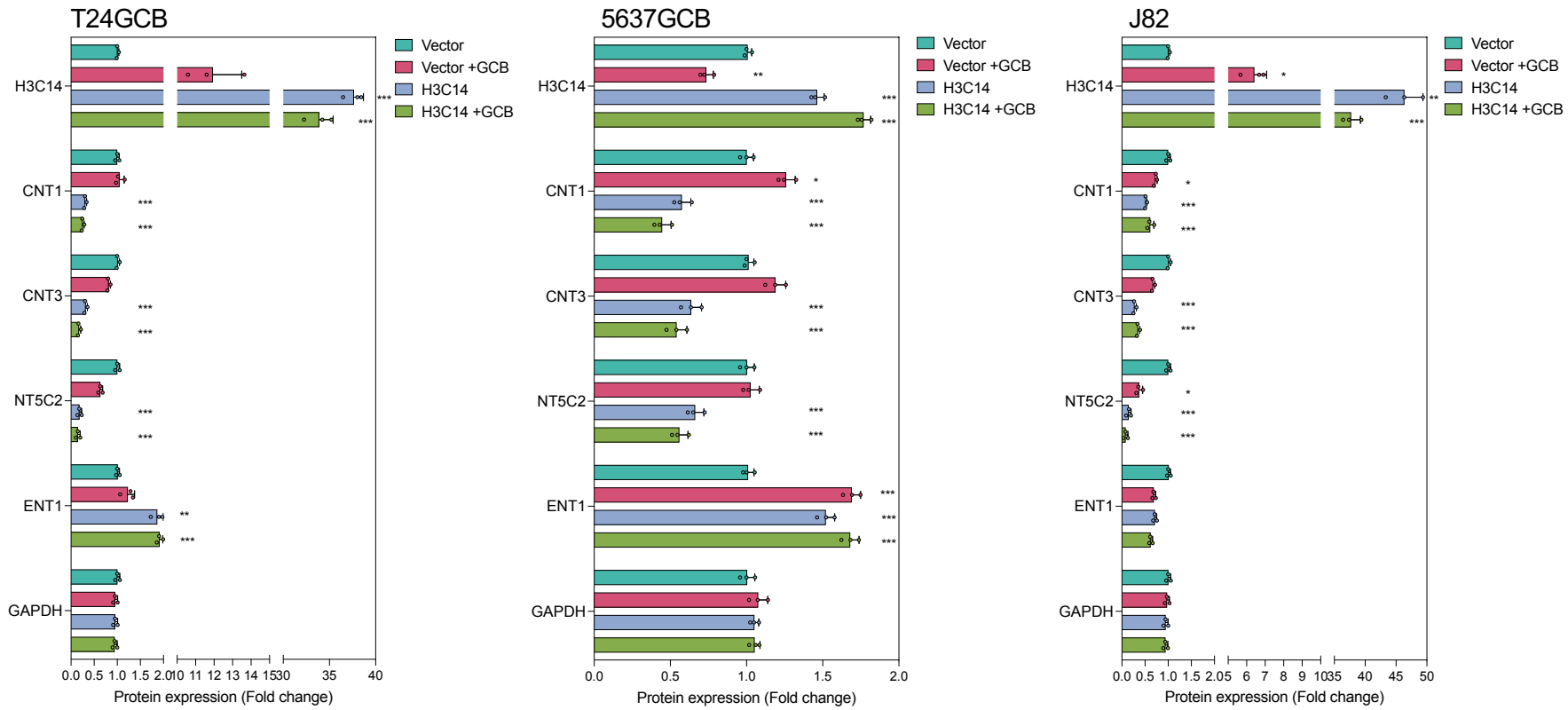

Fig 6J

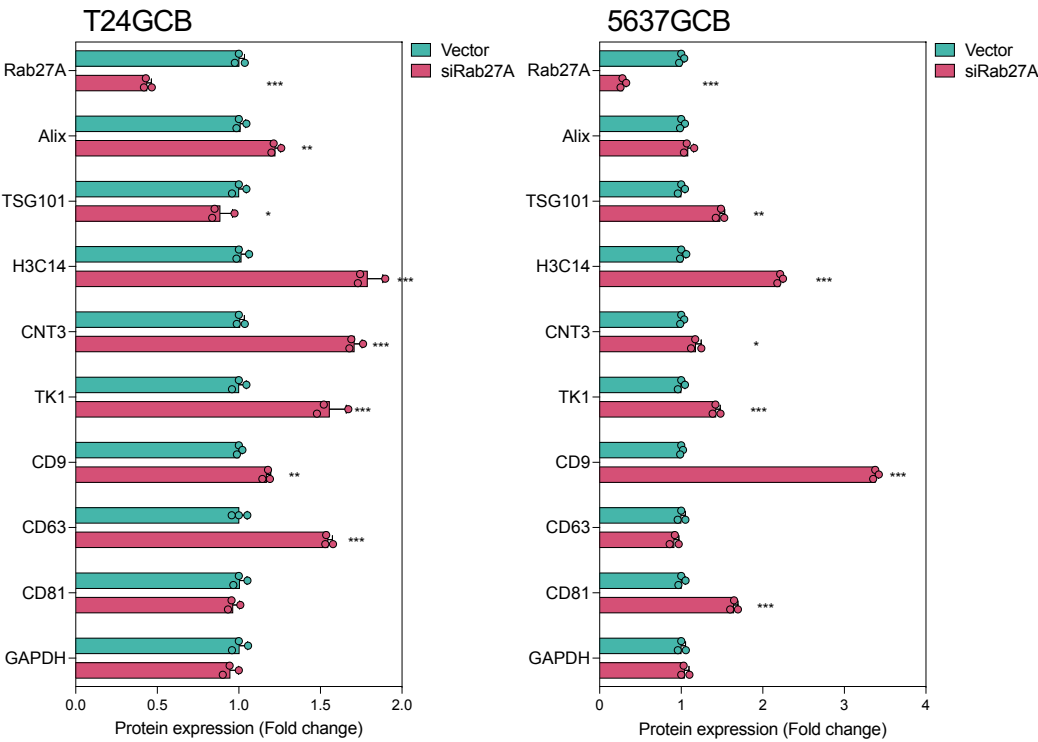

Fig 6N

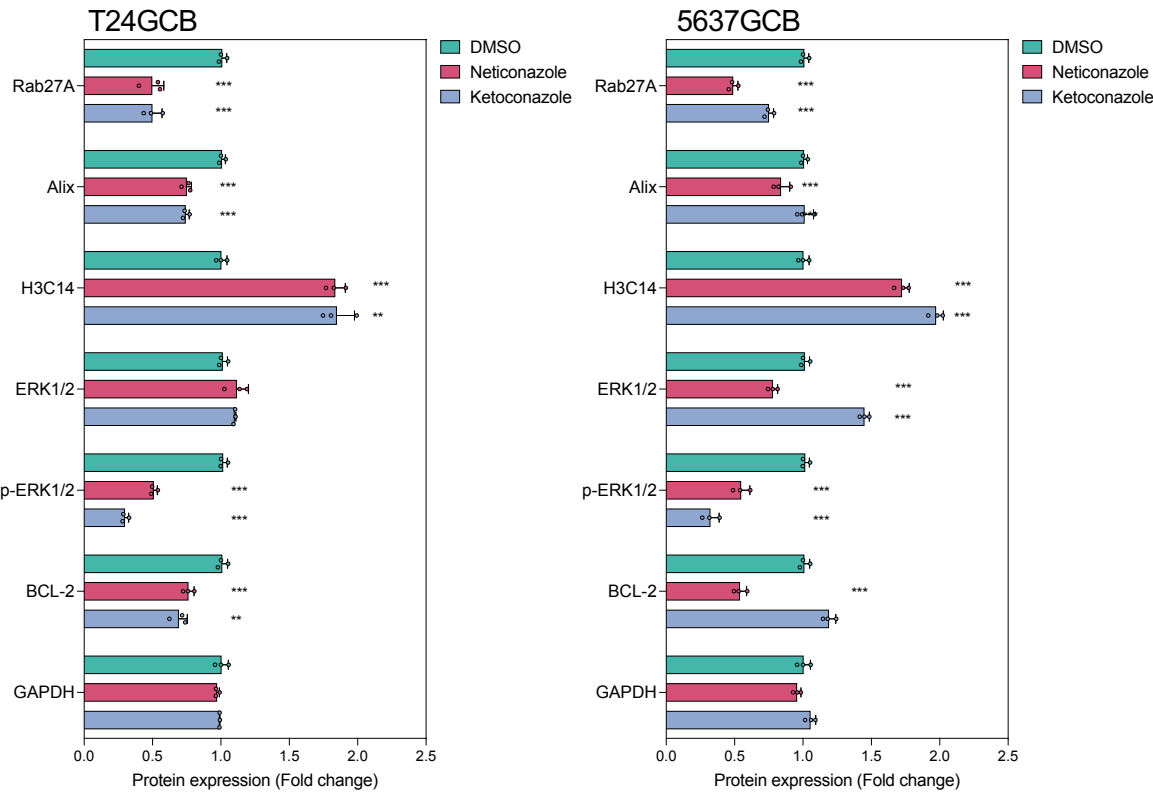

Fig 6R

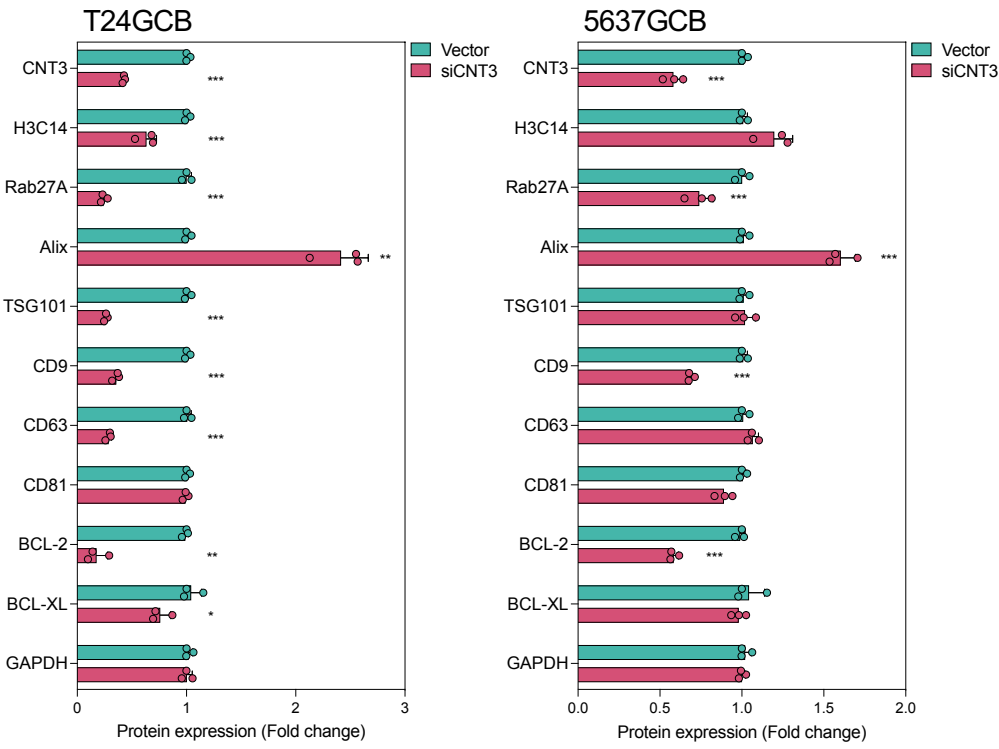

Supplement: Supplementary file 2 — Supplementary Material: jev270179‐sup‐0002‐SuppMatt.pdf [file JEV2-14-e70179-s004.pdf]
