# Supplementary material for: Extracellular Vesicle‐Mediated Regulation of H3C14 Contributes to Gemcitabine Resistance in Bladder Cancer
Source: J Extracell Vesicles. 2025 Oct 29;14(11):e70179. doi: 10.1002/jev2.70179 (PMC12570045; doi:10.1002/jev2.70179)

Fig. 1I

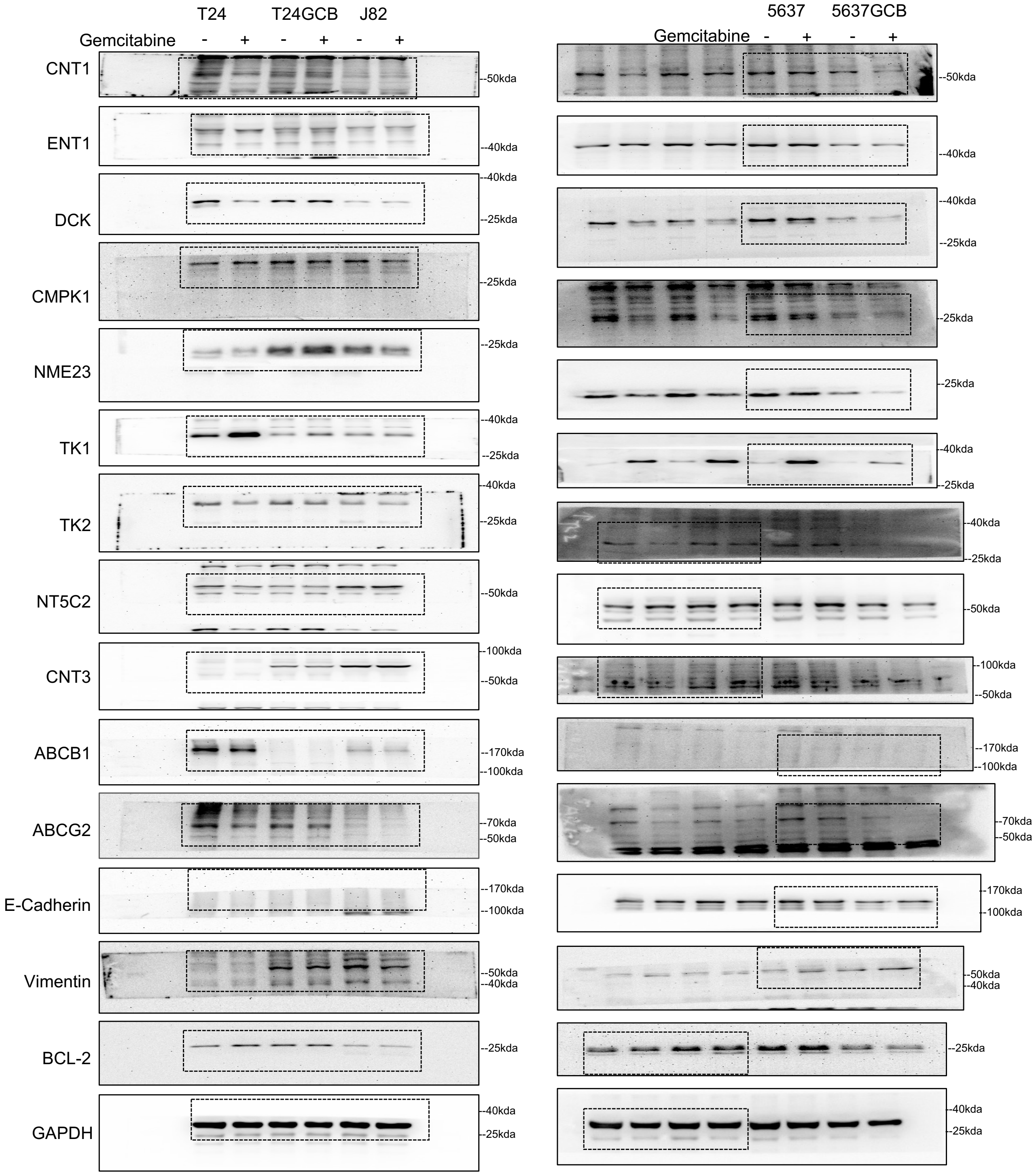

T24
T24GCB
J82
5637
5637GCB
Gemcitabine - + - + - +
Gemcitabine - + - +
CNT1
ENT1
DCK
CMPK1
NME23
TK1
TK2
NT5C2
CNT3
ABCB1
ABCG2
E-Cadherin
Vimentin
BCL-2
GAPDH
--50kda
--40kda
--25kda
--100kda
--170kda
--70kda

Fig. 2F

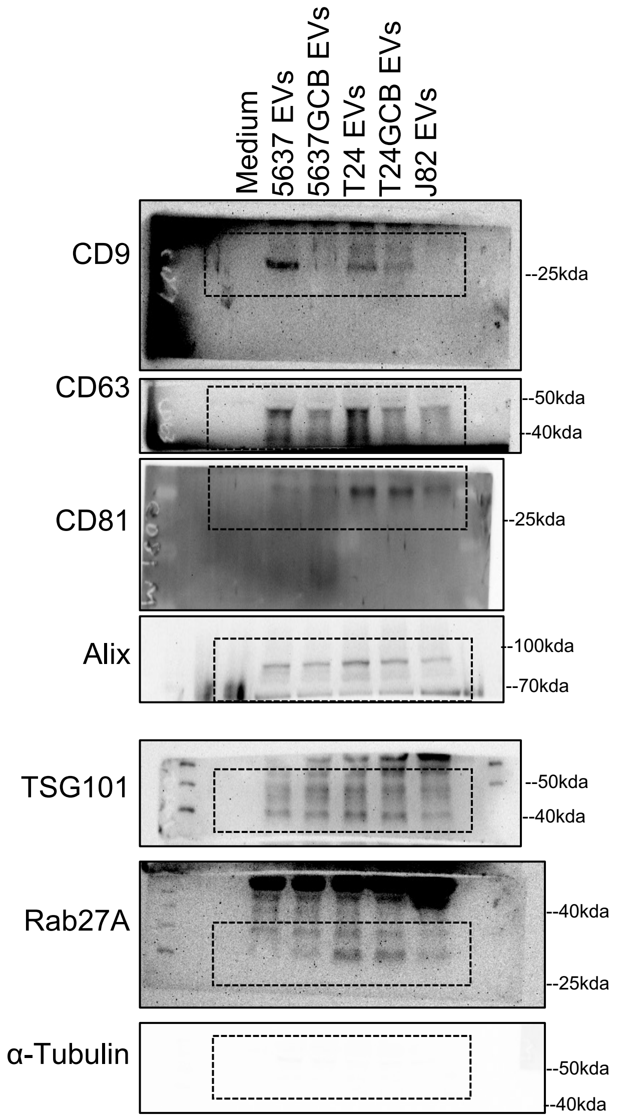
Medium
5637 EVs
5637GCB EVs
T24 EVs
T24GCB EVs
J82 EVs
CD9
--25kda
CD63
--50kda
--40kda
CD81
--25kda
Alix
--100kda
--70kda
TSG101
--50kda
--40kda
Rab27A
--40kda
--25kda
α-Tubulin
--50kda
--40kda

Fig. 4B

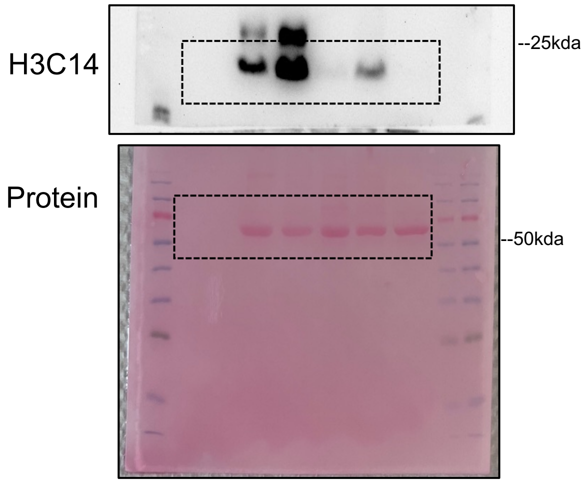
H3C14
--25kda
Total Protein
--50kda

Fig. 2J_T24

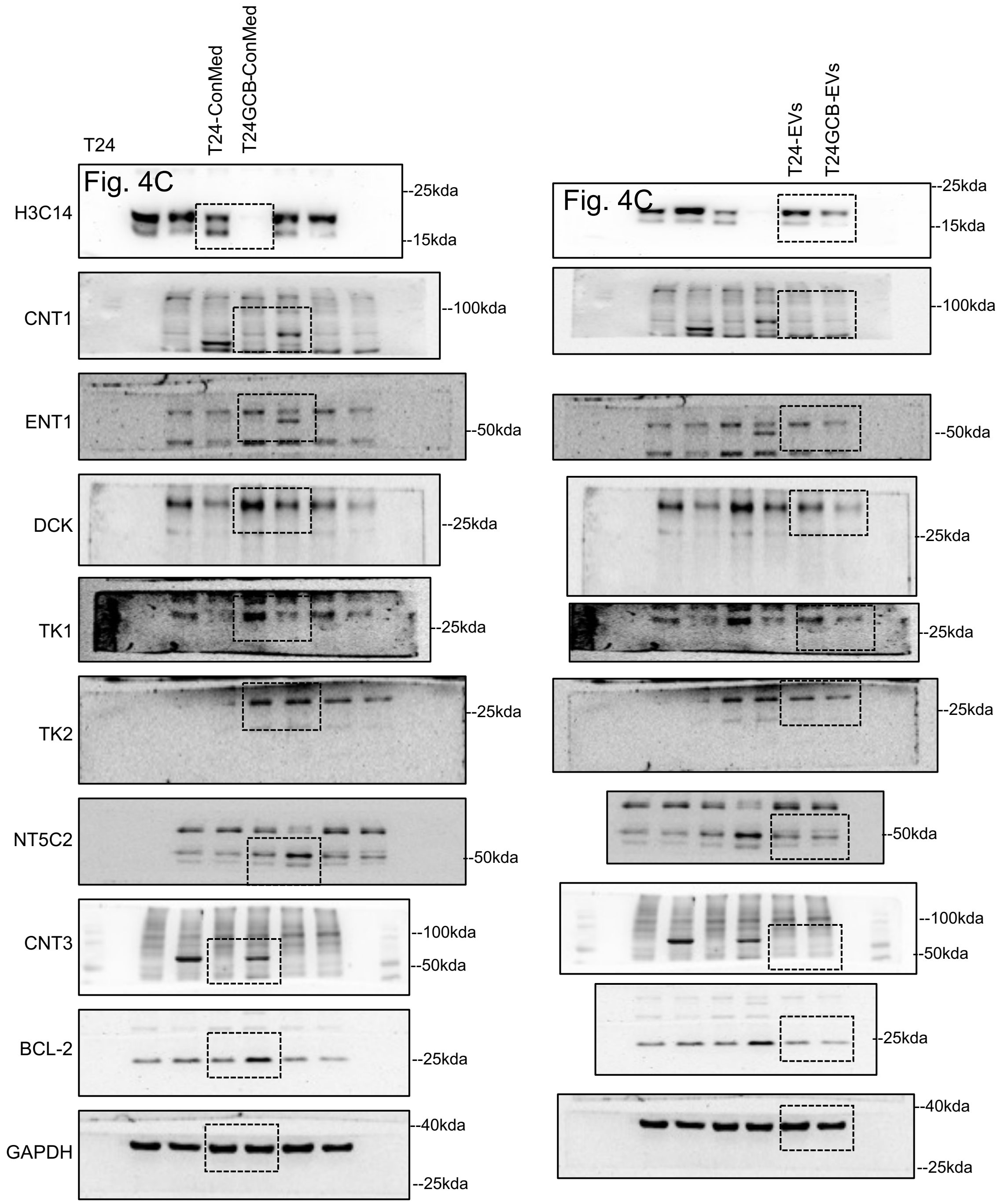
T24
T24-ConMed
T24GCB-ConMed
T24-EVs
T24GCB-EVs
Fig. 4C
Fig. 4C
H3C14
CNT1
ENT1
DCK
TK1
TK2
NT5C2
CNT3
BCL-2
GAPDH
--25kda
--15kda
--100kda
--50kda
--40kda

Fig. 2J_5637

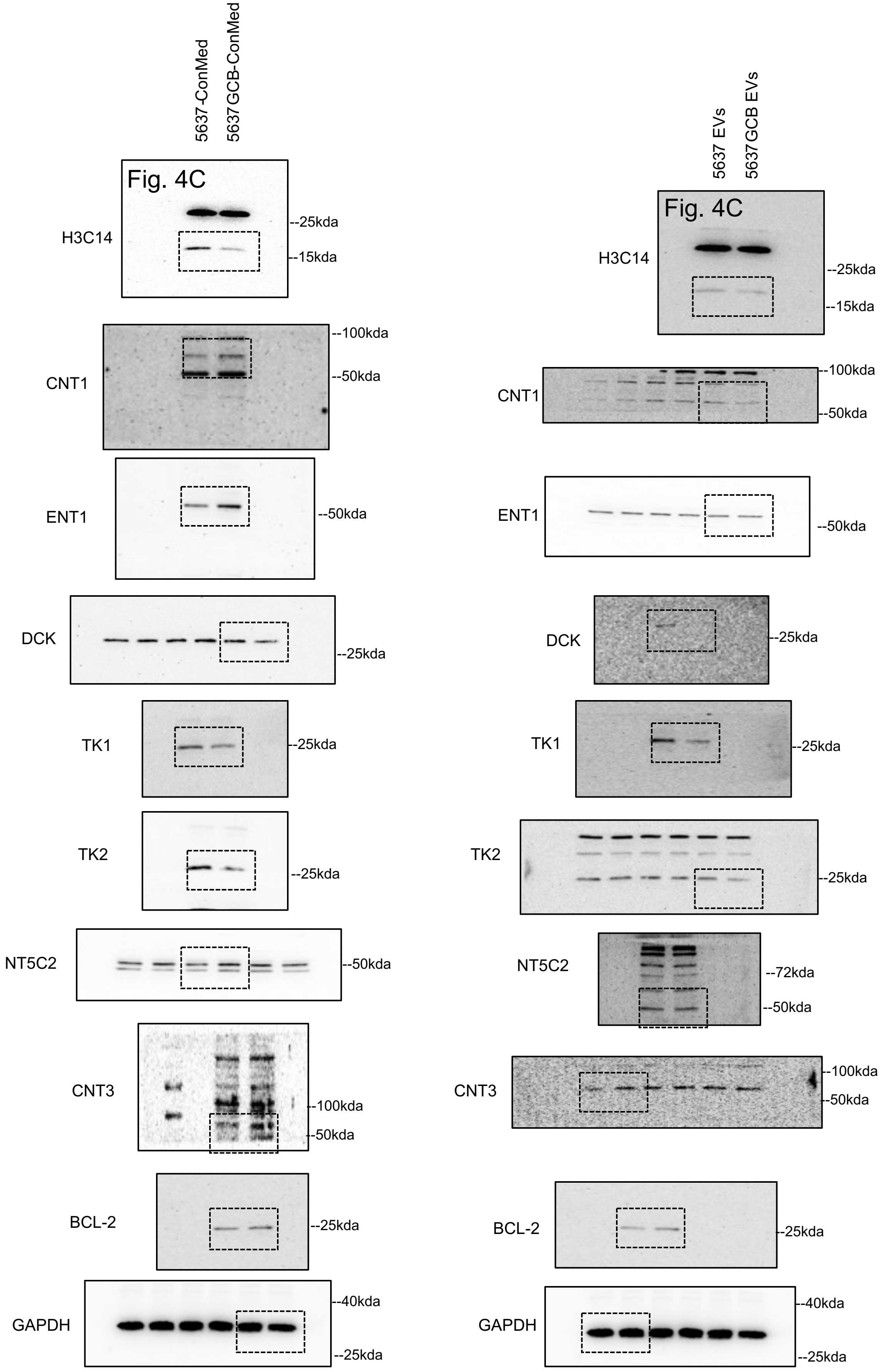

5637-ConMed
5637GCB-ConMed
5637 EVs
5637GCB EVs
Fig. 4C
H3C14
--25kda
--15kda
CNT1
--100kda
--50kda
ENT1
--50kda
DCK
--25kda
TK1
--25kda
TK2
--25kda
NT5C2
--72kda
--50kda
CNT3
--100kda
--50kda
BCL-2
--25kda
GAPDH
--40kda
--25kda

Fig. 4A

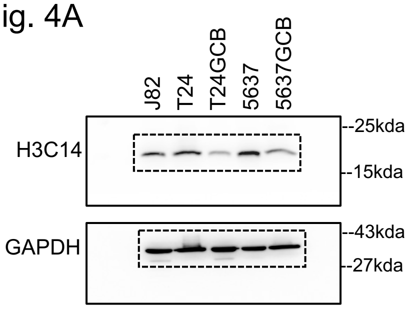
J82
T24
T24GCB
5637
5637GCB
H3C14
--25kda
--15kda
GAPDH
--43kda
--27kda

Fig. 4B

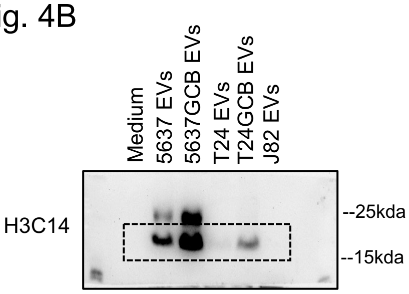
Medium
5637 EVs
5637GCB EVs
T24 EVs
T24GCB EVs
J82 EVs
H3C14
--25kda
--15kda

Fig. 4P

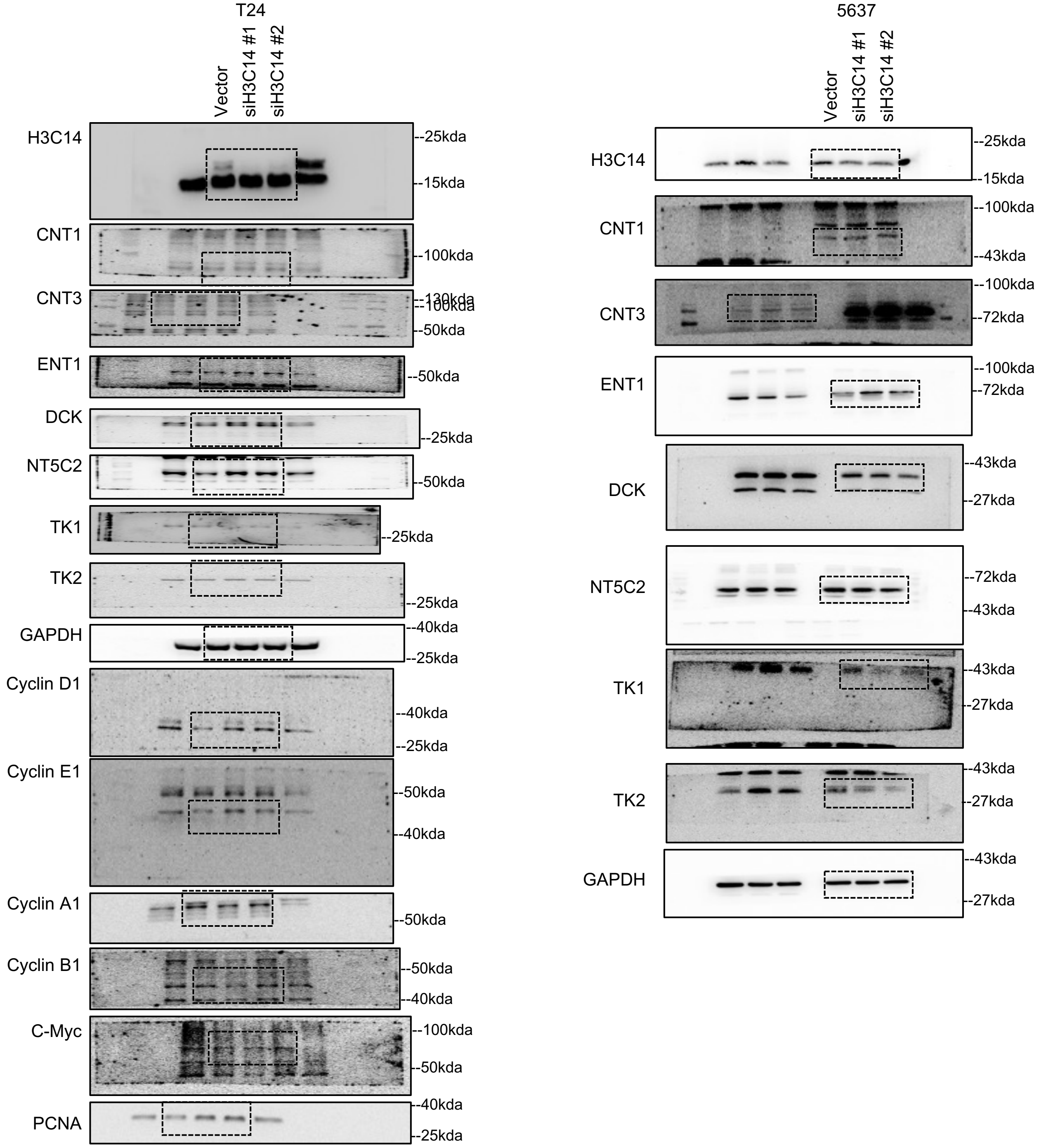
T24
Vector
siH3C14 #1
siH3C14 #2
H3C14
--25kda
--15kda
CNT1
--100kda
CNT3
--130kda
--100kda
--50kda
ENT1
--50kda
DCK
--25kda
NT5C2
--50kda
TK1
--25kda
TK2
--25kda
GAPDH
--40kda
--25kda
Cyclin D1
--40kda
--25kda
Cyclin E1
--50kda
--40kda
Cyclin A1
--50kda
Cyclin B1
--50kda
--40kda
C-Myc
--100kda
--50kda
PCNA
--40kda
--25kda
5637
Vector
siH3C14 #1
siH3C14 #2
H3C14
--25kda
--15kda
CNT1
--100kda
--43kda
CNT3
--100kda
--72kda
ENT1
--100kda
--72kda
DCK
--43kda
--27kda
NT5C2
--72kda
--43kda
TK1
--43kda
--27kda
TK2
--43kda
--27kda
GAPDH
--43kda
--27kda

Fig. 5I

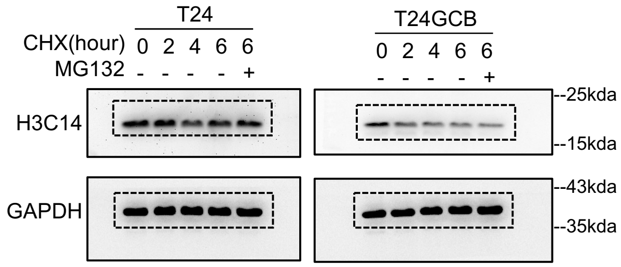
T24
CHX(hour) 0 2 4 6 6
MG132 - - - - +
T24GCB
0 2 4 6 6
- - - - +
H3C14
GAPDH
--25kda
--15kda
--43kda
--35kda

Fig. 5H

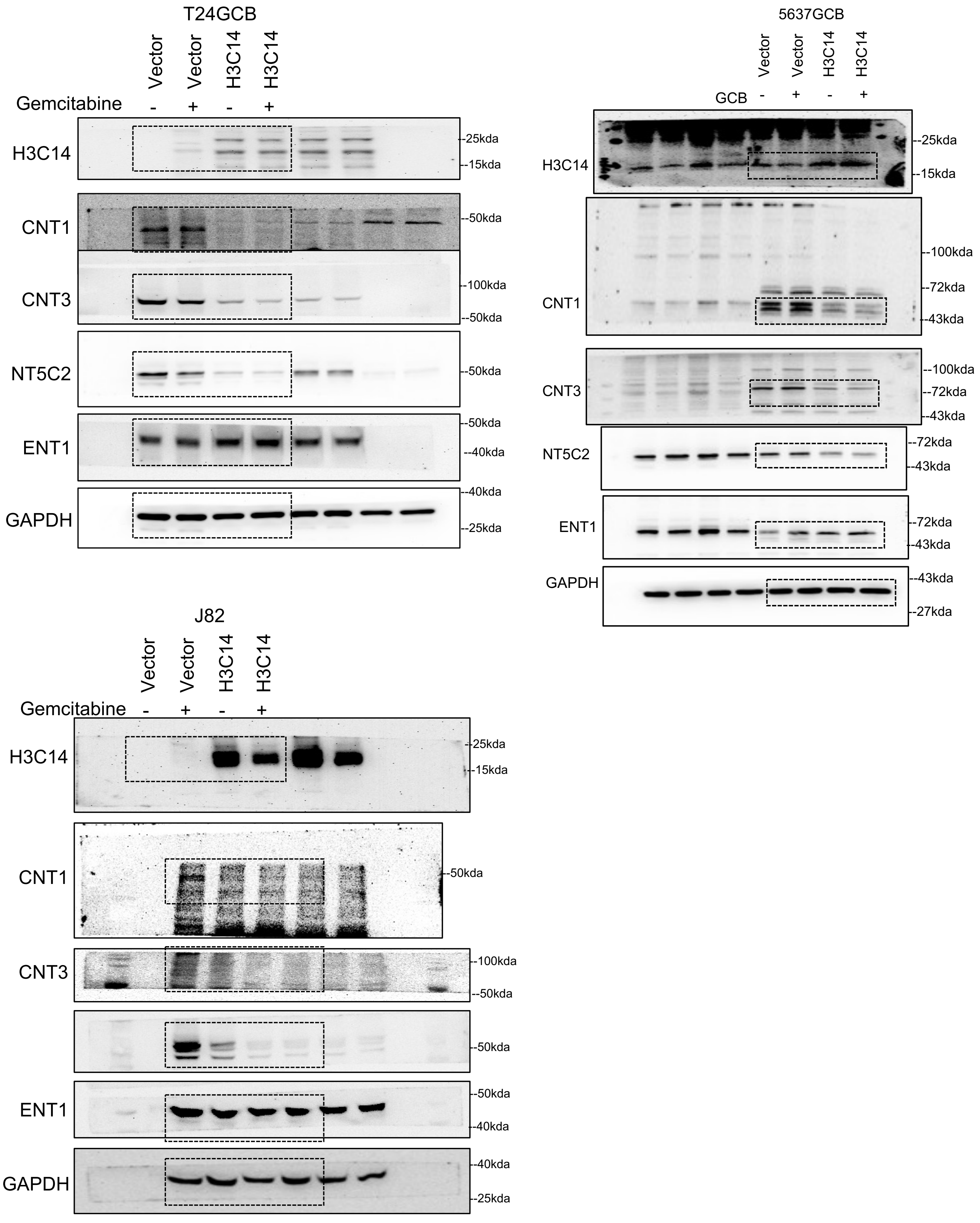
T24GCB
Vector
Vector
H3C14
H3C14
Gemcitabine
-
+
-
+
H3C14
CNT1
CNT3
NT5C2
ENT1
GAPDH
--25kda
--15kda
--50kda
--100kda
--50kda
--50kda
--50kda
--40kda
--40kda
--25kda
5637GCB
Vector
Vector
H3C14
H3C14
GCB
-
+
-
+
H3C14
CNT1
CNT3
NT5C2
ENT1
GAPDH
--25kda
--15kda
--100kda
--72kda
--43kda
--100kda
--72kda
--43kda
--72kda
--43kda
--72kda
--43kda
--43kda
--27kda
J82
Vector
Vector
H3C14
H3C14
Gemcitabine
-
+
-
+
H3C14
CNT1
CNT3
ENT1
GAPDH
--25kda
--15kda
--50kda
--100kda
--50kda
--50kda
--50kda
--40kda
--40kda
--25kda

Fig. 6G

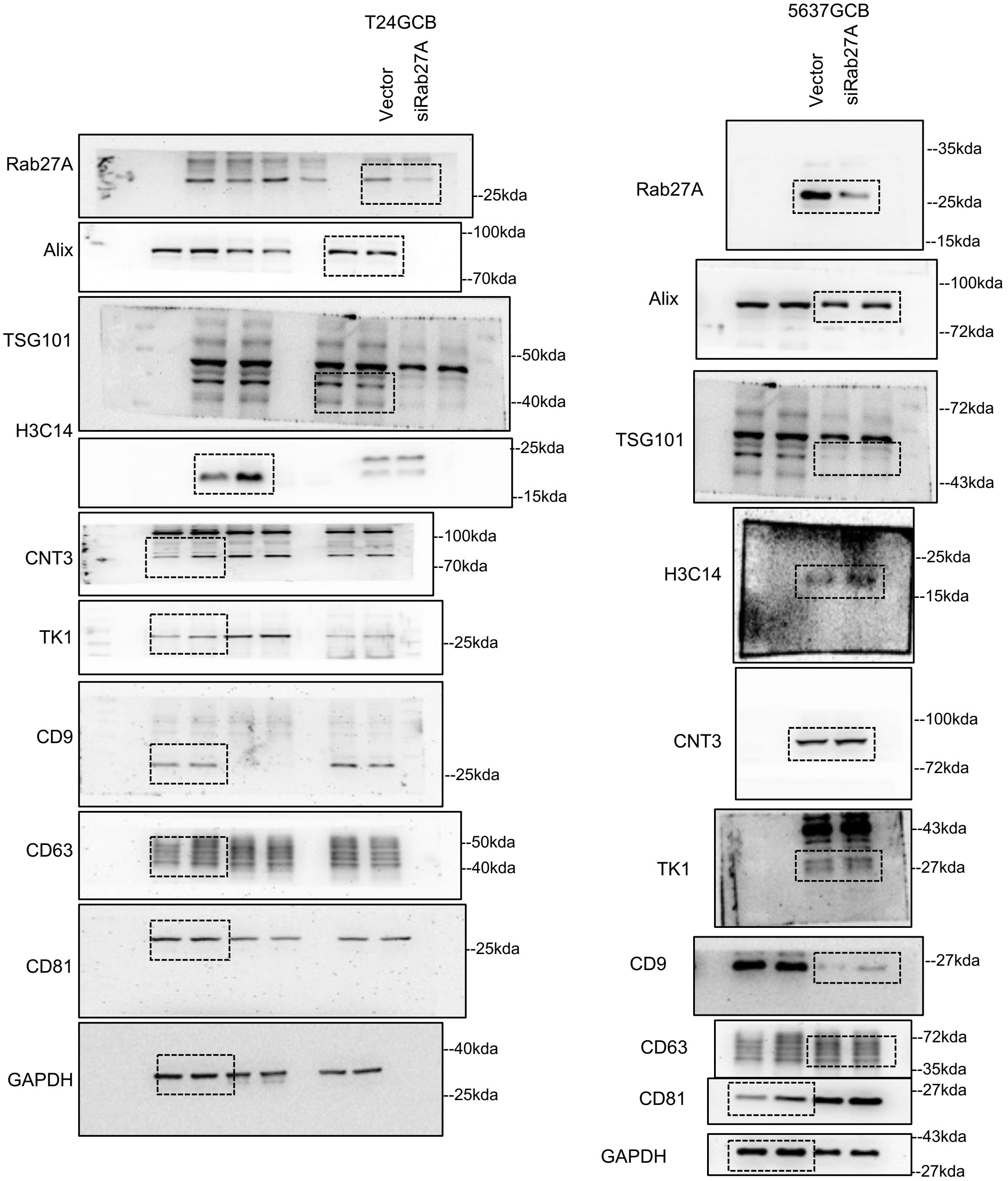
T24GCB
Vector
siRab27A
Rab27A
--25kda
Alix
--100kda
--70kda
TSG101
--50kda
--40kda
H3C14
--25kda
--15kda
CNT3
--100kda
--70kda
TK1
--25kda
CD9
--25kda
CD63
--50kda
--40kda
CD81
--25kda
GAPDH
--40kda
--25kda
5637GCB
Vector
siRab27A
Rab27A
--35kda
--25kda
--15kda
Alix
--100kda
--72kda
TSG101
--72kda
--43kda
H3C14
--25kda
--15kda
CNT3
--100kda
--72kda
TK1
--43kda
--27kda
CD9
--27kda
CD63
--72kda
--35kda
CD81
--27kda
GAPDH
--43kda
--27kda

Fig. 6K

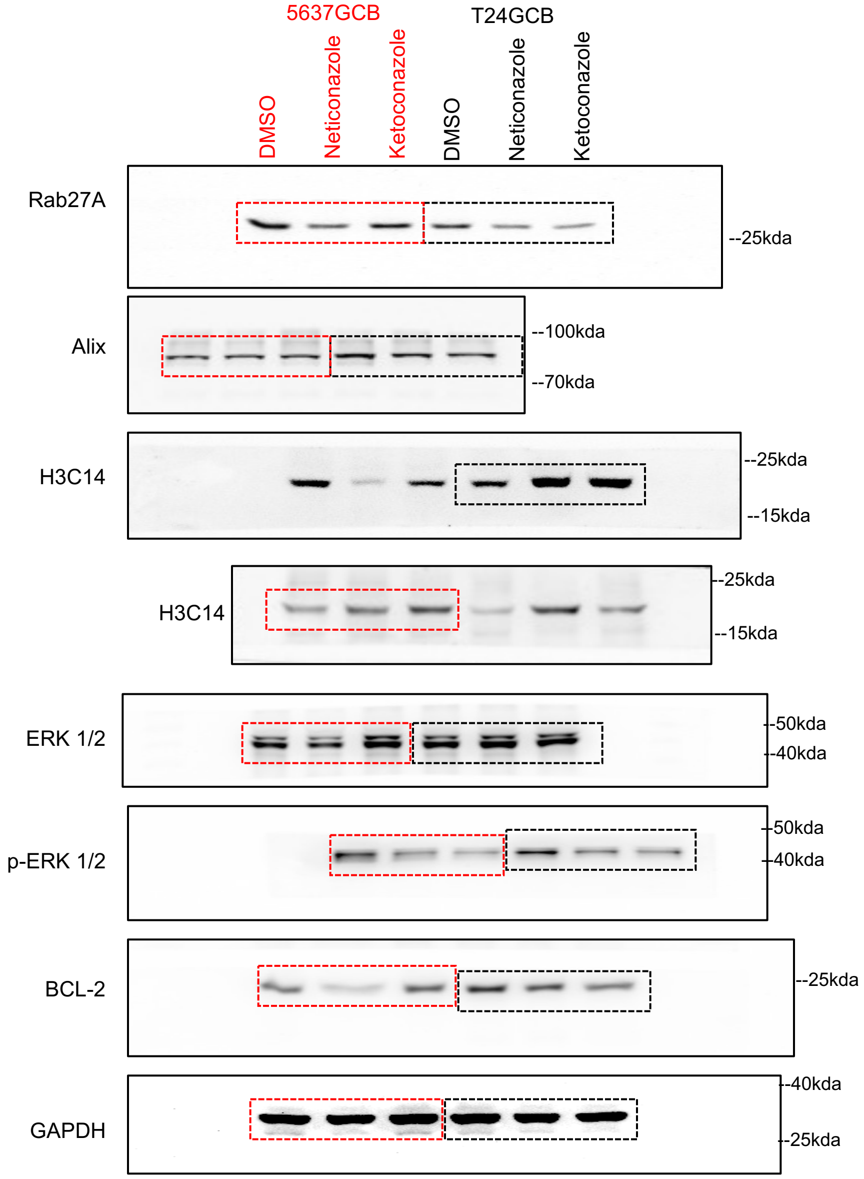

5637GCB
T24GCB
DMSO
Neticonazole
Ketoconazole
DMSO
Neticonazole
Ketoconazole
Rab27A
--25kda
Alix
--100kda
--70kda
H3C14
--25kda
--15kda
H3C14
--25kda
--15kda
ERK 1/2
-50kda
-40kda
p-ERK 1/2
-50kda
-40kda
BCL-2
--25kda
GAPDH
-40kda
-25kda

Fig. 6R

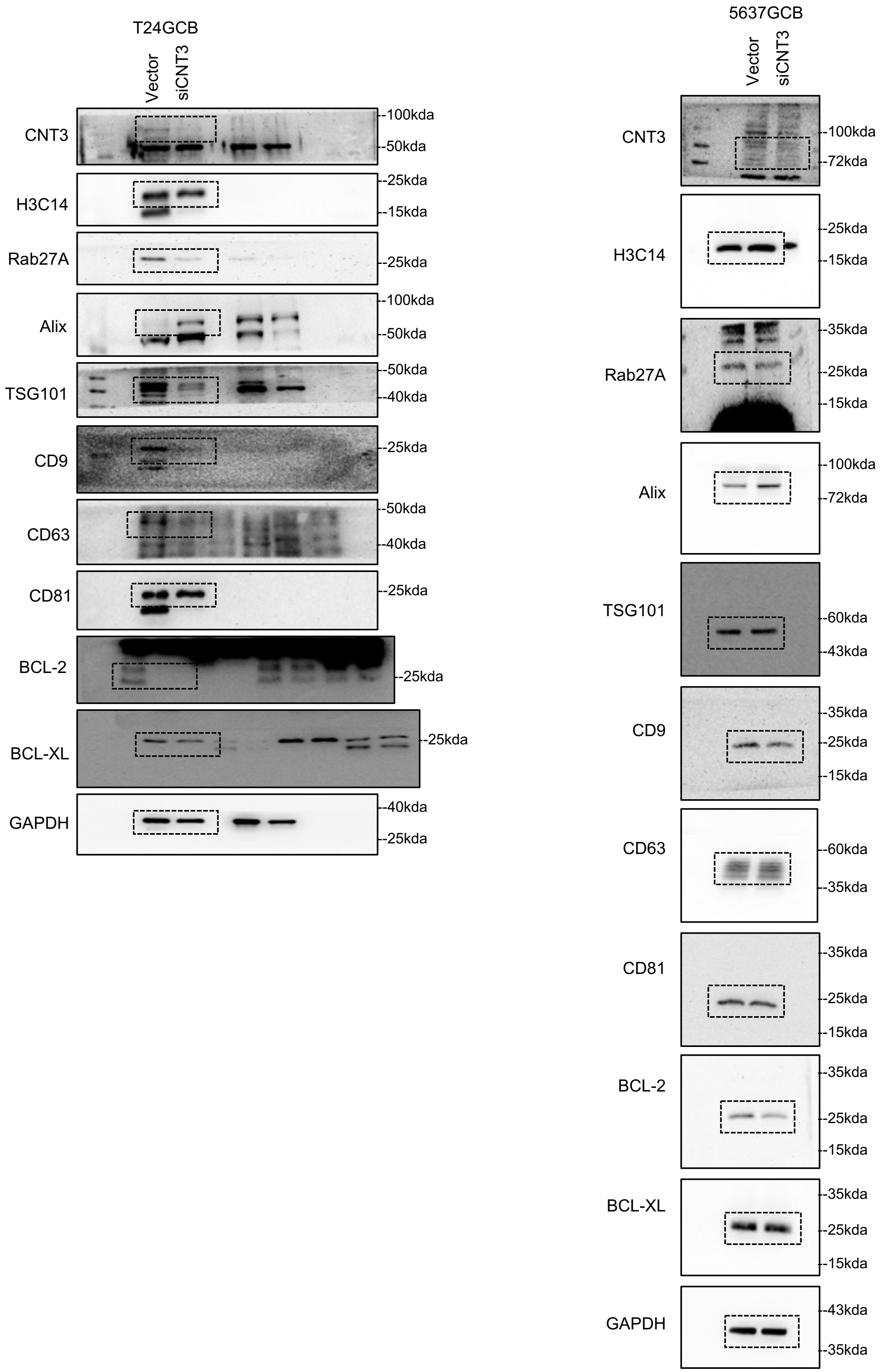
T24GCB
Vector
siCNT3
CNT3
H3C14
Rab27A
Alix
TSG101
CD9
CD63
CD81
BCL-2
BCL-XL
GAPDH
-100kda
-50kda
-25kda
-15kda
-25kda
-100kda
-50kda
-50kda
-40kda
-25kda
-50kda
-40kda
-25kda
-25kda
-25kda
-40kda
-25kda
5637GCB
Vector
siCNT3
CNT3
H3C14
Rab27A
Alix
TSG101
CD9
CD63
CD81
BCL-2
BCL-XL
GAPDH
-100kda
-72kda
-25kda
-15kda
-35kda
-25kda
-15kda
-100kda
-72kda
-60kda
-43kda
-35kda
-25kda
-15kda
-60kda
-35kda
-35kda
-25kda
-15kda
-35kda
-25kda
-15kda
-35kda
-25kda
-15kda
-43kda
-35kda

Fig. 7D

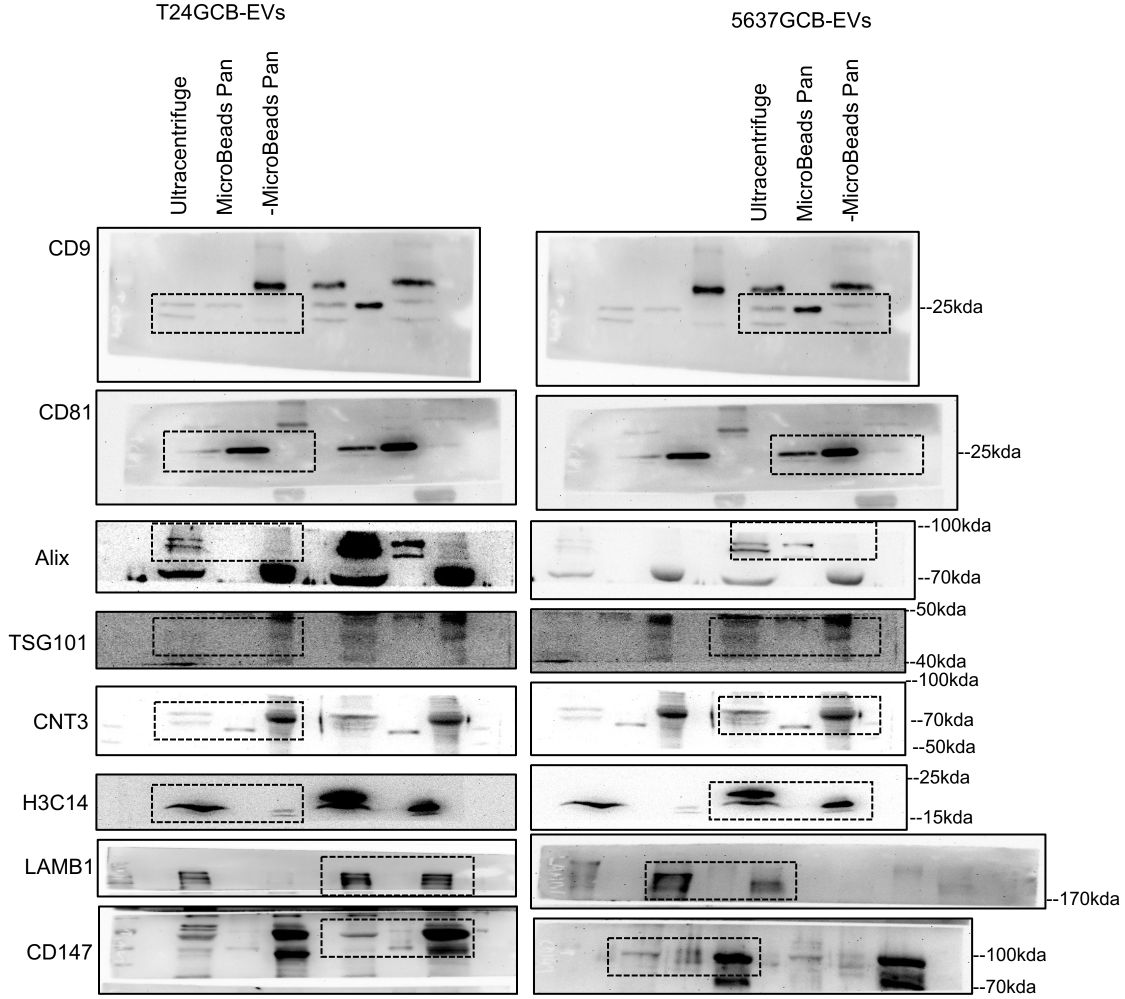

T24GCB-EVs
5637GCB-EVs
Ultracentrifuge
MicroBeads Pan
-MicroBeads Pan
CD9
CD81
Alix
TSG101
CNT3
H3C14
LAMB1
CD147
--25kda
--25kda
--100kda
--70kda
--50kda
--40kda
--100kda
--70kda
--50kda
--25kda
--15kda
--170kda
--100kda
--70kda

Fig. 7E

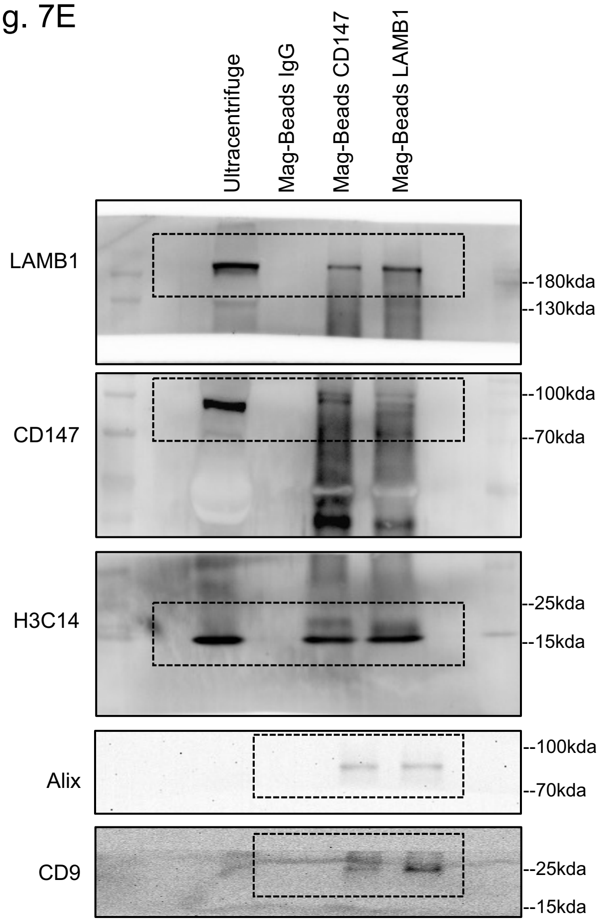
Ultracentrifuge
Mag-Beads IgG
Mag-Beads CD147
Mag-Beads LAMB1
LAMB1
CD147
H3C14
Alix
CD9
--180kda
--130kda
--100kda
--70kda
--25kda
--15kda
--100kda
--70kda
--25kda
--15kda

Supplementary Figure 2

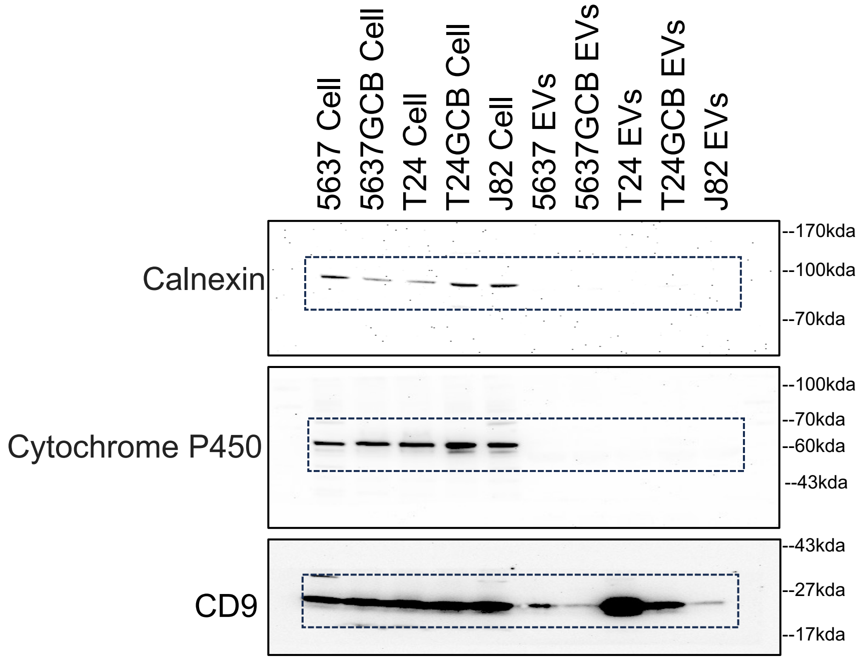

Supplementary Figure 5

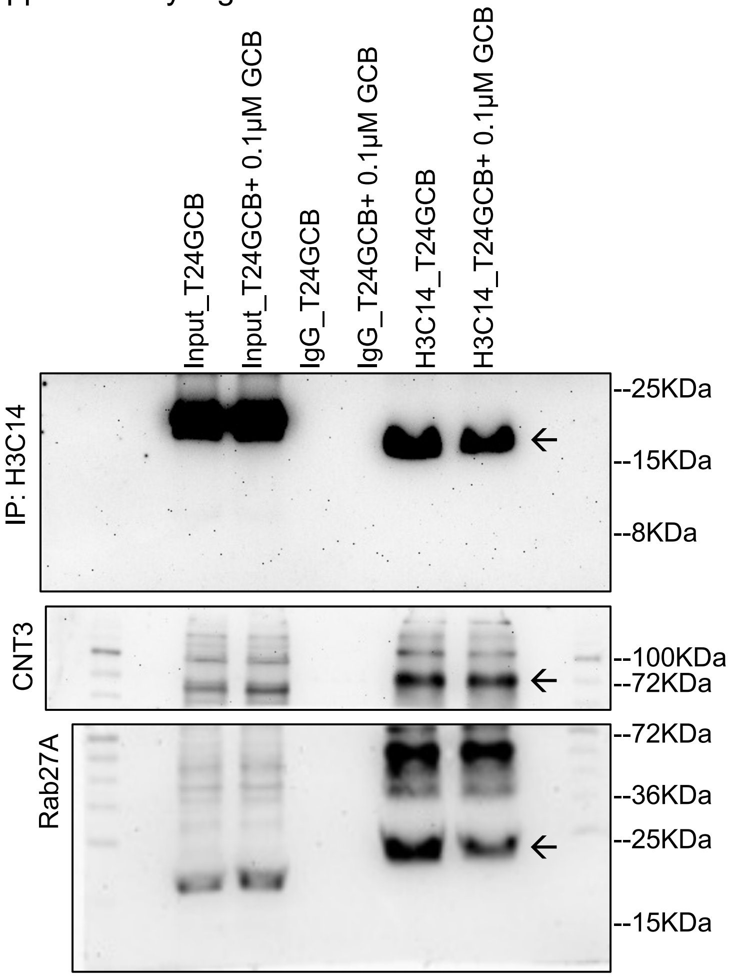

Supplementary Figure 8

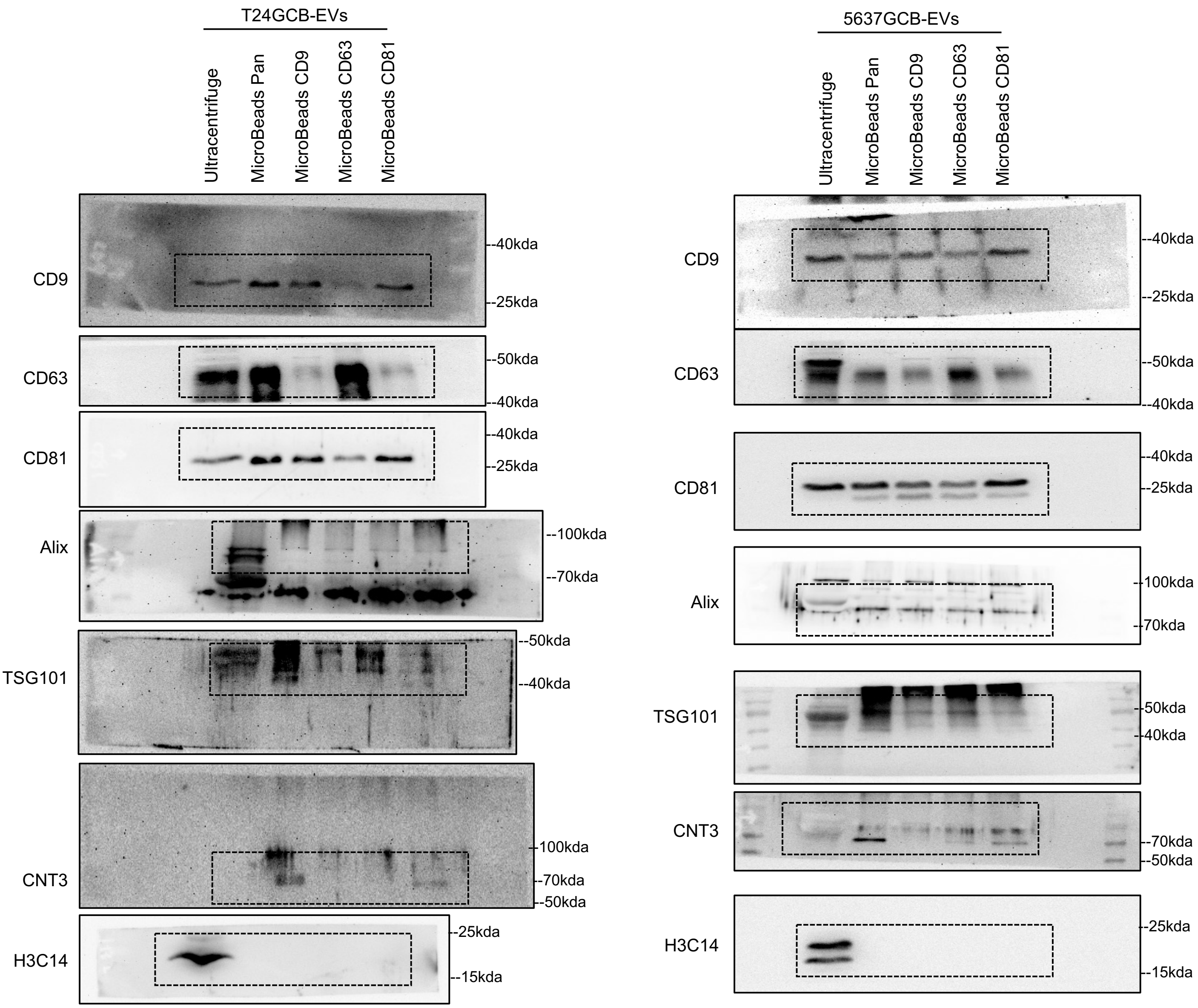

Supplement: Supplementary file 3 — Supplementary Material: jev270179‐sup‐0003‐SuppMat.pdf [file JEV2-14-e70179-s005.pdf]
