# Supplementary material for: Extracellular Vesicle‐Mediated Regulation of H3C14 Contributes to Gemcitabine Resistance in Bladder Cancer
Source: J Extracell Vesicles. 2025 Oct 29;14(11):e70179. doi: 10.1002/jev2.70179 (PMC12570045; doi:10.1002/jev2.70179)
Supplement: Supplementary file 4 — Supplementary Material: jev270179‐sup‐0004‐SuppMat.docx [file JEV2-14-e70179-s003.docx]

**Supplementary Figure 1. Quantitative Analysis of GCB in EV Samples using HPLC-UV.**

(A) A seven-point standard calibration curve was constructed using GCB concentrations of 0.01, 0.05, 0.1, 0.5, 1, 5, and 10 μM to evaluate the sensitivity and quantification capability of the developed HPLC-UV method.

(B–C) HPLC-UV chromatograms of six experimental conditions: SP1 (J82 EVs spiked with 0.1 μM GCB), SP2 (J82 EVs spiked with 0.05 μM GCB), SP3 (T24 EVs), SP4 (T24GCB EVs), SP5 (5637 EVs), and SP6 (5637GCB EVs).

(D) Quantification results of GCB concentration in each sample.

(E) Among the spiked samples, SP1 and SP2 yielded measurable GCB concentrations of 0.1025 μM and 0.0437 μM, respectively. In contrast, SP3–SP6 exhibited no detectable GCB signal above the quantification threshold (0.01 μM) and were reported as not detected (ND).

**Supplementary Figure 2. Validation of EV purity by negative markers of EVs: Calnexin and Cytochrome P450.**

To confirm the purity of isolated EVs in accordance with the latest MISEV guidelines, Western blot analysis was performed to examine the absence of non-EV contaminants. Calnexin and Cytochrome P450 were used as negative controls. EV samples from five sources—5637 EVs, 5637GCB EVs, T24 EVs, T24GCB EVs, and J82 EVs showed no detectable expression of either Calnexin or Cytochrome P450, supporting the specificity and purity of the EV isolation. CD9 served as a positive EV marker.

**Supplementary Figure 3. Uptake of fluorescence-labeled EVs by GCB-sensitive bladder cancer cells.**

(A, B) Representative fluorescence microscopy images of (A) T24 and (B) 5637 cells incubated for 6 h with EVs derived from GCB-sensitive (T24-EVs, 5637-EVs) or GCB-resistant (T24GCB-EVs, 5637GCB-EVs) bladder cancer cells. EVs were pre-labeled with CD9-APC, CD63-FITC, and CD81-PE antibodies. Scale bar: 10 µm.

(C, D) Imaging flow cytometry analysis quantifies the uptake of EVs labeled with CD9-APC, CD63-FITC, and CD81-PE antibodies by (C) T24 and (D) 5637 cells. Data are shown as the percentage of EV-positive cells for each marker. GCB, gemcitabine; EVs, extracellular vesicles

**Supplementary Figure 4. Co-immunoprecipitation analysis of H3C14 with Rab27A and CNT3 in T24GCB cells.**

Western blot analysis of co-immunoprecipitation assays performed in T24GCB cells and T24GCB cells treated with 0.1 µM GCB using anti-H3C14 antibody for immunoprecipitation.

**Supplementary Figure 5. Imaging flow cytometry analysis revealed co-localization of Rab27A and H3.2 (H3C14) upon GCB treatment.**

(A) Representative imaging flow cytometry revealed co-localization of Rab27A and H3.2 (H3C14) in T24, T24GCB, and J82 cells treated with 0.1 µM GCB or left untreated for 24 h. Cells were stained with Rab27A-APC and H3C14-FITC antibodies, and nuclei were stained with DAPI prior to image acquisition using ImageStreamX. Following GCB treatment, T24 cells exhibited increased Rab27A expression and reduced nuclear H3.2 (H3C14) levels. In contrast, T24GCB and J82 cells displayed high Rab27A levels regardless of GCB treatment, with Rab27A detected in the nucleus and co-localized with H3.2 (H3C14).

(B) Quantification of Rab27A nuclear localization and Rab27A–H3.2 (H3C14) co-localization percentages in T24, T24GCB, and J82 cells (n = 3 per group). A significant increase was observed in Rab27A nuclear entry and co-localization with H3.2 (H3C14) in GCB-resistant cells compared to GCB-sensitive cells. Data are presented as mean ± SEM. *p<0.05, **p<0.01 by an unpaired two-tailed Student’s t-test. GCB, gemcitabine; DAPI, 4',6-diamidino-2-phenylindole

**Supplementary Figure 6. GO enrichment analysis of Transport-EVs and Excretion-EVs using IPA.**

(A) GO Biological Process (Complete) enrichment analysis of proteins enriched in Transport-EVs identified pathways associated with cytoskeletal organization, epithelial barrier formation, immune response, and viral entry, including intermediate filament organization, keratinization, establishment of the skin barrier, keratinocyte differentiation, and humoral immune response mediated by circulating immunoglobulin.

(B) GO Cellular Component (Complete) analysis of Transport-EVs revealed enrichment in structures such as the integrin alpha3-beta1 complex, cornified envelope, keratin filament, blood microparticle, and melanosome.

(C) GO Molecular Function (Complete) enrichment analysis of proteins enriched in Excretion-EVs revealed functional associations with structural molecule activity, integrin binding, virus receptor activity, cell adhesion molecule binding, and exogenous protein binding.

(D) GO Cellular Component (Complete) analysis of Excretion-EVs demonstrated strong associations with extracellular compartments and structural scaffolds, including extracellular exosome, extracellular vesicle, keratin filament, extracellular membrane-bounded organelle, and extracellular region.

All data were derived from proteomic profiling and analyzed using IPA software. Significance was determined based on adjusted p-values and enrichment scores. GO, Gene Ontology; EVs, extracellular vesicles; IPA, Ingenuity Pathway Analysis

**Supplementary Figure 7. Validation of Transport and Excretion EV identity by FM 1-43 staining and SEM imaging.**

(A) EVs from T24GCB cells were captured using magnetic beads conjugated with IgG (negative control), CD9⁺CD63⁺CD81⁺ antibodies (Transport EVs), or CD147⁺LAMB1⁺ antibodies (Excretion EVs), followed by FM 1-43 staining and ImageStreamX analysis. FM 1-43-positive signals confirmed the membrane integrity of both Transport and Excretion EVs.

(B) Representative images from ImageStreamX showing FM 1-43 fluorescence (middle), merged signal, and side scatter (SSC) for each EV-bead complex.

(C) Scanning electron microscopy (SEM) images showing the surface of beads incubated with EVs. CD147⁺LAMB1⁺-beads (Excretion EVs) displayed clear vesicle-like structures, while IgG-beads showed no EV association. Scale bars are indicated.

**Supplementary Figure 8. Subpopulation-Specific EV Sorting and H3.2 (H3C14) Exclusion in GCB-Resistant Bladder Cancer EVs**

(A and B) Western blot analysis of EVs isolated from T24GCB and 5637GCB cells using magnetic beads targeting CD9, CD63, and CD81. CNT3 was enriched in CD81⁺ EVs, while H3.2 (H3C14) protein was not detected in CD9⁺, CD63⁺, or CD81⁺ EV fractions.

Quantification of H3.2 (H3C14) and CNT3 levels in bead-isolated EV fractions. These data support the conclusion that H3.2 (H3C14) protein was excreted by Excretion-EVs (CD147+, LAMB1+, CD9-, CD63-, or CD81-). GCB, gemcitabine; EVs, extracellular vesicles
