# Supplementary material for: Extracellular Vesicle‐Mediated Regulation of H3C14 Contributes to Gemcitabine Resistance in Bladder Cancer
Source: J Extracell Vesicles. 2025 Oct 29;14(11):e70179. doi: 10.1002/jev2.70179 (PMC12570045; doi:10.1002/jev2.70179)
Supplement: Supplementary file 5 — Supplementary Tables: jev270179‐sup‐0005‐Table.docx [file JEV2-14-e70179-s001.docx]

**Table 1. Primary antibodies and secondary antibodies used in the study**

**Table 2. Primer sequences used in the study**

| mRNA |  | Sequence |
| --- | --- | --- |
| H3C14 | Forward (5'-3') | CCTGTGTTTTGGTTCGCTAT |
|  | Reverse (5'-3') | CCTTTTGGATACGATGGAAC |
| Rab27A | Forward (5'-3') | AGAGGCCAGAGAATCCACCT |
|  | Reverse (5'-3') | CACACCGTTCCATTCGCTTC |
| CNT3 | Forward (5'-3') | ACGTGGGCTTCCAGAATGAA |
|  | Reverse (5'-3') | CCAGATGATGTGCCGAAGAGT |
| RPLP0 | Forward (5'-3') | GCAGCATCTACAACCCTGAAG |
|  | Reverse (5'-3') | CACTGGCAACATTGCGGAC |
| RPSA | Forward (5'-3') | GTGGCACCAATCTTGACTTCC |
|  | Reverse (5'-3') | GCAGGGTTTTCAATGGCAACAA |
| TTN | Forward (5'-3') | CCCCATCGCCCATAAGACAC |
|  | Reverse (5'-3') | CCACGTAGCCCTCTTGCTTC |
| RPS23 | Forward (5'-3') | GGTGCTTCTCATGCAAAAGGA |
|  | Reverse (5'-3') | GCAACCGTCATTGGGTACAAA |
